# Medicine 2.0: Social Networking, Collaboration, Participation, Apomediation, and Openness

**DOI:** 10.2196/jmir.1030

**Published:** 2008-08-25

**Authors:** Gunther Eysenbach

**Keywords:** Cooperative Behavior, Education, Information Storage and Retrieval, Interpersonal Relations, Organizational Innovation, Social Behavior, User-Computer Interface, Online Systems, Patient Education as Topic, Terminology as Topic, Medical Record Linkage, Self Care, Internet, Health Communication, Collaboration, Research

## Abstract

In a very significant development for eHealth, a broad adoption of Web 2.0 technologies and approaches coincides with the more recent emergence of Personal Health Application Platforms and Personally Controlled Health Records such as Google Health, Microsoft HealthVault, and Dossia. “Medicine 2.0” applications, services, and tools are defined as Web-based services for health care consumers, caregivers, patients, health professionals, and biomedical researchers, that use Web 2.0 technologies and/or semantic web and virtual reality approaches to enable and facilitate specifically 1) social networking, 2) participation, 3) apomediation, 4) openness, and 5) collaboration, within and between these user groups. The Journal of Medical Internet Research (JMIR) publishes a Medicine 2.0 theme issue and sponsors a conference on “How Social Networking and Web 2.0 changes Health, Health Care, Medicine, and Biomedical Research”, to stimulate and encourage research in these five areas.

## JMIR’s Theme Issue and Conference on Medicine 2.0

In the past 9 years, the *Journal of Medical Internet Research* (JMIR) has been publishing hundreds of research and opinion articles on how the Internet is changing medical practice, transforming biomedical research, and empowering health care consumers. While we have seen many new concepts and terms appear and disappear, the term “Web 2.0” (and its derivatives, for example “Web 3.0”) is increasingly entering our discussions and is likely  here to stay.

It is easy to dismiss some of the “hype” around Web 2.0 as a marketing gimmick or rhetoric geared towards attracting venture capital for Web 2.0 startups. However, most Internet researchers and developers probably also agree that recent advances in web technologies and user interfaces have greatly changed the design, appearance, stickiness, and pervasiveness of Web applications, and in many cases transformed the way users interact with them. Perhaps equally importantly, it also has changed the *expectations* of users. After some hard lessons learned from failed Web ventures which disappeared overnight taking any user-generated data with them, people expect Web applications to be open and interoperable. Improved communication between separate software applications (“mashups”) via open Web standards leads to improved collaboration and communication across applications. Social networking approaches revolutionize the way people collaborate, identify potential collaborators or friends, communicate with each other, and identify information that is relevant for them. And finally, Web 2.0 technologies such as AJAX lead to improved Web interfaces that mimic the real-time responsiveness of desktop applications within a browser window. Semantic Web applications (sometimes called Web 3.0) and 3D environments (such as Second Life) can also be seen as second generation Web technologies.

The emergence and broad adoption of Web 2.0 technologies and approaches coincides with the more recent emergence of Personal Health Application (PHA) Platforms (also called Personally Controlled Health Record [PCHR] platforms or “health record banks”) such as Google Health, Microsoft HealthVault, and Dossia, where data is—at the request of the consumer—pulled from various sources (including electronic health records). As eloquently argued by Mandl and colleagues in the *New England Journal of Medicine*, these developments represent “tectonic shifts in the health information economy” [1] with far-reaching consequences for patient involvement, as the gravity shifts away from health care providers as the sole custodian of medical data. PHA (or PCHR) platforms, “where health care consumers independently decide about subsequent disclosure [of health data]” represent nothing short of a “disruptive innovation that inverts the current approach to medical records in that they are created by and reside with patients who grant permission for their use to institutions, clinicians, researchers, public health agencies, and other users of medical information” [1]. A randomized controlled trial with the PCHR system Dossia illustrates the potential of PCHR for public health [2].

It easy to imagine that the combination of both trends—Personal Health Records combined with social networking, what I have called “PHR 2.0” [3]—may lead to a powerful new generation of health applications, where people share parts of their electronic health records with other consumers and “crowdsource” the collective wisdom of other patients and professionals. Advances in genetic medicine will further personalize and tailor health information, based on data stored in personal health records.

Finally, we are seeing developments in biomedical research (“Science 2.0”) and scholarly publishing which apply the same principles of participation and collaboration across different points along the continuum of knowledge production and dissemination.

In an attempt to foster and stimulate research in these areas, JMIR is proud to sponsor the new Medicine 2.0 congress series [4,5] and to publish this theme issue on “How Social Networking and Web 2.0 changes Health, Health Care, Medicine and Biomedical Research”.

## On the Scope and Definition of Medicine 2.0

**Figure 1:**
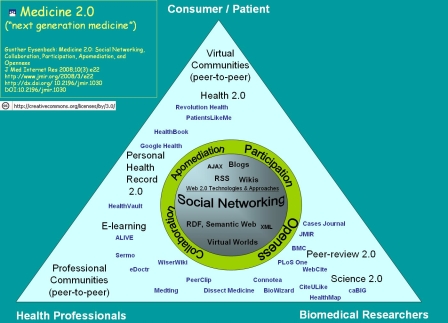
Medicine 2.0 Map (with some current exemplary applications and services)

While it may be too early to come up with an absolute definition of Medicine 2.0 or Health 2.0, Figure 1 shows a suggested framework, created in the context of a call for papers for the purpose of scoping the Medicine 2.0 congress and this theme issue [5]. The program of the first Medicine 2.0 conference [6] also gives a good idea of what academics feel is relevant to the field. An explanation of why we chose the title “Medicine 2.0” over “Health 2.0” has been given elsewhere [4]; it suffices to say at this point that most authors do not necessarily see a significant difference between Health 2.0 and Medicine 2.0 [7]—if anything, Medicine 2.0 is the broader concept and umbrella term which includes consumer-directed “medicine” or Health 2.0.

According to the model depicted in Figure 1, five major aspects (ideas, themes) emerge from Web 2.0 in health, health care, medicine, and science, which will outlive the specific tools and services offered. These emerging and recurring themes are (as displayed in the center of Figure 1):

1) Social Networking,

2) Participation,

3) Apomediation,

4) Collaboration, and

5) Openness.

While “Web 2.0”, “Medicine 2.0”, and “Health 2.0” are terms that should probably be avoided in academic discourse, any discussion and evaluations concerning the impact and effectiveness of Web 2.0 technologies should be framed around these themes. Each of the 5 themes will be considered in detail below.

Figure 1 also depicts the three main user groups of current Medicine 2.0 applications as a triangle: consumers/patients, health professionals, and biomedical researchers. While each of these user groups have received a different level of “formal” training, even end users (consumer, patients) can be seen as experts and—according to the Web 2.0 philosophy—their collective wisdom can and should be harnessed: “the health professional is an expert in identifying disease, while the patient is an expert in experiencing it” [8].

Current Medicine 2.0 applications can be situated somewhere in this triangle space, usually at one of the corners of the triangle, depending on which user group they are primarily targeting. However, the ideal Medicine 2.0 application would actually try to connect different user groups and foster collaboration between different user groups (for example, engaging the public in the biomedical research process), and thus move more towards the center of the triangle.

Putting it all together, the original definition of Medicine 2.0—as originally proposed in the context of soliciting submissions for the theme issue and the conference—was as follows [5]:

Medicine 2.0 applications, services and tools are Web-based services for health care consumers, caregivers, patients, health professionals, and biomedical researchers, that use Web 2.0 technologies and/or semantic web and virtual-reality tools, to enable and facilitate specifically social networking, participation, apomediation, collaboration, and openness within and between these user groups.

Interestingly, Benjamin Hughes' extensive literature review published in this issue concludes with a very similar definition [7].

There is however also a broader idea behind Medicine 2.0 or “second generation medicine”: the notion that healthcare systems need to move away from hospital-based medicine, focus on promoting health, provide healthcare in people's own homes, and empower consumers to take responsibility for their own health—much in line with what others and I have previously written about the field of consumer health informatics [9] (of which many Medicine 2.0 applications are prime examples). Thus, in this broader sense, Medicine 2.0 also stands for a new, better health system, which emphasizes collaboration, participation, apomediation, and openness, as opposed to the traditional, hierarchical, closed structures within health care and medicine.

## Social Networking

Social networking is central to many Web 2.0 and Medicine 2.0 applications and involves the explicit modeling of connections between people, forming a complex network of relations, which in turn enables and facilitates collaboration and collaborative filtering processes. For example, it enables users to see what their peers or others with a predefined relationship (“friends”, “colleagues”, “fellow patients” etc.) are doing; enables automated selection of “relevant” information (based on what peers are doing and reading on the Web); enables reputation and trust management, accountability and quality control, and fosters viral dissemination of information and applications (it is this “viral marketing” aspect that makes Web 2.0 applications so attractive to venture capitalists and public health practitioners alike). Moreover, social networking is a potentially powerful tool to engage users, in that it provides “social” incentives to enter, update, and manage personal information. Teenagers spend hours keeping their Facebook profile current, constantly updating their status. Now imagine the same generation of users turning their attention and energy to similar tools for health (what I called a “Healthbook” application). Will social networking be the killer application that gets people interested in personal health records, motivates users to take responsibility for their health and health information, and—more importantly—retain their interest over time? Will these mechanisms help to combat the “Law of Attrition” [10], ie, the phenomenon that many patients lose interest and stop using online health applications after some time?

**Figure 2:**
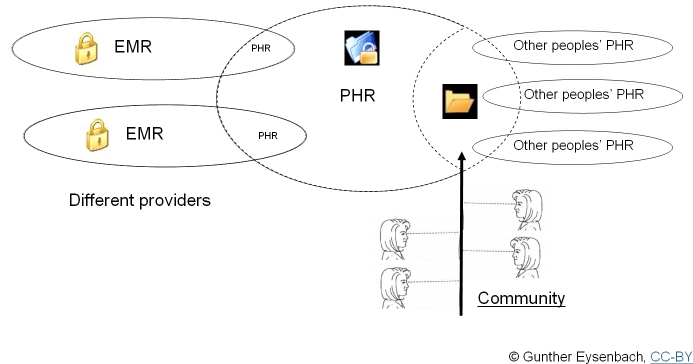
PHR 2.0: Conceptual model of a second generation of personal health records, which not only allows patients to access their electronic health record, but to share parts of it with other people, building communities around certain health topics and issues.

I predict that this will be a very active and interesting area of research. The social networking idea, which involves modeling relationships between actors, is a relatively new idea in health informatics. For example, what is traditionally “modeled” in electronic health records is usually medical information (symptoms, diagnosis, therapy), but not relationships between people. True, in most electronic health records we usually have some database fields for storing the name of the family physician, the attending physician, closest relatives and emergency contacts, and perhaps a narrative free text social anamnesis, but none of the existing health record systems support the explicit modeling of the patients’ or health professionals’ complex social network. When we combine social networking approaches with emerging technologies such as Personal Health Records, a new class of applications emerges—PHR 2.0 [3] (Figure 2).

For quality management and collaborative filtering, the application of social networking (and the attempt to model relationships) is not an entirely new idea. In fact, almost a decade ago, within the framework of the MedCertain and MedCircle projects, we started thinking about this and envisioned the explicit modeling of social relationships and information concerning “who said what about a specific website” as one promising way to guide consumers to high-quality information. We developed a vocabulary to describe relationships between those involved in quality initiatives on the Web, with the eventual goal being to build intelligent tools that can harness this information [11]. Today, this approach might be called a Web 3.0 application (which is a bit misleading, as the relevant technologies such as semantic web, RDF [resource description framework], FOAF [friend-of-a-friend] etc. pre-date most Web 2.0 technologies). Today we would probably rely on a folksonomy, rather than trying to develop a taxonomy.

## Participation

Participation is another central theme and core value in Medicine 2.0. This aspect is particularly important for consumers and patients but also extends to health professionals and researchers. Personal Health Records and, in particular, PHR 2.0 [3] are a part of this development. Over the past decade we have come a long way toward this goal of consumer participation in health care. When I first wrote about the promise of consumer health informatics opening up the possibility for consumers to access their electronic health record [9], this way of thinking was far from mainstream, and not many people thought this was a realistic or even desirable goal. But the Web and related technologies have changed attitudes and the culture in health care. The Internet has been a tool for users and citizens to get more involved and empowered, and Web 2.0 tools take this to a new level, as the philosophy of end-user participation and engagement (“trust your users”) is deeply ingrained in Web 2.0 thinking, exemplified by tools like wikis.

Wikis are the perfect example to illustrate that the “participation” theme is also relevant for other user groups, such as scientists or health professionals, and can be adopted for tasks like scholarly communication.

There is another aspect of Web 2.0 and Personal Health Records/Personal Health Application Platforms which excites consumers and researchers alike: These platforms provide—at least theoretically—unique opportunities to address directly the concerns of patients regarding secondary use of their data for research, and to facilitate obtaining informed consent for participation and data use in research studies in an ethical manner. For example, most patients do not want “the obtaining of consent [to participate in a research study] to detract from the reason for their appointment. They expected their health, not research, to be the focus of the consultation” [12]. PCHR platforms allow consumers to access and control their personal health information and provide the possibility to obtain consent in a different setting than during a clinical consultation: through the Internet, where it is contextualized by educational information. It can even be argued that *patient-access to their own data is a prerequisite for engaging the public*. As Mandl and colleagues argued: “Patients should be able to grant or deny study access to selected personal medical data. […] All these patient functions should be accessible from any web browser in the world.” [13]

In summary, the emergence of social networking platforms and applications such as Facebook or PatientsLikeMe [14], potentially combined with “PHR 2.0”—personal health records which allow users to share parts of their electronic health record with other users—create new levels of patient participation, as well as unique and unprecedented opportunities for engaging patients in their health, health care, and health research, and for connecting patients with informal and formal caregivers, health professionals, and researchers. However, it also creates complex privacy issues. For example, consumers—perceiving information they post or disclose on the Internet as ephemeral—may be unaware of the fact that web-information is often permanently archived and may be accessible long-term (eg, by future employers). Little is known about the actual consumer awareness of these privacy and “persistence” issues, in particular when it comes to young participating users [15].

## Apomediation

Apomediation is a new socio-technological term that was coined to avoid the term “Web 2.0” in the scholarly debate [16,17]. It characterizes the “third way” for users to identify trustworthy and credible information and services. The first possible approach is to use intermediaries (ie, middlemen or “gatekeepers”), for example health professionals giving “relevant” information to a patient. Trusted Web portals containing only information vetted by experts can also be seen as an intermediary. The second possibility is to bypass “middlemen” completely, which is commonly referred to as disintermediation. Examples are patients searching for information on the web, or travelers booking their flights directly on the booking system of an airline, bypassing travel agents. The third way, prevalent in the age of Web 2.0, is a special form of disintermediation: an information seeking strategy where people rely less on traditional experts and authorities as gatekeepers, but instead receive “guidance” from apomediaries, ie, networked collaborative filtering processes [16,17]. The difference between an intermediary and an apomediary is that an intermediary stands “in between” (latin: inter- means “in between”) the consumer and information, meaning that he is a necessary mediating agent to receive the information in the first place. As a result, the credibility and quality of the intermediary heavily determines the credibility and quality of the information a consumer receives. In contrast, apomediation means that there are agents (people, tools) which “stand by” (latin: apo- means separate, detached, away from) to guide a consumer to high quality information and services without being a prerequisite to obtain that information or service in the first place, and with limited individual power to alter or select the information that is being brokered. While these distinctions are not absolute (in practice, there may be a mix of both and people move back and forth between apomediation and intermediation models), it has been hypothesized that they influence how people judge credibility, as elaborated in more detail elsewhere [16].

**Figure 3:**
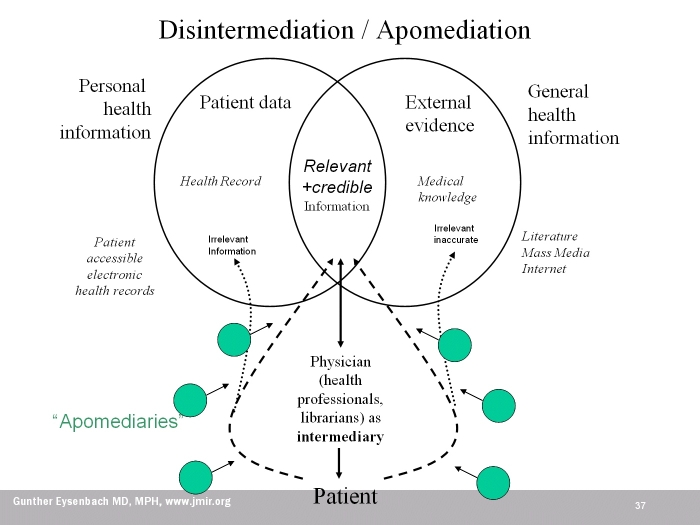
Apomediation in the health care field from the perspective of a patient.

In the health context, disintermediation (cutting out the middleman) means more direct access of consumers to their personal data (eg, in web accessible electronic health records—upper left circle of Figure 3) and general medical information (on the web—upper right circle of Figure 3). The traditional role of the middleman is to guide consumers to relevant and credible information (the intersection of both circles in the center of the diagram). Thus, the main problem of bypassing the middleman is that consumers may “get lost” in the vast amount of information and arrive at the wrong or irrelevant information (dotted arrows). Apomediation theory conceptualizes that “apomediaries” (which includes Web 2.0 approaches) can partly take over the role of the intermediary and “push” or “guide” users to relevant and accurate information (dashed arrows).

The Web 2.0 environment is essentially an “apomediated environment”, meaning that all the issues related to the apomediation model, summarized in Table 1 [16], are relevant for Web 2.0 and Medicine 2.0.

**Table 1:** Issues in an apomediation vs intermediation environment [16].

Dimension	Intermediation Environment	Disintermediation/Apomediation Environment
**Overarching Issues**		
Environment	Managed	Autonomous
Power	Centralized; power held by intermediaries (experts, authorities)	Decentralized; empowerment of information seekers
Dependence	Information seekers dependent on intermediaries (physicians, parents); intermediaries are *necessary*	Information seekers are emancipated from intermediaries as apomediaries (peers, technology) provide *guidance;* apomediaries are *optional*
Nature of Information Consumption	Consumers tend to be passive receivers of information	Consumers are “prosumers” (ie,, co-producers of information)
Nature of Interaction	Traditional 1:1 interaction between intermediary and information seeker	Complex individual- and group-based interactions in a networked environment
Information Filtering	“Upstream” filtering with top-down quality assurance mechanisms	“Downstream filtering” with bottom-up quality assurance mechanisms
Learning	More formal; learning through consumption of information	More informal; learning through participation, application, and information production
Cognitive Elaboration	Lower cognitive elaboration required by information receivers	Higher elaboration required by information seekers; higher cognitive load unless assistance through intelligent tools
User	More suitable for and/or desired by preadolescents, inexperienced or less information literate consumers, or patients with acute illness	More suitable for and/or desired by older adolescents and adults, experienced or information literate consumers, or patients with chronic conditions
**Credibility Issues**		
Expertise	Based on traditional credentials (eg, seniority, professional degrees)	Based on first-hand experience or that of peers
Bias	May promote facts over opinion, but opportunity for intermediary to introduce biases	May bestow more credibility to opinions rather than facts
Source Credibility	Based on the believability of the source’s authority; source credibility is more important than message credibility	Based on believability of apomediaries; message credibility and credibility of apomediaries are more important than source credibility
Message Credibility	Based on professional and precise language, comprehensiveness, use of citations, etc.	Based on understandable language, knowing or having experienced issues personally
Credibility Hubs	Static (experts)	Dynamic (opinion leaders)
Credibility Evaluations	Binary	Spectral

Apomediation theory argues that apomediaries, such as users and friends in the case of Digg, can help users navigate through the onslaught of information afforded by networked digital media, providing additional credibility cues and supplying further metainformation. Other examples of apomediaries and apomediation tools include consumer ratings on amazon.com or epinions.com; technologies like PICS or MedPICS labels and its RDF successors that enable machine-processable dissemination and interpretation of user ratings [18]; collaborative filtering and recommender systems as exemplified by StumbleUpon.com; and other second generation Internet-based services and tools that let people collaborate on a massive scale and share information online in new ways, including social networking sites, social bookmarking, blogs, wikis, communication tools, and folksonomies.

**Figure 4:**
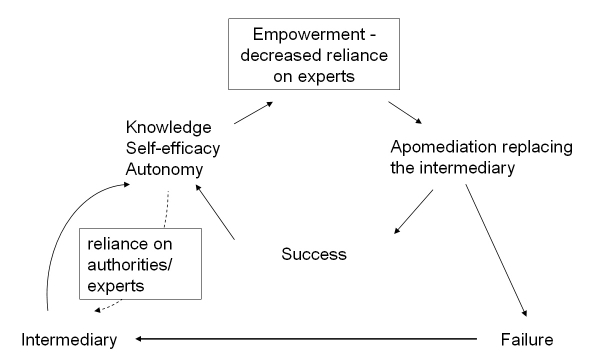
Dynamic Intermediation-Disintermiation-Apomediation model (DIDA) [16].

The Dynamic Intermediation-Disintermiation-Apomediation model (DIDA) (illustrated in Figure 4) argues that whether or not consumers prefer an apomediation or intermediation environment is highly situation-specific, and key variables in determining consumer preference for apomediation are autonomy, self-efficacy, and knowledge in a specific area for which information or support is sought. For example, a cancer patient may initially prefer an intermediary to satisfy his information needs, but with growing autonomy, self-efficacy, and knowledge, the same patient may later prefer Web 2.0 approaches to guide him to information deemed trustworthy.

Apomediation is not only important to the consumer as end user and the health professional as former intermediary. Both health professionals and scientists themselves are also switching from intermediaries to apomediaries. For example, two decades ago, researchers and health professionals still used intermediaries such as information brokers to conduct a Medline search for them, but then disintermediation took over, and they were able to search in PubMed directly. Today, these tools are complemented by “apomediaries”, for example shared bookmarking tools such as CiteULike, Connotea, or WebCite, where people receive pointers to recently published relevant literature based on what others with a similar profile and interests have cited or bookmarked.

In science, we are also witnessing an apomediation process (sometimes called “Science 2.0”), with changing roles for the former intermediaries such as journals and professional publishers. Much of the communication between scientists now takes place on the Web before an article is actually published [19]. This onslaught of information necessitates the use of “apomediaries” (such as shared bookmarks) to guide users to relevant information on the Web. One can also predict that journals themselves will experiment increasingly with peer-review models that rely more on networked, bottom-up review processes, as opposed to relying on traditional “expert” peer-reviewers. Such models are not without challenges and require a cultural shift as well as strong incentives. Nature’s recent open peer-review experiment suggests that most researchers “are too busy, and lack sufficient career incentive, to venture onto a venue such as *Nature*'s website and post public, critical assessments of their peers' work” [20]).

These apparent failures highlight the problem that—as has been previously pointed out [16]—what works for the entertainment industry, namely rating tools for users to rate movies, music, etc., may not necessarily work in the medical or scientific field. Productivity tools (including health applications) have to pass a different hurdle than “fun” applications such as Facebook – they have to be trustworthy, secure and people have to see an (immediate) benefit. There is the question of incentives for users to participate and to contribute constructively to a virtual community. Social networking sites such as Facebook or Myspace work because for young people it is important to be visible, and there is a considerable social and peer pressure for youth to have a presence and a positive “karma” or reputation on such sites, so much so that there is a grey market for users to “buy” virtual friends [21]. This of course highlights another problem – which is that even networked “apomediation” models are liable to fraud and “Scam 2.0”. It is an open research question whether, and under which circumstances, apomediation models work better than intermediation approaches, and how apomediation models can be made less susceptible to fraud.

## Collaboration

Collaboration specifically means to connect groups of people with each other who have not, or have insufficiently, interacted with each other. In the “researcher” corner of the Medicine 2.0 triangle, this may refer to bringing together scientists using tools and approaches such as the ones described by Schleyer [22] or Falkman [23] in this theme issue. But it also involves encouraging collaboration between diverse user groups, including for example fostering public participation and engagement in research issues, and user engagement in health care decisions. Collaboration between researchers on one hand, and the public or health professionals on the other hand, also means improved possibilities for knowledge translation and getting research findings into practice.

## Openness

Finally, I would argue that openness is another important and emerging theme to consider in the Web 2.0 context. On one level—the technical level—Web 2.0 stands for transparency, interoperability, open source, and open interfaces: “Don’t lock me in”, “my data belongs to me”, “web as operating system”, and “open up your API” are popular philosophies associated with Web 2.0. Personal Health Application platforms such as HealthVault and Google Health both have APIs for other applications to connect to.

What is perhaps most significant about this development is that the “openness” philosophy of Web 2.0 tools will also raise the expectations of the Facebook generation in terms of dealing with their health data. Web 2.0 savvy consumers will push the envelope and demand more than just an institutions-specific “portal” (also called “tethered PHR”) which allows them to view or access their data but not to do anything else with it. Patients 2.0 will demand full control over their data (as a minimum, XML export!). Many current Medicine 2.0 applications fall short in that regard, in that people can feed information into the system but can’t get it out again.

On another—societal—level, Medicine 2.0 also implies openness and transparency which enables access to other kinds of information and data the public has historically had limited access to, for example research and research data (open access journals, open data etc.), and which even allows the public to engage in the research process itself (open peer-review).

## Conclusion

Openness being a key theme in Web 2.0, it is very appropriate that the *Journal of Medical Internet Research*—an open access journal—sponsors the first conference and publishes the first theme issue on Medicine 2.0. Regardless of what labels we attach to this emerging field, those interested in collaborative tools and empowerment of end users will find stimulating new perspectives for research and policy in both the conference and this theme issue. We also do not see this as a one-time event, as JMIR will continue to consider and publish submissions which fall into this area, and the Medicine 2.0 Congress is likely to be an annual event focusing on the latest technologies and societal developments to support the five themes. In analogy to what Tim Berners-Lee once said about Web 2.0—that it was “what the Web was supposed to be all along” [24])—we could also say that “Medicine 2.0 is what ehealth was supposed to be all along”, and fostering and encouraging these developments was why this journal was created in the first place.

## Figures and Tables

**Figure 5:**
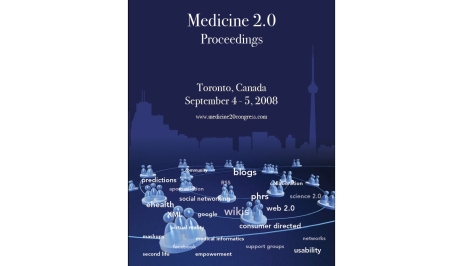
Medicine 2.0 Proceedings: Cover page

## Multimedia Appendix

Eysenbach G (ed.). Medicine 2.0 Proceedings. Toronto, ON, Canada, Sept 4-5, 2008 [Full text PDF]

## Abbreviations

API: application programming interface
PCHR: Personally Controlled Health Record
PHA: Personal Health Application
PHR: Personal Health Record
