# Supplementary material for: Medicine 2.0: Social Networking, Collaboration, Participation, Apomediation, and Openness
Source: J Med Internet Res. 2008 Aug 25;10(3):e22. doi: 10.2196/jmir.1030 (PMC2626430; doi:10.2196/jmir.1030)
Supplement: Supplementary file 1 [file jmir_v10i3e22_app1.pdf]

Medicine 2.0
Proceedings

Toronto, Canada
September 4 - 5, 2008

www.medicine20congress.com

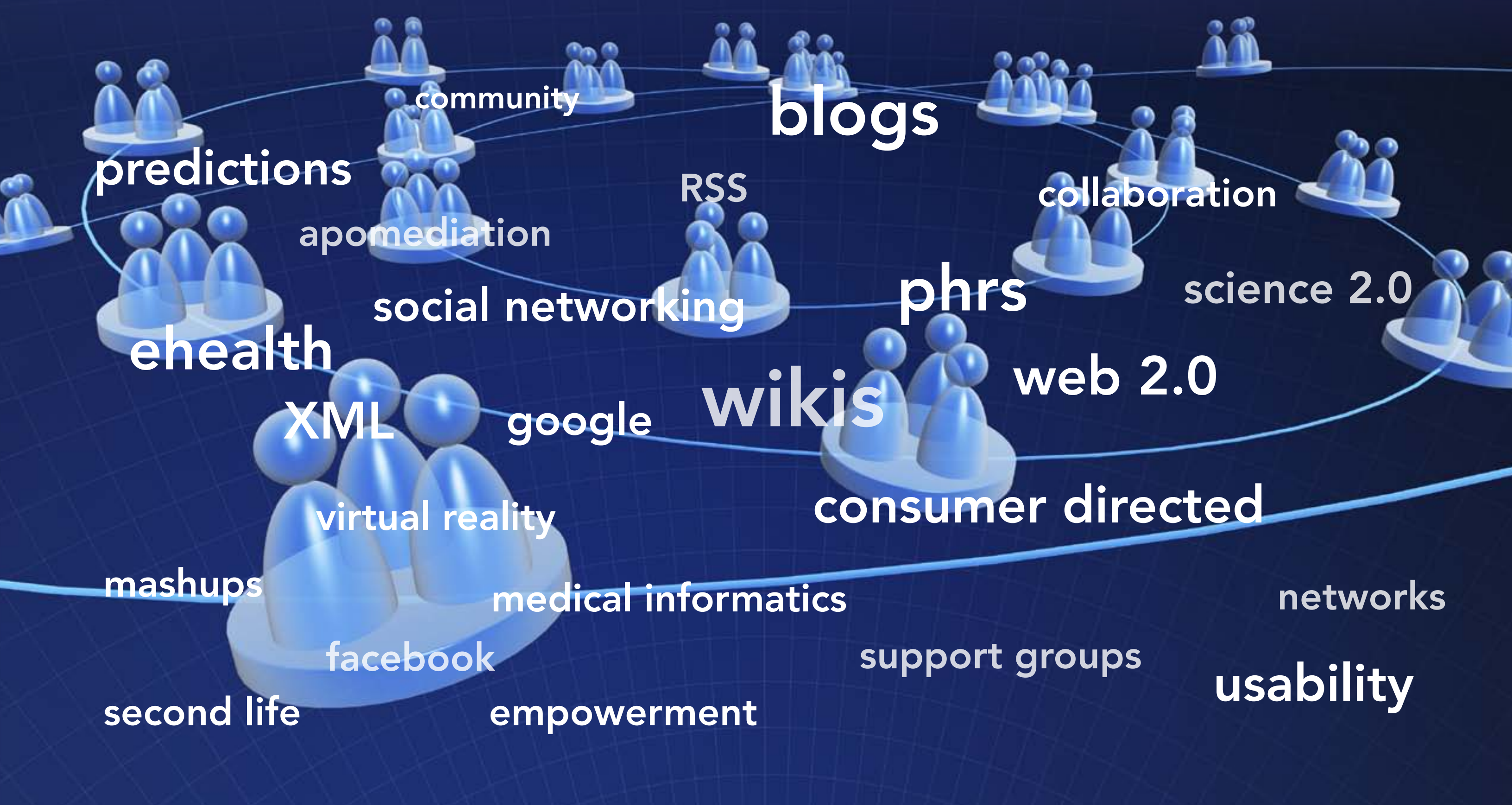

Eysenbach G. (ed.): Medicine 2.0 Proceedings. Toronto, ON, Canada, Sept 4-5, 2008 http://www.medicine20congress.com.

Published as Multimedia Appendix in: Eysenbach G. Medicine 2.0: Social Networking, Collaboration, Participation, Apomediation, and Openness. J Med Internet Res 2008;10(3):e22. http://www.jmir.org/2008/3/e22/ doi:10.2196/jmir.1030

NOTE: Presentation times and room assignments provided in this booklet are subject to change. In case of information conflicting with the Medicine 2.0 Final Program, the program information prevails

Editorial

Medicine 2.0: Social Networking, Collaboration, Participation, Apomediation, and Openness

Gunther Eysenbach, MD, MPH

Corresponding Author:
Gunther Eysenbach, MD, MPH
Centre for Global eHealth Innovation
University of Toronto and University Health Network
190 Elizabeth Street
Toronto ON M5G 2C4
Canada
Phone: +1 416 340 4800 ext 6427
Fax: +1 416 340 3595
Email: geysenba@uhnres.utoronto.ca

Abstract

In a very significant development for eHealth, a broad adoption of Web 2.0 technologies and approaches coincides with the more recent emergence of Personal Health Application Platforms and Personally Controlled Health Records such as Google Health, Microsoft HealthVault, and Dossia. "Medicine 2.0" applications, services, and tools are defined as Web-based services for health care consumers, caregivers, patients, health professionals, and biomedical researchers, that use Web 2.0 technologies and/or semantic web and virtual reality approaches to enable and facilitate specifically 1) social networking, 2) participation, 3) apomediation, 4) openness, and 5) collaboration, within and between these user groups. The Journal of Medical Internet Research (JMIR) publishes a Medicine 2.0 theme issue and sponsors a conference on "How Social Networking and Web 2.0 changes Health, Health Care, Medicine, and Biomedical Research", to stimulate and encourage research in these five areas.

(J Med Internet Res 2008;10(3):e22) doi:10.2196/jmir.1030

KEYWORDS

Cooperative Behavior; Education; Information Storage and Retrieval; Interpersonal Relations; Organizational Innovation; Social Behavior; User-Computer Interface; Online Systems; Patient Education as Topic; Terminology as Topic; Medical Record Linkage; Self Care; Internet; Health Communication; Collaboration; Research

JMIR's Theme Issue and Conference on Medicine 2.0

In the past 9 years, the *Journal of Medical Internet Research* (JMIR) has been publishing hundreds of research and opinion articles on how the Internet is changing medical practice, transforming biomedical research, and empowering health care consumers. While we have seen many new concepts and terms appear and disappear, the term "Web 2.0" (and its derivatives, for example "Web 3.0") is increasingly entering our discussions and is likely here to stay.

It is easy to dismiss some of the "hype" around Web 2.0 as a marketing gimmick or rhetoric geared towards attracting venture capital for Web 2.0 startups. However, most Internet researchers and developers probably also agree that recent advances in web technologies and user interfaces have greatly changed the design, appearance, stickiness, and pervasiveness of Web applications, and in many cases transformed the way users interact with them. Perhaps equally importantly, it also has changed the *expectations* of users. After some hard lessons learned from failed Web ventures which disappeared overnight taking any user-generated data with them, people expect Web applications to be open and interoperable. Improved communication between separate software applications ("mashups") via open Web standards leads to improved collaboration and communication across applications. Social networking approaches revolutionize the way people collaborate, identify potential collaborators or friends, communicate with each other, and identify information that is relevant for them. And finally, Web 2.0 technologies such as AJAX lead to improved Web interfaces that mimic the real-time responsiveness of desktop applications within a browser window. Semantic Web applications (sometimes called Web 3.0) and 3D environments (such as Second Life) can also be seen as second generation Web technologies.

The emergence and broad adoption of Web 2.0 technologies and approaches coincides with the more recent emergence of Personal Health Application (PHA) Platforms (also called Personally Controlled Health Record [PCHR] platforms or "health record banks") such as Google Health, Microsoft HealthVault, and Dossia, where data is—at the request of the consumer—pulled from various sources (including electronic

XSL•FO
RenderX

health records). As eloquently argued by Mandl and colleagues in the *New England Journal of Medicine*, these developments represent “tectonic shifts in the health information economy” [1] with far-reaching consequences for patient involvement, as the gravity shifts away from health care providers as the sole custodian of medical data. PHA (or PCHR) platforms, “where health care consumers independently decide about subsequent disclosure [of health data]” represent nothing short of a “disruptive innovation that inverts the current approach to medical records in that they are created by and reside with patients who grant permission for their use to institutions, clinicians, researchers, public health agencies, and other users of medical information” [1]. A randomized controlled trial with the PCHR system Dossia illustrates the potential of PCHR for public health [2].

It easy to imagine that the combination of both trends—Personal Health Records combined with social networking, what I have called “PHR 2.0” [3]—may lead to a powerful new generation of health applications, where people share parts of their electronic health records with other consumers and “crowdsource” the collective wisdom of other patients and professionals. Advances in genetic medicine will further personalize and tailor health information, based on data stored in personal health records.

Finally, we are seeing developments in biomedical research (“Science 2.0”) and scholarly publishing which apply the same principles of participation and collaboration across different points along the continuum of knowledge production and dissemination.

In an attempt to foster and stimulate research in these areas, JMIR is proud to sponsor the new Medicine 2.0 congress series [4,5] and to publish this theme issue on “How Social Networking and Web 2.0 changes Health, Health Care, Medicine and Biomedical Research”.

On the Scope and Definition of Medicine 2.0

While it may be too early to come up with an absolute definition of Medicine 2.0 or Health 2.0, Figure 1 shows a suggested framework, created in the context of a call for papers for the purpose of scoping the Medicine 2.0 congress and this theme issue [5]. The program of the first Medicine 2.0 conference [6] also gives a good idea of what academics feel is relevant to the field. An explanation of why we chose the title “Medicine 2.0” over “Health 2.0” has been given elsewhere [4]; it suffices to say at this point that most authors do not necessarily see a significant difference between Health 2.0 and Medicine 2.0 [7]—if anything, Medicine 2.0 is the broader concept and umbrella term which includes consumer-directed “medicine” or Health 2.0.

According to the model depicted in Figure 1, five major aspects (ideas, themes) emerge from Web 2.0 in health, health care, medicine, and science, which will outlive the specific tools and services offered. These emerging and recurring themes are (as displayed in the center of Figure 1):

1) Social Networking,

2) Participation,

3) Apomediation,

4) Collaboration, and

5) Openness.

Figure 1. Medicine 2.0 Map (with some current exemplary applications and services)

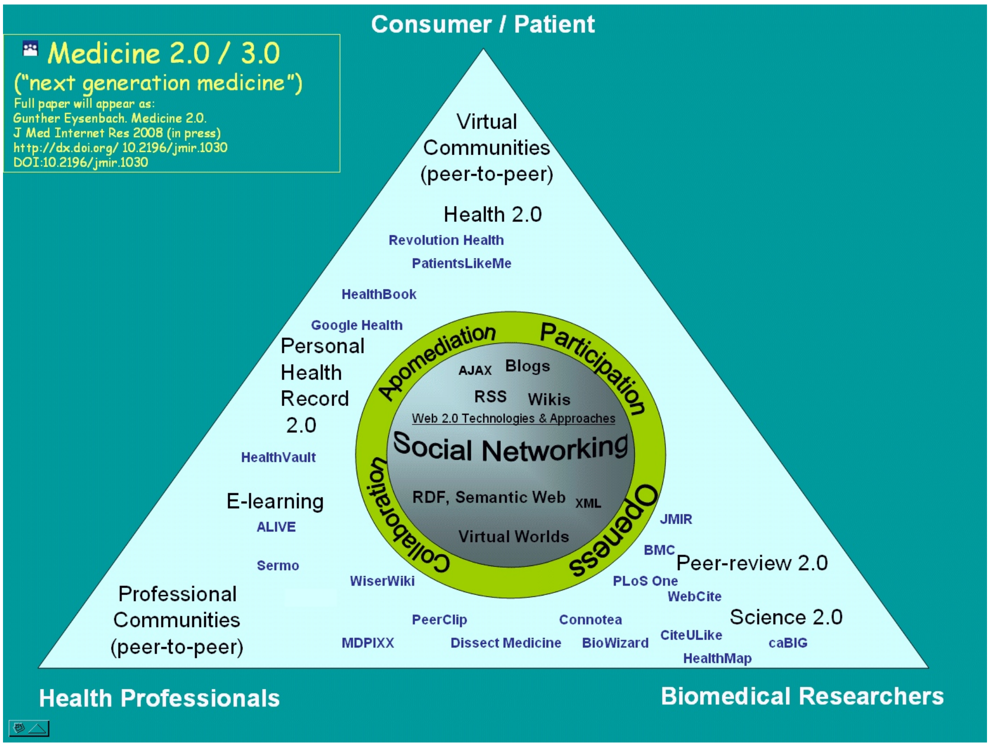

XSL•FO
RenderX

While “Web 2.0”, “Medicine 2.0”, and “Health 2.0” are terms that should probably be avoided in academic discourse, any discussion and evaluations concerning the impact and effectiveness of Web 2.0 technologies should be framed around these themes. Each of the 5 themes will be considered in detail below.

Figure 1 also depicts the three main user groups of current Medicine 2.0 applications as a triangle: consumers/patients, health professionals, and biomedical researchers. While each of these user groups have received a different level of “formal” training, even end users (consumer, patients) can be seen as experts and—according to the Web 2.0 philosophy—their collective wisdom can and should be harnessed: “the health professional is an expert in identifying disease, while the patient is an expert in experiencing it” [8].

Current Medicine 2.0 applications can be situated somewhere in this triangle space, usually at one of the corners of the triangle, depending on which user group they are primarily targeting. However, the ideal Medicine 2.0 application would actually try to connect different user groups and foster collaboration between different user groups (for example, engaging the public in the biomedical research process), and thus move more towards the center of the triangle.

Putting it all together, the original definition of Medicine 2.0—as originally proposed in the context of soliciting submissions for the theme issue and the conference—was as follows [5]:

> *Medicine 2.0 applications, services and tools are Web-based services for health care consumers, caregivers, patients, health professionals, and biomedical researchers, that use Web 2.0 technologies and/or semantic web and virtual-reality tools, to enable and facilitate specifically social networking, participation, apomediation, collaboration, and openness within and between these user groups.*

Interestingly, Benjamin Hughes' extensive literature review published in this issue concludes with a very similar definition [7].

There is however also a broader idea behind Medicine 2.0 or “second generation medicine”: the notion that healthcare systems need to move away from hospital-based medicine, focus on promoting health, provide healthcare in people's own homes, and empower consumers to take responsibility for their own health—much in line with what others and I have previously written about the field of consumer health informatics [9] (of which many Medicine 2.0 applications are prime examples). Thus, in this broader sense, Medicine 2.0 also stands for a new, better health system, which emphasizes collaboration, participation, apomediation, and openness, as opposed to the traditional, hierarchical, closed structures within health care and medicine.

Social Networking

Social networking is central to many Web 2.0 and Medicine 2.0 applications and involves the explicit modeling of connections between people, forming a complex network of relations, which in turn enables and facilitates collaboration and collaborative filtering processes. For example, it enables users to see what their peers or others with a predefined relationship (“friends”, “colleagues”, “fellow patients” etc.) are doing; enables automated selection of “relevant” information (based on what peers are doing and reading on the Web); enables reputation and trust management, accountability and quality control, and fosters viral dissemination of information and applications (it is this “viral marketing” aspect that makes Web 2.0 applications so attractive to venture capitalists and public health practitioners alike). Moreover, social networking is a potentially powerful tool to engage users, in that it provides “social” incentives to enter, update, and manage personal information. Teenagers spend hours keeping their Facebook profile current, constantly updating their status. Now imagine the same generation of users turning their attention and energy to similar tools for health (what I called a “Healthbook” application). Will social networking be the killer application that gets people interested in personal health records, motivates users to take responsibility for their health and health information, and—more importantly—retain their interest over time? Will these mechanisms help to combat the “Law of Attrition” [10], ie, the phenomenon that many patients lose interest and stop using online health applications after some time?

I predict that this will be a very active and interesting area of research. The social networking idea, which involves modeling relationships between actors, is a relatively new idea in health informatics. For example, what is traditionally “modeled” in electronic health records is usually medical information (symptoms, diagnosis, therapy), but not relationships between people. True, in most electronic health records we usually have some database fields for storing the name of the family physician, the attending physician, closest relatives and emergency contacts, and perhaps a narrative free text social anamnesis, but none of the existing health record systems support the explicit modeling of the patients’ or health professionals’ complex social network. When we combine social networking approaches with emerging technologies such as Personal Health Records, a new class of applications emerges—PHR 2.0 [3] (Figure 2).

XSL•FO
RenderX

Figure 2. PHR 2.0: Conceptual model of a second generation of personal health records, which not only allows patients to access their electronic health record, but to share parts of it with other people, building communities around certain health topics and issues.

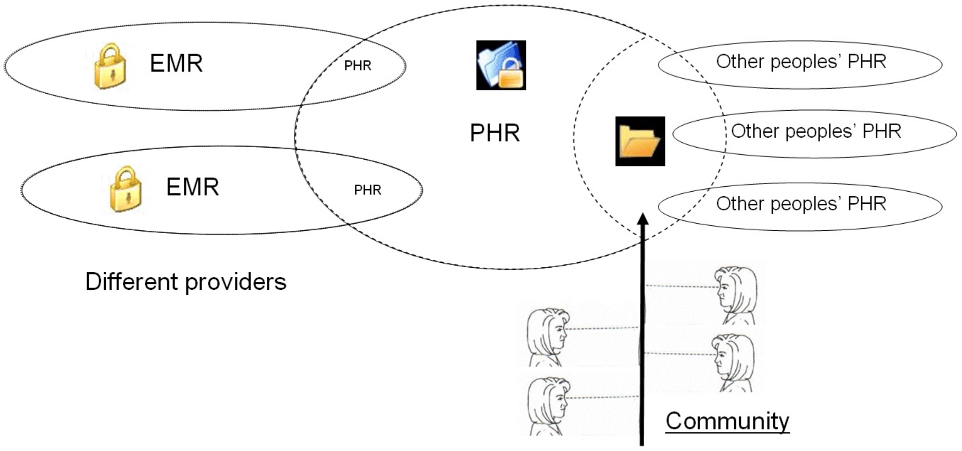

© Gunther Eysenbach, CC-BY

For quality management and collaborative filtering, the application of social networking (and the attempt to model relationships) is not an entirely new idea. In fact, almost a decade ago, within the framework of the MedCertain and MedCircle projects, we started thinking about this and envisioned the explicit modeling of social relationships and information concerning "who said what about a specific website" as one promising way to guide consumers to high-quality information. We developed a vocabulary to describe relationships between those involved in quality initiatives on the Web, with the eventual goal being to build intelligent tools that can harness this information [11]. Today, this approach might be called a Web 3.0 application (which is a bit misleading, as the relevant technologies such as semantic web, RDF [resource description framework], FOAF [friend-of-a-friend] etc. pre-date most Web 2.0 technologies). Today we would probably rely on a folksonomy, rather than trying to develop a taxonomy.

Participation

Participation is another central theme and core value in Medicine 2.0. This aspect is particularly important for consumers and patients but also extends to health professionals and researchers. Personal Health Records and, in particular, PHR 2.0 [3] are a part of this development. Over the past decade we have come a long way toward this goal of consumer participation in health care. When I first wrote about the promise of consumer health informatics opening up the possibility for consumers to access their electronic health record [9], this way of thinking was far from mainstream, and not many people thought this was a realistic or even desirable goal. But the Web and related technologies have changed attitudes and the culture in health care. The Internet has been a tool for users and citizens to get more involved and empowered, and Web 2.0 tools take this to a new level, as the philosophy of end-user participation and engagement ("trust your users") is deeply ingrained in Web 2.0 thinking, exemplified by tools like wikis.

Wikis are the perfect example to illustrate that the "participation" theme is also relevant for other user groups, such as scientists or health professionals, and can be adopted for tasks like scholarly communication.

There is another aspect of Web 2.0 and Personal Health Records/Personal Health Application Platforms which excites consumers and researchers alike: These platforms provide—at least theoretically—unique opportunities to address directly the concerns of patients regarding secondary use of their data for research, and to facilitate obtaining informed consent for participation and data use in research studies in an ethical manner. For example, most patients do not want "the obtaining of consent [to participate in a research study] to detract from the reason for their appointment. They expected their health, not research, to be the focus of the consultation" [12]. PCHR platforms allow consumers to access and control their personal health information and provide the possibility to obtain consent in a different setting than during a clinical consultation: through the Internet, where it is contextualized by educational information. It can even be argued that *patient-access to their own data is a prerequisite for engaging the public*. As Mandl and colleagues argued: "Patients should be able to grant or deny study access to selected personal medical data. […] All these patient functions should be accessible from any web browser in the world." [13]

In summary, the emergence of social networking platforms and applications such as Facebook or PatientsLikeMe [14], potentially combined with "PHR 2.0"—personal health records which allow users to share parts of their electronic health record with other users—create new levels of patient participation, as well as unique and unprecedented opportunities for engaging patients in their health, health care, and health research, and for connecting patients with informal and formal caregivers, health professionals, and researchers. However, it also creates complex privacy issues. For example, consumers—perceiving information they post or disclose on the Internet as ephemeral—may be unaware of the fact that web-information is often permanently archived and may be accessible long-term (eg, by future employers). Little is known about the actual consumer awareness of these privacy and "persistence" issues, in particular when it comes to young participating users [15].

XSL•FO
RenderX

Apomediation

Apomediation is a new socio-technological term that was coined to avoid the term “Web 2.0” in the scholarly debate [16,17]. It characterizes the “third way” for users to identify trustworthy and credible information and services. The first possible approach is to use intermediaries (ie, middlemen or “gatekeepers”), for example health professionals giving “relevant” information to a patient. Trusted Web portals containing only information vetted by experts can also be seen as an intermediary. The second possibility is to bypass “middlemen” completely, which is commonly referred to as disintermediation. Examples are patients searching for information on the web, or travelers booking their flights directly on the booking system of an airline, bypassing travel agents. The third way, prevalent in the age of Web 2.0, is a special form of disintermediation: an information seeking strategy where people rely less on traditional experts and authorities as gatekeepers, but instead receive “guidance” from apomediaries, ie, networked collaborative filtering processes [16,17]. The difference between an intermediary and an apomediary is that an intermediary stands “in between” (latin: inter- means “in between”) the consumer and information, meaning that he is a necessary mediating agent to receive the information in the first place. As a result, the credibility and quality of the intermediary heavily determines the credibility and quality of the information a consumer receives. In contrast, apomediation means that there are agents (people, tools) which “stand by” (latin: apo- means separate, detached, away from) to guide a consumer to high quality information and services without being a prerequisite to obtain that information or service in the first place, and with limited individual power to alter or select the information that is being brokered. While these distinctions are not absolute (in practice, there may be a mix of both and people move back and forth between apomediation and intermediation models), it has been hypothesized that they influence how people judge credibility, as elaborated in more detail elsewhere [16].

In the health context, disintermediation (cutting out the middleman) means more direct access of consumers to their personal data (eg, in web accessible electronic health records—upper left circle of Figure 3) and general medical information (on the web—upper right circle of Figure 3). The traditional role of the middleman is to guide consumers to relevant and credible information (the intersection of both circles in the center of the diagram). Thus, the main problem of bypassing the middleman is that consumers may “get lost” in the vast amount of information and arrive at the wrong or irrelevant information (dotted arrows). Apomediation theory conceptualizes that “apomediaries” (which includes Web 2.0 approaches) can partly take over the role of the intermediary and “push” or “guide” users to relevant and accurate information (dashed arrows).

The Web 2.0 environment is essentially an “apomediated environment”, meaning that all the issues related to the apomediation model, summarized in Table 1 [16], are relevant for Web 2.0 and Medicine 2.0.

Figure 3. Apomediation in the health care field from the perspective of a patient.

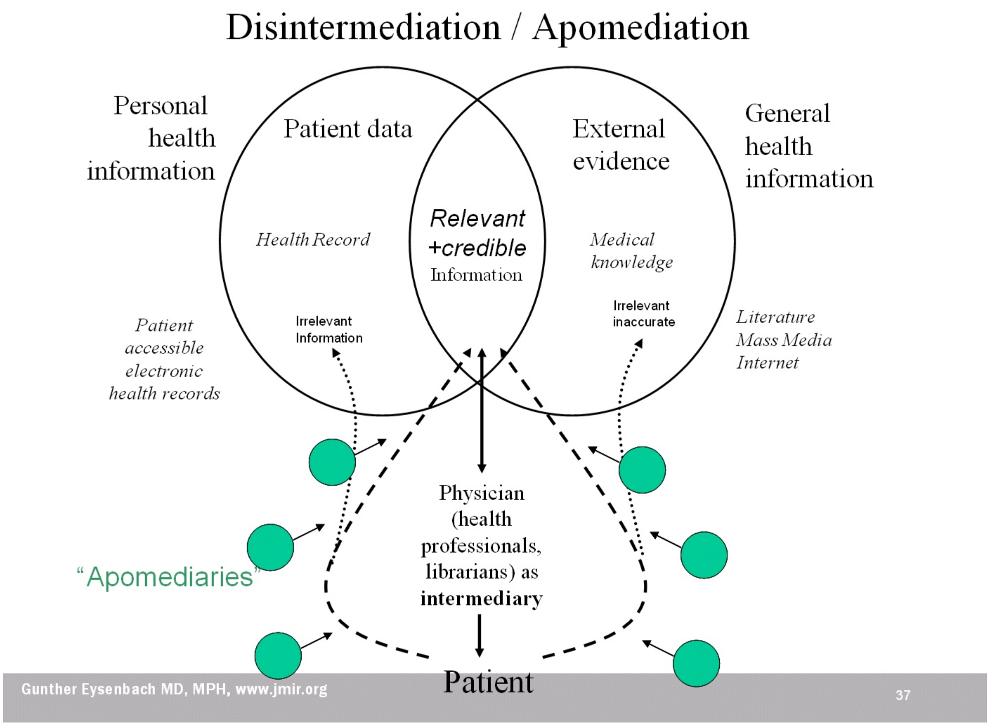

XSL•FO
RenderX

Table 1. Issues in an apomediation vs intermediation environment [16].

Dimension	Intermediation Environment	Disintermediation/Apomediation Environment
**Overarching Issues**		
Environment	Managed	Autonomous
Power	Centralized; power held by intermediaries (experts, authorities)	Decentralized; empowerment of information seekers
Dependence	Information seekers dependent on intermediaries (physicians, parents); intermediaries are *necessary*	Information seekers are emancipated from intermediaries as apomediaries (peers, technology) provide *guidance;* apomediaries are *optional*
Nature of Information Consumption	Consumers tend to be passive receivers of information	Consumers are “prosumers” (ie,, co-producers of information)
Nature of Interaction	Traditional 1:1 interaction between intermediary and information seeker	Complex individual- and group-based interactions in a networked environment
Information Filtering	“Upstream” filtering with top-down quality assurance mechanisms	“Downstream filtering” with bottom-up quality assurance mechanisms
Learning	More formal; learning through consumption of information	More informal; learning through participation, application, and information production
Cognitive Elaboration	Lower cognitive elaboration required by information receivers	Higher elaboration required by information seekers; higher cognitive load unless assistance through intelligent tools
User	More suitable for and/or desired by preadolescents, inexperienced or less information literate consumers, or patients with acute illness	More suitable for and/or desired by older adolescents and adults, experienced or information literate consumers, or patients with chronic conditions
**Credibility Issues**		
Expertise	Based on traditional credentials (eg, seniority, professional degrees)	Based on first-hand experience or that of peers
Bias	May promote facts over opinion, but opportunity for intermediary to introduce biases	May bestow more credibility to opinions rather than facts
Source Credibility	Based on the believability of the source’s authority; source credibility is more important than message credibility	Based on believability of apomediaries; message credibility and credibility of apomediaries are more important than source credibility
Message Credibility	Based on professional and precise language, comprehensiveness, use of citations, etc.	Based on understandable language, knowing or having experienced issues personally
Credibility Hubs	Static (experts)	Dynamic (opinion leaders)
Credibility Evaluations	Binary	Spectral

Apomediation theory argues that apomediaries, such as users and friends in the case of Digg, can help users navigate through the onslaught of information afforded by networked digital media, providing additional credibility cues and supplying further metainformation. Other examples of apomediaries and apomediation tools include consumer ratings on amazon.com or epinions.com; technologies like PICS or MedPICS labels and its RDF successors that enable machine-processable dissemination and interpretation of user ratings [18]; collaborative filtering and recommender systems as exemplified by StumbleUpon.com; and other second generation Internet-based services and tools that let people collaborate on a massive scale and share information online in new ways, including social networking sites, social bookmarking, blogs, wikis, communication tools, and folksonomies.

The Dynamic Intermediation-Disintermiation-Apomediation model (DIDA) (illustrated in Figure 4) argues that whether or not consumers prefer an apomediation or intermediation environment is highly situation-specific, and key variables in determining consumer preference for apomediation are autonomy, self-efficacy, and knowledge in a specific area for which information or support is sought. For example, a cancer patient may initially prefer an intermediary to satisfy his information needs, but with growing autonomy, self-efficacy, and knowledge, the same patient may later prefer Web 2.0 approaches to guide him to information deemed trustworthy.

XSL•FO
RenderX

Figure 4. Dynamic Intermediation-Disintermiation-Apomediation model (DIDA) [16].

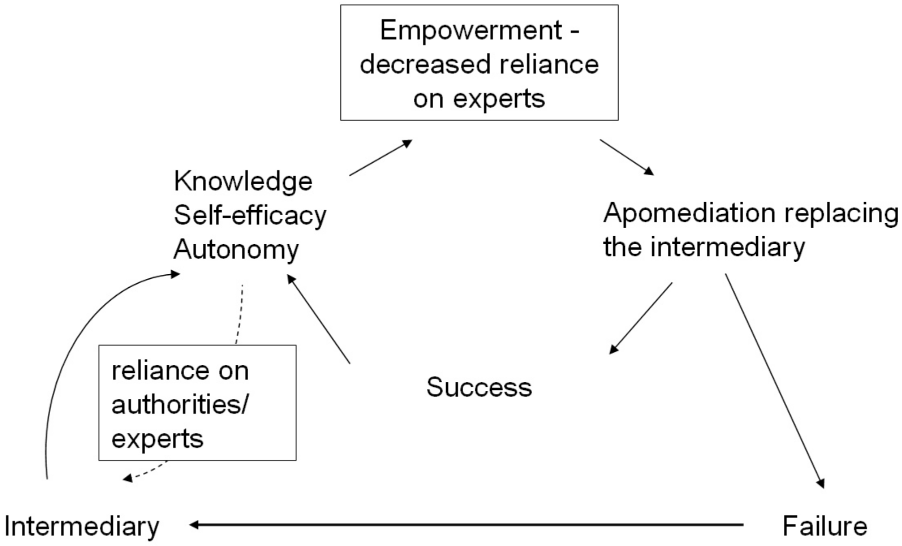

Apomediation is not only important to the consumer as end user and the health professional as former intermediary. Both health professionals and scientists themselves are also switching from intermediaries to apomediaries. For example, two decades ago, researchers and health professionals still used intermediaries such as information brokers to conduct a Medline search for them, but then disintermediation took over, and they were able to search in PubMed directly. Today, these tools are complemented by "apomediaries", for example shared bookmarking tools such as CiteULike, Connotea, or WebCite, where people receive pointers to recently published relevant literature based on what others with a similar profile and interests have cited or bookmarked.

In science, we are also witnessing an apomediation process (sometimes called "Science 2.0"), with changing roles for the former intermediaries such as journals and professional publishers. Much of the communication between scientists now takes place on the Web before an article is actually published [19]. This onslaught of information necessitates the use of "apomediaries" (such as shared bookmarks) to guide users to relevant information on the Web. One can also predict that journals themselves will experiment increasingly with peer-review models that rely more on networked, bottom-up review processes, as opposed to relying on traditional "expert" peer-reviewers. Such models are not without challenges and require a cultural shift as well as strong incentives. Nature's recent open peer-review experiment suggests that most researchers "are too busy, and lack sufficient career incentive, to venture onto a venue such as *Nature*'s website and post public, critical assessments of their peers' work" [20]).

These apparent failures highlight the problem that—as has been previously pointed out [16]—what works for the entertainment industry, namely rating tools for users to rate movies, music, etc., may not necessarily work in the medical or scientific field. Productivity tools (including health applications) have to pass a different hurdle than "fun" applications such as Facebook – they have to be trustworthy, secure and people have to see an (immediate) benefit. There is the question of incentives for users to participate and to contribute constructively to a virtual community. Social networking sites such as Facebook or Myspace work because for young people it is important to be visible, and there is a considerable social and peer pressure for youth to have a presence and a positive "karma" or reputation on such sites, so much so that there is a grey market for users to "buy" virtual friends [21]. This of course highlights another problem – which is that even networked "apomediation" models are liable to fraud and "Scam 2.0". It is an open research question whether, and under which circumstances, apomediation models work better than intermediation approaches, and how apomediation models can be made less susceptible to fraud.

Collaboration

Collaboration specifically means to connect groups of people with each other who have not, or have insufficiently, interacted with each other. In the "researcher" corner of the Medicine 2.0 triangle, this may refer to bringing together scientists using tools and approaches such as the ones described by Schleyer [22] or Falkman [23] in this theme issue. But it also involves encouraging collaboration between diverse user groups, including for example fostering public participation and engagement in research issues, and user engagement in health care decisions. Collaboration between researchers on one hand, and the public or health professionals on the other hand, also means improved possibilities for knowledge translation and getting research findings into practice.

Openness

Finally, I would argue that openness is another important and emerging theme to consider in the Web 2.0 context. On one level—the technical level—Web 2.0 stands for transparency, interoperability, open source, and open interfaces: "Don't lock me in", "my data belongs to me", "web as operating system", and "open up your API" are popular philosophies associated with Web 2.0. Personal Health Application platforms such as HealthVault and Google Health both have APIs for other applications to connect to.

XSL•FO
RenderX

What is perhaps most significant about this development is that the “openness” philosophy of Web 2.0 tools will also raise the expectations of the Facebook generation in terms of dealing with their health data. Web 2.0 savvy consumers will push the envelope and demand more than just an institutions-specific “portal” (also called “tethered PHR”) which allows them to view or access their data but not to do anything else with it. Patients 2.0 will demand full control over their data (as a minimum, XML export!). Many current Medicine 2.0 applications fall short in that regard, in that people can feed information into the system but can’t get it out again.

On another—societal—level, Medicine 2.0 also implies openness and transparency which enables access to other kinds of information and data the public has historically had limited access to, for example research and research data (open access journals, open data etc.), and which even allows the public to engage in the research process itself (open peer-review).

Conclusion

Openness being a key theme in Web 2.0, it is very appropriate that the *Journal of Medical Internet Research*—an open access journal—sponsors the first conference and publishes the first theme issue on Medicine 2.0. Regardless of what labels we attach to this emerging field, those interested in collaborative tools and empowerment of end users will find stimulating new perspectives for research and policy in both the conference and this theme issue. We also do not see this as a one-time event, as JMIR will continue to consider and publish submissions which fall into this area, and the Medicine 2.0 Congress is likely to be an annual event focusing on the latest technologies and societal developments to support the five themes. In analogy to what Tim Berners-Lee once said about Web 2.0—that it was “what the Web was supposed to be all along” [24])—we could also say that “Medicine 2.0 is what ehealth was supposed to be all along”, and fostering and encouraging these developments was why this journal was created in the first place.

Conflicts of Interest

The author is editor and publisher of JMIR, organizer of the Medicine 2.0 conference series, and is involved in several commercial and non-commercial projects which can be called Web 2.0 or Web 3.0 applications. He is also owner of the Medicine 2.0® Congress trademark.

Note: An early draft of this article was published as a blog entry [5].

Multimedia Appendix

Figure 5.

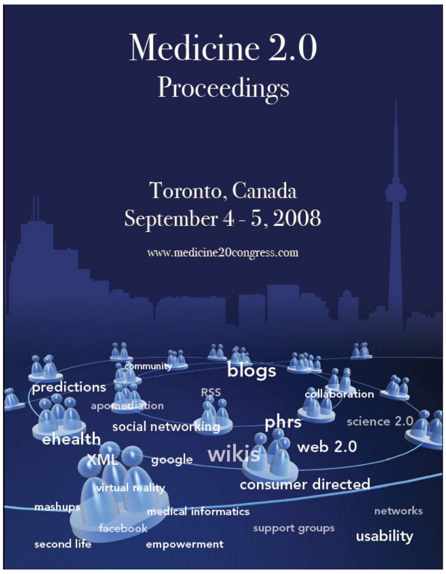

Eysenbach G (ed.). Medicine 2.0 Proceedings. Toronto, ON, Canada, Sept 4-5, 2008

[PDF file (Adobe Acrobat File), 605 kB - jmir_v10i3e22_app1.pdf]

References

1. Mandl KD, Kohane IS. Tectonic shifts in the health information economy. N Engl J Med 2008 Apr 17;358(16):1732-1737. [Medline: 18420506] [doi: 10.1056/NEJMsb0800220]
2. Bourgeois FT, Simons WW, Olson K, Brownstein JS, Mandl KD. Evaluation of influenza prevention in the workplace using a personally controlled health record: randomized controlled trial. J Med Internet Res 2008;10(1):e5 [FREE Full text] [Medline: 18343794] [doi: 10.2196/jmir.984]

XSL•FO
RenderX

3. Eysenbach G. Google Health starts pilot test at Cleveland Clinic - and my reflections on Personal Health Records 2.0 (PHR 2.0). Gunther Eysenbach Random Research Rants Blog. 2008 Feb 21. URL: http://gunther-eysenbach.blogspot.com [WebCite Cache ID 5WAZNYYli]
4. Eysenbach G. Medicine 2.0 Congress Website. URL: http://www.medicine20congress.com/ [WebCite Cache ID 5aJsnNo4C]
5. Eysenbach G. Medicine 2.0 Congress Website launched (and: Definition of Medicine 2.0 / Health 2.0). Gunther Eysenbach Random Research Rants Blog. 2008 Mar 06. URL: http://gunther-eysenbach.blogspot.com/2008/03/medicine-20-congress-website-launched.html [WebCite Cache ID 5aLi7UZUh]
6. Eysenbach G. Medicine 2.0 Final Program. Medicine 2.0 Congress. URL: http://www.medicine20congress.com/ocs/schedule.php [WebCite Cache ID 5aJtU3rUj]
7. Hughes B, Joshi I, Wareham J. Health 2.0 and Medicine 2.0: tensions and controversies in the field. J Med Internet Res 2008;10(3):e23 [FREE Full text] [Medline: 18682374] [doi: 10.2196/jmir.1056]
8. Davidson KP, Pennebaker JW. Virtual narratives: Illness representations in on-line support groups. In: Petrie KJ, Weinman JA, editors. Perceptions of Health and Illness. Amsterdam: Harwood Academic Publishers; 1997:463-486.
9. Eysenbach G. Consumer health informatics. BMJ 2000 Jun 24;320(7251):1713-1716. [Medline: 10864552]
10. Eysenbach G. The law of attrition. J Med Internet Res 2005 Mar 31;7(1):e11 [FREE Full text] [Medline: 15829473] [doi: 10.2196/jmir.7.1.e11]
11. Eysenbach G. An ontology of quality initiatives and a model for decentralized, collaborative quality management on the (semantic) World-Wide-Web. J Med Internet Res 2001 Dec 31;3(4):E34 [FREE Full text] [Medline: 11772549] [doi: 10.2196/jmir.3.4.e34]
12. Computerization of Medical Practices for the Enhancement of Therapeutic Effectiveness investigators, Willison DJ, Keshavjee K, Nair K, Goldsmith C, Holbrook AM. Patients' consent preferences for research uses of information in electronic medical records: interview and survey data. BMJ 2003 Feb 15;326(7385):373 [FREE Full text] [Medline: 12586673] [doi: 10.1136/bmj.326.7385.373]
13. Mandl KD, Szolovits P, Kohane IS. Public standards and patients' control: how to keep electronic medical records accessible but private. BMJ 2001 Feb 3;322(7281):283-287 [FREE Full text] [Medline: 11157533] [doi: 10.1136/bmj.322.7281.283]
14. Frost JH, Massagli MP. Social uses of personal health information within PatientsLikeMe, an online patient community: what can happen when patients have access to one another's data. J Med Internet Res 2008;10(3):e15 [FREE Full text] [Medline: 18504244] [doi: 10.2196/jmir.1053]
15. Heverly RA. Growing Up Digital: Control and the Pieces of a Digital Life. McPherson T, ed. Digital Youth, Innovation, and the Unexpected. The John D and Catherine T MacArthur Foundation Series on Digital Media and Learning Cambridge, MA: MIT Press; 2008.
16. Eysenbach G. Credibility of health information and digital media: new perspectives and implications for youth. In: Metzger MJ, Flanagin AJ, editors. Digital Media, Youth, and Credibility. The John D and Catherine T MacArthur Foundation Series on Digital Media and Learning. Cambridge, MA: MIT Press; 2008. URL: http://www.mitpressjournals.org/doi/pdf/10.1162/dmal.9780262562324.123 [doi: 10.1162/dmal.9780262562324.123]
17. Eysenbach G. From intermediation to disintermediation and apomediation: new models for consumers to access and assess the credibility of health information in the age of Web2.0. Stud Health Technol Inform 2007;129(Pt 1):162-166. [Medline: 17911699]
18. Eysenbach G, Diepgen TL. Towards quality management of medical information on the internet: evaluation, labelling, and filtering of information. BMJ 1998 Nov 28;317(7171):1496-1500 [FREE Full text] [Medline: 9831581]
19. Eysenbach G. Challenges and changing roles for medical journals in the cyberspace age: electronic pre-prints and e-papers. J Med Internet Res 1999 Dec 31;1(2):E9 [FREE Full text] [Medline: 11720918] [doi: 10.2196/jmir.1.2.e9]
20. Peer review and fraud. Nature 2006 Dec 21;444(7122):971-972. [Medline: 17183274] [doi: 10.1038/444971b]
21. Slotnik DE. Too Few Friends? A Web Site Lets You Buy Some (and They Are Hot). New York Times. 2007 Feb 26. URL: http://www.nytimes.com/2007/02/26/technology/26fake.html?_r=2&oref=slogin&oref=slogin [WebCite Cache ID 5aL53Dtz3]
22. Schleyer T, Spallek H, Butler BS, Subramanian S, Weiss D, Poythress ML, et al. Facebook for scientists: requirements and services for optimizing how scientific collaborations are established. J Med Internet Res 2008;10(3):e24 [FREE Full text] [Medline: 18701421] [doi: 10.2196/jmir.1047]
23. Falkman G, Gustafsson M, Jontell M, Torgersson O. SOMWeb: A Semantic Web-Based System for Supporting Collaboration of Distributed Medical Communities of Practice. J Med Internet Res 2008;10(3):e25 [FREE Full text] [Medline: 18725355] [doi: 10.2196/jmir.1059]
24. Berners-Lee T. IBM developerWorks pod cast, recorded 7-28-2006 (Transscript). IBM. IBM. URL: http://www-128.ibm.com/developerworks/podcast/dwi/cm-int082206.txt [WebCite Cache ID 5aL8Cp6Zf]

Abbreviations

API: application programming interface
PCHR: Personally Controlled Health Record
PHA: Personal Health Application

XSL•FO
RenderX

PHR: Personal Health Record

Edited by G. Eysenbach; This is a non-peer-reviewed article. Submitted 22.01.08; Published 25.08.08

Please cite as:
Eysenbach G
Medicine 2.0: Social Networking, Collaboration, Participation, Apomediation, and Openness
J Med Internet Res 2008;10(3):e22
<URL: http://www.jmir.org/2008/3/e22/>

XSL•FO
RenderX

Medicine 2.0
Proceedings

Toronto, Canada
September 4 - 5, 2008

www.medicine20congress.com

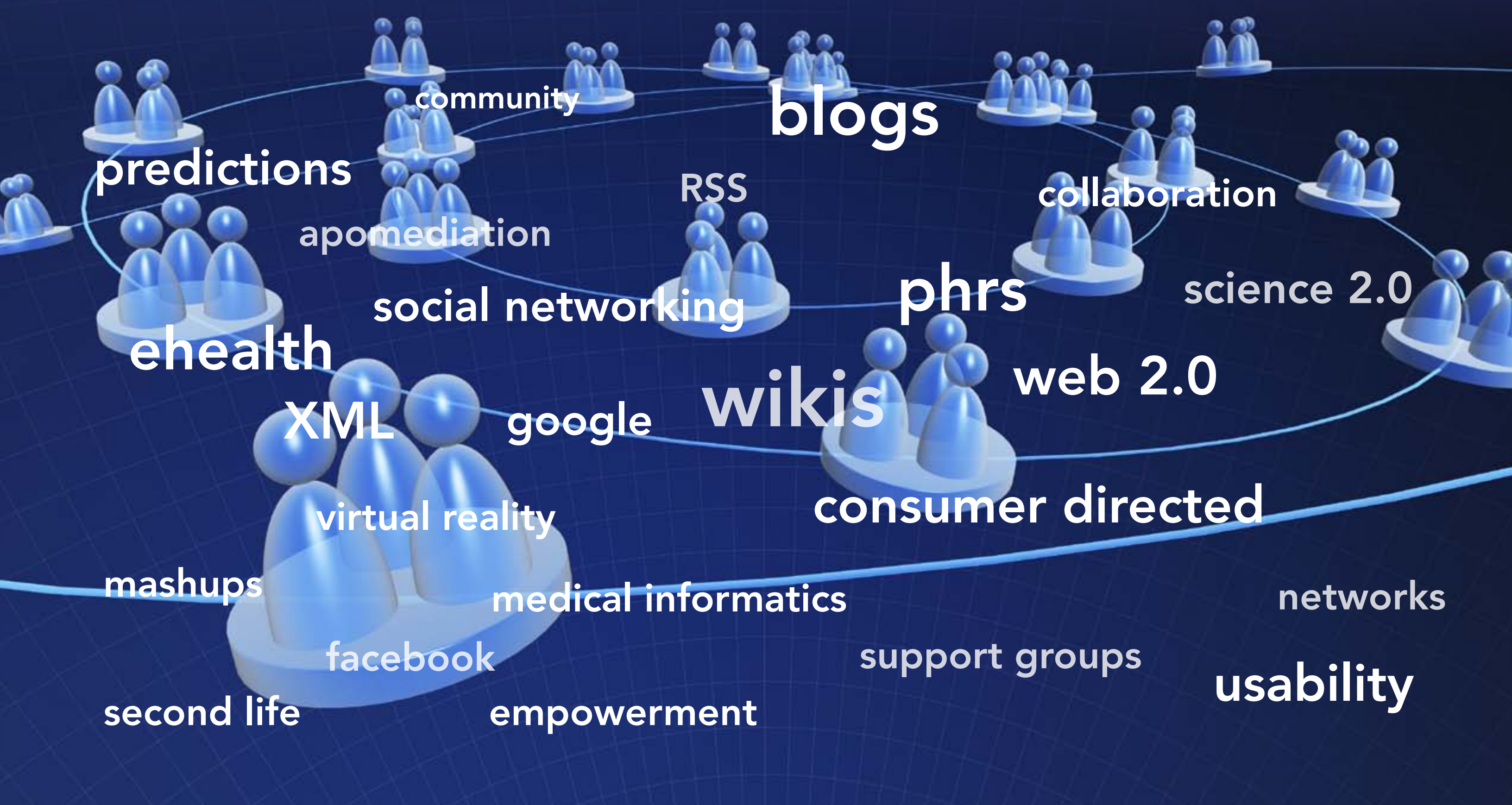

Table of Contents

9:00 AM - 9:30 AM IN MARS AUDITORIUM........6

9:30 AM - 10:00 AM IN MARS AUDITORIUM......6

TOPIC: BUILDING VIRTUAL COMMUNITIES AND SOCIAL NETWORKING APPLICATIONS FOR HEALTH PROFESSIONALS.....6

Asklepios: The CMA's new social networking site for Canadian physicians............................6

10:00 AM - 10:30 AM IN MARS AUDITORIUM....6

TOPIC: BUILDING VIRTUAL COMMUNITIES AND SOCIAL NETWORKING APPLICATIONS FOR PATIENTS AND CONSUMERS. 6

Social uses of personal health information within PatientsLikeMe™, an online patient community: What can happen when patients have access to one another's data....................6

11:00 AM - 12:30 PM IN MARS AUDITORIUM.....6

TOPIC: BLOGS...6

Inside the Health Blogosphere: Governance, Quality and the New Opinion Leaders.............6

Medical Bloggers Panel...................................7

11:00 AM - 12:30 PM IN MARS CR2........................8

TOPIC: CONSUMER EMPOWERMENT, PATIENT-PHYSICIAN RELATIONSHIP, AND SOCIOTECHNICAL ISSUES......................8

The Construction of Expertise in the Age of the Internet: Psychotropic Drug Knowledge in Consumer-Constructed Online Spaces.............8

BioTIFF: Articulating Self-Documenting Personal Health Digital Information Artefacts8

Hesitative Introduction of E-mail consultations in General Practice..9

Not every site needs a wiki: A conceptual framework for health Websites.......................10

11:00 AM - 12:30 PM IN MARS CR3.....................10

TOPIC: WEB 2.0 APPROACHES FOR BEHAVIOUR CHANGE, PUBLIC HEALTH AND BIOSURVEILLANCE...........................10

Preventive Medicine and the Allen Behavioral Change Model: A Collaboration for Health Behavior Change Using Web-based Interventions..10

E-epidemiology – adapting epidemiological data collection to the 21st century..................11

Personally Tailored Health Information: A Health 2.0 Approach.....................................12

Biosurveillance 2.0: A Social Networking Approach...12

1:30 PM - 3:00 PM IN MARS AUDITORIUM.......13

TOPIC: BUILDING VIRTUAL COMMUNITIES AND SOCIAL NETWORKING APPLICATIONS FOR PATIENTS AND CONSUMERS 13

Expert Patients as Apomediaries...................13

Web 2.0 systems supporting childhood chronic disease management: a general architecture compliant with the WHA eHealth Resolution.13

From Social Networks to Social Medicine: Exploring the role of online interventions......14

1:30 PM - 2:30 PM IN MARS CR2..........................15

TOPIC: DIGITAL LEARNING..15

Where can teens find health information? A survey of web portals designed for teen health information seekers.......................................15

Versatile, Immersive, Creative and Dynamic Virtual 3-D Healthcare Learning..................15

1:30 PM - 3:00 PM IN MARS CR3..........................15

Planning the Development of the Singapore National Health Portal..................................15

Providing Online Access to Point-of-Care Information: Lessons from the OntarioMD.ca Physician Portal..16

e-health in a Remote and Rural Setting in the North of Scotland- An Evolving Success Story16

Knowledge Exchange Nodes in Public Health: Two successful practical experiences............17

3:30 PM - 5:00 PM IN MARS AUDITORIUM.......18

TOPIC: BUILDING VIRTUAL COMMUNITIES AND SOCIAL NETWORKING APPLICATIONS FOR PATIENTS AND CONSUMERS 18

Patient Problem-Solving on the Web: How do Patients Use Web Forums to Cope with Chronic Disease?...18

Living with HIV/AIDS and use of online support groups...18

Moderated online social networking for smokers: Content of first posts from 2,562 members...19

Patient-initiated Online Support Groups: Motives for Initiation, Extent of Success and Success Factors...20

3:30 PM - 5:00 PM IN MARS CR2..........................20

TOPIC: WIKIS..20

Using Wiki for a Large Medical Nomenclature Translation..20

Patient-Driven Content Creation: Effectively Introducing Wiki Repositories to Virtual Support Communities..21

Collaborative Knowledge Translation: Application of a Wiki Model for Primary Care Practitioners..22

3:30 PM - 5:00 PM IN MARS CR3..........................22

TOPIC: WEB2.0-BASED MEDICAL EDUCATION AND LEARNING 22

The use of Web 2.0 in teaching Medical Informatics to postgraduate medical students: first experiences..22

Medicine 2.0 and Medical Faculty Development 23

Medical education and building an on-line reputation in the world of web 2.0.................23

The potential and challenges of Web 2.0 in the education of healthcare professionals............24

9:00 AM - 10:30 AM IN MARS AUDITORIUM....25

Methodological Issues and Challenges in eHealth Research Panel.................................25

9:00 AM - 10:30 AM IN MARS CR2.......................25

RNAO eChampion Program...........................25

Representing and Processing Dynamic Healthcare Workflow......................................26

Consumers are not Patient(s).........................26

MyOpenCare: Multicontent treatment paths to share medical knowledge and experience......27

9:00 AM - 10:30 AM IN MARS CR3.......................27

TOPIC: COLLABORATIVE BIOMEDICAL RESEARCH, ACADEMIC / SCHOLARLY COMMUNICATION, PUBLISHING AND PEER REVIEW 27

Development of an RSS-based Current Awareness Service..27

Implementation of Web 2.0-services in the Norwegian Electronic Health Library...........27

Online video-publication for biological research 28

11:00 AM - 12:30 PM IN MARS AUDITORIUM...28

TOPIC: CONSUMER EMPOWERMENT, PATIENT-PHYSICIAN RELATIONSHIP, AND SOCIOTECHNICAL ISSUES....................28

Study of the ePatient as a provider of health content in the Internet....................................28

Will the development of Web 2.0 technology result in a preference for quantity over quality? 29

Women Wading Through the Web: providing women with the tools to evaluate online health information..29

Empowerment by Participation in Online Support Groups for Patients with Arthritis, Fibromyalgia and Breast Cancer..................30

11:00 AM - 12:30 PM IN MARS CR2......................31

TOPIC: PERSONAL HEALTH RECORDS AND PATIENT PORTALS 31

Virtual Health Platform.................................31

InfoWell Patient Portal: A Case of Patient-Centred Design..32

Measuring Efficiency of Use in a Web-based EMR Developed for Malawi: Lessons Learned From Performing a Preliminary CogTool Analysis...32

User-centered Design of a PHR: Traditional Web Forms vs. Wizard Forms.........................33

11:00 AM - 12:30 PM IN MARS CR3......................34

TOPIC: BUILDING VIRTUAL COMMUNITIES AND SOCIAL NETWORKING APPLICATIONS FOR HEALTH PROFESSIONALS...34

Stop The World wide web I Want To Get Off..34

Creating and Supporting a Community of Practice...34

New Media Medicine: A Social Network for Doctors and Medical Students.......................34

Facilitating Online Collaboration and Communication: A Groups Approach............35

1:30 PM - 3:00 PM IN MARS AUDITORIUM.......35

TOPIC: CONSUMER EMPOWERMENT, PATIENT-PHYSICIAN RELATIONSHIP, AND SOCIOTECHNICAL ISSUES....................35

Using Technology to Engage Patients and Clinicians in Electronic Cancer Symptom Assessment and Management: Lessons Learned and Implications for Practice........................35

Developing of a web-based application to facilitate patient treatment adherence in individuals with sleep apnea treated with Continuous Positive Airway Pressure (CPAP) devices...35

Web and mobile solution for support of process of care: case of depression............................36

Evaluation of the use of an interactive web-based support program for optimizing the management of Diabetes Mellitus.................37

1:30 PM - 3:00 PM IN MARS CR2.........................37

TOPIC: SEMANTIC WEB ("WEB 3.0"), OPEN SOURCE.....37

PESCA: Developing an Open Source Platform To Bring eHealth To Latin America and the Caribbean..37

*"Nexthealth" - An Open-Source, Web-Based Tool Determining "What's Next" in the Evolution of Consumer-Centric Care Delivery*38

Usage of Semantic Web Technologies (Web-3.0) Aiming to Facilitate the Utilisation of Computerized Algorithmic Medicine in Clinical Practice..38

1:30 PM - 3:00 PM IN MARS CR3..........................39

TOPIC: WEB 2.0 APPROACHES FOR CLINICAL PRACTICE, CLINICAL RESEARCH, QUALITY MONITORING.....................39

To 2.0 or not to 2.0 – have junior doctors already answered the question?.....................39

MDPIXX: The Global Medical Images Repository..39

Social Computing Enhances Biomedical Research on Rare Diseases............................40

Mobile Medicine 2.0......................................41

3:30 PM - 4:30 PM IN MARS AUDITORIUM.......41

TOPIC: ETHICAL & LEGAL ISSUES, CONFIDENTIALITY AND PRIVACY...41

Risk 2.0: The future of connected health (Panel) 41

POSTER PRESENTATIONS (FOYER).................43

THURSDAY 10:30 AM – 11:00 AM........................43

Using Web 2.0. tools on health Websites in Spanish...43

Email Communication Between Physicians and Patients..43

Information search behaviours in a controlled study of a web portal for young diabetes type 1 patients and their parents...............................43

Is www.forumclinic.org an electronic support groups for patients with chronic illnesses?....44

Using usability-tests to improve language match of a web-based system for digital triage in primary care..45

Internet Use among Occupational Therapists in Their Clinical Practice..................................45

Health professionals' attitudes toward using an Internet portal for child and adolescent diabetes care..46

Determinants of engagement in face-to-face and online patient support groups........................46

The EMR-scan: Assessing the Quality of Electronic Medical Records in General Practice 47

9:00 AM - 9:30 AM in MaRS Auditorium

Greetings from the International Medical Informatics Association (IMIA), and CHIRAD,
Peter J. Murray, IMIA Vice President for Strategic Planning

Welcome from the Conference Chair
Gunther Eysenbach, Senior Scientist, Centre for Global eHealth Innovation, Toronto; Editor/Publisher, Journal of Medical Internet Research (JMIR)

9:30 AM - 10:00 AM in MaRS Auditorium

Topic: Building virtual communities and social networking applications for health professionals

Pat Rich, Director CMA Online Content, Canadian Medical Association (CMA)

Asklepios: The CMA's new social networking site for Canadian physicians

(Practice)

In August 2008, the CMA will be launching Asklepios, its social networking site for Canadian physicians, medical students and residents in Canada, which will be a very significant development for enabling the use of web 2.0 technologies by Canadian doctors. This talk presents an early first look at Asklepios. Asklepios is an online community that allows physicians to make new contacts, get advice from colleagues, and collaborate with physicians all over the country.

10:00 AM - 10:30 AM in MaRS Auditorium

Topic: Building virtual communities and social networking applications for patients and consumers

Jeana Frost, PatientsLikeMe; Michael Massagli, PatientsLikeMe

Social uses of personal health information within PatientsLikeMe™, an online patient community: What can happen when patients have access to one another's data

(Research)

Background: This project investigates the ways in which patients respond to the shared use of what is often considered private information: personal health data. There is a growing demand for patient access to personal health records. The predominant model for this record is a repository of all clinically relevant health information kept securely and viewed privately by patients and their healthcare providers. While this type of record does seem to have beneficial effects for the patient–physician relationship, the complexity and novelty of these data coupled with the lack of research in this area means the utility of personal health information for the primary stakeholders -- the patients —is not well documented or understood. **Objective**: PatientsLikeMe™ is an online community built to support information exchange between patients. The site provides customized disease-specific outcome and visualization tools to help patients understand and share information about their condition. We begin this paper by describing the components and design of the online community. We then identify and analyze how users of this platform reference personal health information within patient-to-patient dialogues. **Methods**: Patients diagnosed with amyotrophic lateral sclerosis (ALS) post data on their current treatments, symptoms, and outcomes. These data are displayed graphically within personal health profiles and are reflected in composite community-level symptom and treatment reports. Users review and discuss these data within the Forum, private messaging, and comments posted on each others' profiles. We analyzed member communications that referenced individual-level personal health data to determine how patient peers use personal health information within patient-to-patient exchanges. **Results**: Qualitative analysis of a sample of 123 comments (about 2% of the total) posted within the community revealed a variety of commenting and questioning behaviors by patient members. Members referenced data to locate others with particular experiences to answer specific health-related questions, proffer personally acquired disease-management knowledge to those who are most likely to benefit from it, and foster and solidify relationships based on shared concerns. **Conclusions:** Few studies examine the use of personal health information by patients themselves. This project suggests how patients who choose to explicitly share health data within a community may benefit from the process, helping patients engage in dialogues that may inform disease self-management. We recommend that future designs make each patient's health information as clear as possible, automate matching of people with similar conditions and using similar treatments, and integrate data into online platforms for health conversations.

11:00 AM - 12:30 PM in MaRS Auditorium

Topic: Blogs

Neil Seeman, School of Health Services Management, Ryerson University & IBM Healthcare

Inside the Health Blogosphere: Governance, Quality and the New Opinion Leaders

(Research)

Introduction: Although health-related blogs are exploding in number on the Web, there has been very little examination of the governance of these blogs. In

light of criticism from various sectors (especially from prominent members of the lay media, and from the clinical community) over the quality and governance of health blogs, I wished to undertake an empirical analysis of the governance of the most popular health blogs on the Web. Further, I wished to compare the content of health blogs to the health-related content of the most popular Canadian newspapers to determine whether blogs offer greater opportunity for consumers to obtain relevant health news content.**Methods:** Based on a relational link analysis and a review of the number of active subscribers, I identified the highest density health blogs on the Web. I then created a "blog governance score" based on a number of governance best practices (incl. user privacy protection and content objectivity). I also analyzed the findings to determine the quality of the content of health blogs as compared to lay media health coverage (i.e., in newspapers). This was done by reviewing comparative coverage of the 10 most important medical news stories of 2007 as defined by a consensus of more than 60 clinical experts across North America.**Results:** Highly trusted health blogs are mostly "start-ups" that win loyalty via a combination of first-to-market prominence, niche and randomness. Very few highly used health blogs are affiliated with a major media company. Further, a higher governance score does not necessarily correlate with higher global usage (i.e., traffic rank). When reviewing the content of popular health blogs, the following observations can be made: (i) both lay newspapers and health blogs fare poorly on reporting and discussing medical "news that matters." General interest blogs fare as well or better than general interest Canadian newspapers in reporting critical medical stories; (ii) only a small minority of popular health blogs demonstrate overt political partisanship or drug industry sponsorship; (iii) a minority of popular blogs contain prominent user privacy controls in keeping with best practices; (iv) a majority contain some general industry sponsorship; (v) a majority have semi- or full moderation by subject matter experts. **Discussion:** Health blogs offer promise in the delivery of engaged interaction for consumers seeking relevant health information. The findings suggest that such blogs are generally well-governed or monitored by subject matter experts and are free from politically partisan bias. Although improved user privacy protocols are necessary, there is little indication that these popular blogs contain personal health data that are linkable to any individual.

Bertalan Mesko, University of Debrecen, Debrecen, Hungary; Jennifer Mccabe Gorman, Health 2.0/Nexthealth; Keith J Kaplan; Sam Solomon; Peter Murray

Medical Bloggers Panel

(Practice)

The Medical Bloggers' Panel aims to give deeper insight into medical blogging; why it is beneficial to maintain a medical blog and how one can start their own blogs. The Panel also plans to discuss the dangers web 2.0 can cause to medical professionals in the medical blogosphere and to present some medical blogging careers.

Medical blogs allow the hospital, medical, and health/wellness management communities to build bridges to populations and individuals that have traditionally existed as the 'other' waiting in the wings - peoples at the periphery of medical practice, including patients.

Blogs are communication vectors that, properly harnessed via distinctive voices, allow us to connect to diverse constituencies. These virtual conversations often move offline into the realm of practical, everyday strategy development, improving the overall practice of medical research and care delivery across an increasingly diverse and fragmented care continuum.

Jennifer McCabe Gorman of Health Management RX (healthmgmtrx.blogspot.com) will discuss the role of medical blogs in terms of creating open lines of communication that allow the medical community to inexpensively cross - pollinate best practices from outside industries.

Sam Solomon of the National Review of Medicine's editors' blog, Canadian Medicine (canadianmedicine.blogspot.com), will provide a Canadian perspective on medical blogging and discuss the question of anonymous authorship.

Keith Kaplan of the Digital Pathology Blog (www.tissuepathology.typepad.com) will discuss the role of blogging about technologies and issues potentially disruptive to conventional practice in pathology. He will also explore applications blogs may have for a platform to share content beyond stories and ideas.

Peter Murray from Health Informatics Blog (www.hi-blogs.info) will present experiences of developing collaborative models of blogging to support professional development, and discuss how such collaborative models could help support communities of practice for health professionals, patients and others.

Bertalan Meskó from Scienceroll (www.scienceroll.com) will talk about the advantages and dangers of medical blogging through the story of his blog. He will also discuss the career and networking opportunities a medical blog can provide from a medical student's perspective.

11:00 AM - 12:30 PM in MaRS CR2

Chair: Derek Hansen

Topic: Consumer empowerment, patient-physician relationship, and sociotechnical issues

Shannon Hughes, School of Social Work Florida International University, Miami, FL, USA; David Cohen, School of Social Work Florida International University, Miami, FL, USA

The Construction of Expertise in the Age of the Internet: Psychotropic Drug Knowledge in Consumer-Constructed Online Spaces

(General)

By universal definition and consensus, information about psychotropic drugs prescribed and used as medicines rests on a highly specialized knowledge base, long seen as the legitimate if not exclusive province of medical experts. This expert knowledge base has, however, become highly contested, for three main reasons. First, to reach a "valid" understanding of drugs' effects, researchers have persistently muted the direct voice and speech of the patient in favor of a presumably more objective biomedical discourse [1]. Second, the blurring lines between pharmaceutical industry marketing and scientific activities, and the near monopoly of the industry over "scientific" knowledge production on psychotropic drugs has resulted, at a minimum, in serious publication biases and great uncertainty about drug efficacy and safety [2, 3]. Third, intermediaries through which officially legitimated drug information is filtered (including the FDA drug label, psychiatrists, and direct-to-consumer advertising) are losing credibility and relevance. In parallel, the Internet now allows researchers, clinicians, and consumers to review drug experiences and information from thousands of consumers without professional mediation, and presumably without pharmaceutical industry supervision over content. How these web-based sources contribute to or dilute the expert/scientific knowledge base, or how they constitute their own knowledge base, remains virtually unexplored. Moreover, pharmaceutical companies looking for new opportunities to influence consumer behavior are keenly aware of the power of consumer forums to build trust, offer support, and encourage behavior [4]. This presentation will also review the possible extent and implications of drug representative infiltration into online consumer spaces.Finally, "democratizing" the production of knowledge and consumers' broadened acceptance of what constitutes legitimate, trustworthy, or credible information results in a trans-disciplinary debate about the appropriate role of experts and non-experts in policy, media, science, and culture, as well as the true nature of what is being applauded as "democratization" [5]. Applied to the construction of knowledge about psychotropic drugs, the debate may be summarized by the following questions: Are increased pluralism and democracy in drug knowledge production and decision-making, along with increased transparency of such processes, the best means to build a knowledge base continually evolving toward the ideal of being "fully informed"? Or might they actually hinder both experts' and laypersons' understanding of the genuine effects of drugs, by diluting the knowledge base with ill-considered contributions? Answers to these questions will have broad implications for the future of clinical research methods, post-marketing drug surveillance, and treatment decision-makers (clinicians, third-party payers, and potential and actual consumers).

This presentation will review inadequacies in how scientific methods are applied in drug research to construct official drug knowledge, opportunities for knowledge construction within consumer-constructed online spaces, and emerging questions related to the authenticity and credibility of peer-produced, often anonymous contributions.

References

1. Jacobs D, Cohen D. What is really known about psychological alterations produced by psychiatric drugs? Intern J of Risk & Saf in Med 1999;12:37-47.

2. Ioannidis I, Lau J. Completeness of safety reporting in randomized trials: An evaluation of 7 medical areas. JAMA 2001;285:437-443.

3. Melander H, Ahlqvist-Rastad J, Meijer G, Beermann B. Evidence-b(i)ased medicine - selective reporting from studies sponsored by pharmaceutical industry: Review of studies in new drug applications. Br Med J 2003;326:1171-1173.

4. O'Neill A. The patient trust deficit in pharmaceutical marketing. DTC Perspectives 2007 Dec:12-16.

5. Keen A. The cult of the amateur: How today's internet is killing our culture. New York: Doubleday; 2007.

Peter Pennefather, Leslie Dan Faculty of Pharmacy, University of Toronto, Toronto ON, CANADA; West Suhanic, Leslie Dan Faculty of Pharmacy, University of Toronto, Toronto, ON CANDA

BioTIFF: Articulating Self-Documenting Personal Health Digital Information Artefacts

(Develop)

Although the Web 2.0 world of participatory, web-based collaboration and social networking has just been launched, a Web 3.0 is emerging where ubiquitous data and virally distributed free and open applications for interacting with that data, is leading to new and useful forms of socio-technical articulation. The University of Toronto Laboratory for Collaborative Diagnostics (www.lcd.utoronto.ca) is developing an electronic personal health record (ePHR) system that anticipates Web 3.0. The BioTIFF is a self-documenting digital file structure designed to bind typical ePHR clinical information to digital files that record results of diagnostic tests. It consists of a highly extensible multi-page tagged image file format (TIFF) record in which digital image representations of the results of diagnostic tests can be annotated with richly informative metadata providing the substrate for generating bio-referenced

overlay maps useful in interpreting that information. For example, the first digital CT image of a newly diagnosed brain tumor can be entered into an articulated and indexable person-centered digital file envelope containing all of the persons relevant health diagnostic data and metadata as well as pointers to bioinformatic and eHealth information concerning tumors of that type. What is more, this image can act as a navigation beacon and record-anchor for asynchronous communication, info(r)mediation and other forms of transactions within the persons circle of care concerning the tumor. The BioTIFF is inspired by GeoTIFF (www.remotesensing.org/geotiff/geotiff.html) that evolved as an open standard for creating multi-page TIFF relating overlapping maps and images of particular geospatial locations and linking those maps to associated metadata. GeoTIFF exploited the self-documenting properties of geographical coordinate systems to uniquely identify a location within a satellite image and thereby allow cross-referencing to other information artefacts relevant to that location. The BioTIFF approach uses molecular, cellular, anatomical, and biomedical coordinate systems to specify biological inner space domains where biological systems dynamics happen in ways that express themselves as health and disease. These coordinate systems allow mapping of the progression of treatment and of health status in individuals as represented by the continuous stream of results from diagnostic and laboratory tests guiding treatment. This biomedical mapping can be further articulated with socio-technical systems frameworks describing how individuals interact with technologies and places in their healthcare systems. In this way the BioTIFF record can contain all of the information needed to drive interpretive applications aimed at mapping a persons experiences with their treatment. The BioTIFF is not only self-documenting but also enables members of the circle of care, especially the person at the center, to document, reflect, and comment upon opinions and narratives within the circle of care concerning goals, experiences and interpretation of outcomes. This enables a multilateral, dialogical, and person centric approach to negotiating optimized health care trajectories. The BioTIFF has been designed as a distributed and easily scalable and transportable application that is well adapted to emerging commoditized cloud storage and computational infrastructure. In this talk we describe BioTIFF´s architecture and design constraints.

Robert Verheij, Neth. Inst. for Health Services Reseach (NIVEL); Carmen Ton; Kiek Tates, Neth. Inst. for Health Services Research (NIVEL)

Hesitative Introduction of E-mail consultations in General Practice

(Research)

IntroductionThe Dutch Council for Public Health and Health Care reported in 2005 that 70% of internet users would want to have the opportunity to consult their own general practitioner by e-mail [1]. Since January 1, 2006, general practitioners in the Netherlands are reimbursed 4.50 euro for e-mail consultations, on the condition that it does not concern a new health problem and that it substitutes for a normal practice consultation. In this presentation we will investigate how many doctors and patients actually use e-mail consultations; whether the health problems presented in e-consultations differ from normal practice consultations; and to what extent patients who use e-consultations are different in terms of, age, gender and frequency of use of GP services. **Methods**Data are derived from routine electronic medical records of 100 GP practices that participate in the Netherlands Information Network of General Practice (LINH) [2]. The dataset comprises all 1.902.902 contacts of all 271.509 patients listed in these practices in 2006. For each contact the dataset holds information on type of contact; patient age and gender, prescribed drugs, referrals, as well as diagnosis information. Diagnoses are recorded using the International Classification of Primary Care (ICPC) coding system. **Results** E-mail consultations were claimed at least once in about 30% of the practices involved. Health centers were overrepresented as compared with single handed practices. In most practices the number of consulations was less than 25 in the whole study period, but there were also some enthousiasts: three practices were responsible for almost 50% of all 1159 e-consultations that were claimed. These consultations pertained to 777 patients. For most patients (74%) an e-mail consultation was claimed only once. Taking into account the fact that women use more GP services in general, more men than women were users of e-consultations. Among users of of e-mail consultations people aged 25 - 44 years are overrepresented. In a relatively large number of e-mail consultations (73%) no diagnosis was recorded. Where this information was available, metabolic diseases such as diabetes, pregnancy etc and diseases/complaints of the male reproductive system were recorded more often than in normal practice consultations. Cardiovascular problems and respiratory problems seemed to be less suitable for e-mail consultations.Among users of e-mail consultations, the contact frequency for the GP is 65% higher than among other people who consulted their GP in the study period. Relatively many users of e-consultations, had a normal consultation in the days before and after the e-consultation. **Conclusion**Though many people seem to be interested in e-consultations, still very few are people using it. In relative terms, e-mail consultations are used more often by men than women and by people aged 25-44. The fact that e-consultations are more often used by frequent attenders suggests that e-consultations are a particularly interesting option for those who have a close relation with their GP. Additional results for 2007 will be presented at the conference.

[1] AJG Van der Rijen. Internetgebruiker en Veranderingen in de Zorg. Zoetermeer, Raad voor de Volksgezondheid en Zorg, 2005.

[2] Verheij RA, et al. Netherlands Information Network of General Practice. NIVEL/WOK. 2008-05-02. URL:http://www.nivel.nl/linh/. Accessed: 2008-05-02. (Archived by WebCite® at http://www.webcitation.org/5XVf8Yx9M)

Holly Witteman, Human Factors, Mechanical and Industrial Engineering & Health Care, Technology and Place, University of Toronto, Toronto, Canada; Laura O'Grady, Health Policy, Management and Evaluation, Faculty of Medicine; Centre for Global eHealth Innovation & Health Care, Technology and Place, University of Toronto, Toronto, Canada

Not every site needs a wiki: A conceptual framework for health Websites

(Research)

IntroductionIn the early days of the World Wide Web, a Website was a Website was a Website. Sites facilitated information exchange in the form of text, images and hyperlinks and, for the most part, differed from each other only in content and aesthetic design features [1]. As the Web and its associated technology evolved, exchanges became more complex, incorporating new functions such as commerce, which enabled users to purchase items or services via the Web, and new features such as guest books, which allowed readers to comment directly on Websites authored by others. This increase in interactivity continued, and there are now many different classes of Web-based applications, each with its own focus and key functions. Choosing which applications and technologies to incorporate in health Websites requires an understanding of one's goals and of how different Web-based tools might help achieve such goals. Similarly, analyzing existing Websites requires an understanding of fundamental differences in focus between different applications [2].**Methods**In this work, we drew on literature from Human-Computer Interaction, Sociology, Human Behaviour and eHealth to develop a conceptual framework for Web-based applications and technologies within which to consider and balance potentially competing goals and priorities. We explore these concepts through a framework with two pillars: 1) Community and 2) Information (Re)Organization that shape a structure of attributes, each with technical, design, social and human aspects.

Results

1) Community:

a) Individuals and Identity: What are the characteristics and roles of individuals in the community? How is identity constructed? What options are available for depicting identity?

b) Connections: What role do connections between individuals have in the application? How are connections made and represented?

c) Authorship: Who authors information in this application? How is authorship depicted?

d) Authority: What are the formal and informal structures of authority in the community? How do these reflect, reproduce, support, or provide alternatives to existing power structures?

e) Dialogue and Dissent: How is dialogue represented in the application? (How) is dissent addressed?

f) Collaboration: How does the application support collaboration amongst community members?

2) Information (Re)Organization

a) Information (Input): What are the characteristics of the content input to this application? E.g. Is the content textual or numerical? Scientific or lay? Does it convey specific experiential knowledge or generalized knowledge? Is it detailed or vague?

b) Organization (Presentation): How is the content of this application presented? E.g. Are there tables or graphs? Images, audio, or video? What is the general layout? Is it multi-platform?

c) Reorganization (Output): What are the characteristics of the content output from this application? E.g. Is it aggregated or mashed up from a variety of sources?

DiscussionWe propose a theoretical model describing the relationships between the attributes, situate existing and upcoming Web-based eHealth applications within the framework, and highlight the implications of this framework for eHealth researchers and health Website designers.

References

1. Berners-Lee T. Weaving the Web: The Original Design and Ultimate Destiny of the World Wide Web. New York, NY: Harper Collins; 2000.

2. Weiss JB, Campion TR. Blogs, Wikis, and Discussion Forums: Attributes and Implications for Clinical Information Systems. MEDINFO 2007 - Proceedings of the 12th World Congress on Health (Medical) Informatics: Building Sustainable Health Systems. Brisbane, Australia: IOS Press; 2007;12(Pt 1):157-161.

11:00 AM - 12:30 PM in MaRS CR3

Chair: Joan Dzenowagis

Topic: Web 2.0 approaches for behaviour change, public health and biosurveillance

Michael W. Allen, Medical School, U of MN, Minneapolis, USA; Edward Allan Murphy; David O. Wiebers; Charles P. Bosmajian, Jr.; Will Thalheimer; Julie Dirksen

Preventive Medicine and the Allen Behavioral Change Model: A Collaboration for Health Behavior Change Using Web-based Interventions

(Business)

The prevalence of chronic disease and the principal risk factors that lead to disease onset can be significantly diminished through lifestyle choices people make in their everyday lives. Interactive Web-based media has the capacity to assist millions of people to adopt healthy lifestyle practices through very individualized guidance tailored to each person with whom it interacts.

This paper describes a new paradigm for Web-based health behavior change developed through a collaboration between Preventive Medicine, a healthcare informatics company, and Allen Interactions,

a leading developer of custom e-learning applications. The concept is designed to empower individuals to integrate preventive healthcare strategies into their daily lives and inexpensively maintain frequent contact to monitor and reinforce such behavior.

This model offers a distinctive mix of medical and behavioral science, presented in a non-intimidating, interactive system. It provides interesting, engaging, and informative features that tailor the behavior health change process to each individual to help them overcome the inherent difficulties in the behavioral and lifestyle change process. A variety of behavioral science and information technology innovations designed to engage individuals continuously in their own personalized prevention and lifestyle plans will be described.

Health behavior change can use the viral characteristics of Web 2.0 which enable the creation of large social networks sharing common interests and providing mutual support. The explosion of social networks speaks to the popularity of network communication and the developing research base speaks to its effectiveness in supporting behavior change. The model also describes methods for creating frequent personalized contact with users on a large scale using a wide range of web-based applications.

Contrasting with other models, which emphasize rational thought and make reasoned cases that individuals should adopt healthier behaviors, this model focuses on the emotional and automatic underpinnings of the behavioral choices that people make. Methods that help users connect emotionally with interactive media are described. Strategies are presented for maintaining interest and reducing attrition, including the use of intrigue, surprise, and an emphasis on graphical rather than textual interactions. While message tailoring is used by some web-based applications, it is suggested that to make full use of the interactive capabilities of Web 2.0, strategies informed by e-learning research and experience should be more fully utilized. Methods of tailoring messages to individuals using algorithms based on unique characteristics of users are described. The model also describes an interactive and intriguing process of assessment that goes beyond simply transferring paper and pencil instruments to a computer screen as is commonly done in many of today's web-based applications.

By combining the capabilities of the Web 2.0 environment with a new model of behavior change based on years of e-learning research and development, a significant increase in effectiveness over current web-based health behavior change efforts can be obtained.

Christin Bexelius, Department of Medical Epidemiology and Biostatistics; Karolinska Institutet, Stockholm, Sweden; Jan-eric Litton, Department of Medical Epidemiology and Biostatistics; Karolinska Institutet, Stockholm, Sweden

E-epidemiology – adapting epidemiological data collection to the 21st century

(General)

We aim to present the prerequisites to use cell phones and SMS as a means of contact in LifeGene. LifeGene (www.lifegene.se) is a Swedish population-based prospective resource on lifestyle, health and genomics. The cohort will include at least 500.000 Swedes, starting with index person 18-49 years old and their household. This cohort will regularly (annually) be contacted to assess in depth exposure information through electronic means (web, cell phones, etc). There will be regular surveillance of morbidity through population based registers, and study participants will be contacted to assess symptoms for outcomes not typically reported in national health registers or ascertained via hospital discharge (e.g. acute infections, psychiatric disorders and allergy).

The rapid transformation of the society during the last decade has altered the possibilities for data collection in epidemiological studies (1-3). The modern society constantly generate new trends, and people change site and habits more often then twenty and even ten years ago. The traditional approaches, face-to-face interviews and paper questionnaires, are restricted to fixed sites and are costly in terms of time-delay and administration (4, 5). Introduction of new information and communication technologies such as the web, Interactive Voice Response (IVR) and cell phones hold great potential as innovative tools for medical data collections (6-9). E-epidemiology is the science underlying the acquisition, maintenance and application of epidemiological knowledge and information using digital media, and hold the potential to adapt medical data collection to a modern society (10). Using digital technologies, self-reported data through questionnaires can be replaced by objective measurements resembling data gained at health care services. The high access of cell phones gives a possibility to use the device for smaller questionnaires. Many new mobile phones include GPS (Global Positioning System) and JAVA technology, which gives ever more opportunities for real-time data collection.

Sweden has today among the highest Internet and cell phone penetration in the world, making the population suitable for introducing new technologies in data collection (11). The Department of Medical Epidemiology and Biostatistics (MEB) at Karolinska Institutet in Sweden has since 2001 designed and evaluated several studies using e-epidemiology. In 2003, the first large scale web-based study including 47,859 women in the age group 41-60 was conducted (12). Today, the department has used the Internet for several large scale population-based studies, including an Internet based-hearing test and a surveillance system utilizing IVR and a web-based application.

Experiences of using cell phones include sending SMS for identifying influenza vaccination coverage among the Swedish population and daily self-report of physical activity level trough a JAVA based questionnaire. In a previous study, we have studied the feasibility of using SMS in a study population between 18-49 years. Our results indicate that no bias is introduced when using SMS compared to telephone interviews and that data collected is comparable, based on socio-economic and demographic features gained from Swedish population based registers.

1. Dillman D. Presidential Address: Navigating the Rapids of Change: Some Observations on Survey Methodology in the Early Twenty-First Century. Public Opin Q 2002;66:473-494.
2. Couper MP. Technology Trends in Survey Data Collection. Social Science Computer Review 2005;23:486-501.
3. Kempf AM, Remington PL. New challenges for telephone survey research in the twenty-first century. Annu Rev Public Health 2007;28:113-26.
4. Siemiatycki J. A comparison of mail, telephone, and home interview strategies for household health surveys. Am J Public Health 1979;69:238-45.
5. Voigt LF, Koepsell TD, Daling JR. Characteristics of telephone survey respondents according to willingness to participate. Am J Epidemiol 2003;157:66-73.
6. Sax LG, SK; Bryant, AN. Assessing response rates and nonresponse bias in Web and Paper surveys. Reaserch in Higher Education 2003;44:409-432.
7. Mundt JC, Moore HK, Bean P. An interactive voice response program to reduce drinking relapse: A feasibility study. J Subst Abuse Treat 2006;30:21-9.
8. Fricker S, Galesic M, Tourangeau R, Yan T. An Experimental Comparison of Web and Telephone Surveys10.1093/poq/nfi027. Public Opin Q 2005;69:370-392.
9. Brick JM, Brick PD, Dipko S, Presser S, Tucker C, Yuan Y. Cell phone survey feasibility in the U.S.: Sampling and calling cell numbers versus Landline numbers. Public Opin Q 2007;71:23-39.
10. Ekman A, Litton JE. New times, new needs; e-epidemiology. Eur J Epidemiol 2007;22:285-92.
11. ITU/UNCTAD 2007 World Information Society report: Beyond WSIS. In: (ITU) ITU, ed. Geneva: International Telecommunication Union (ITU), 2007:1-175.
12. Ekman A, Dickman PW, Klint A, Weiderpass E, Litton JE. Feasibility of Using Web-based Questionnaires in Large Population-based Epidemiological Studies. Eur J Epidemiol 2006;21:103-11.

Jason Bonander, Centers for Disease Control and Prevention, Atlanta, GA USA

Personally Tailored Health Information: A Health 2.0 Approach

(General)

At present, strategies used for health tailoring typically require users to complete extensive questionnaires on health and behaviors to tailor sites to health needs. This poses formidable barriers to delivery of tailored messages. Are there alternative means of collecting online behavioral information and increasing the availability of highly tailored health information for health consumers that does not require lengthy and time consuming surveys? The presentation will address early results in testing the following hypothesis: can information contained on an individual's social networking page(s) be used to generate meaningful tailored health messages? The approach to be discussed uses publicly shared information contained in individuals' online profile(s) in social networking sites to tailor health information. Through an opt-in mechanism and connecting the dots within and across individuals' "online networked behavioral life" a tailored health profile could be presented to an individual. Such a tailored health profile could address any number of highly contextually relevant health issues, issues that likely relate to many public health priorities. The most salient public health themes for tailored information are likely to revolve around social, emotional and mental health, sexual and reproductive health/HIV and STD prevention, substance abuse, nutrition, physical activity, chronic and infectious disease prevention, and injury prevention. With an eye to the future, it has the potential to become a very personal facet of an overall electronic personal health record – one that could conceivably connect to other sources of personal health and medical information.

Taha Kass-hout, Assistant Professor, Emory University, Department of Biostatistics and Bioinformatics, Atlanta, GA, USA. InSTEDD, Director of Health Informatics, Palo Alto, CA, USA

Biosurveillance 2.0: A Social Networking Approach

(General)

While not an entirely new concept, biosurveillance has been gaining importance in the modern world. In recent history, the geographic isolation between plants and animals has been gradually broken by the intentional or natural transport of organisms through human travel, tourism or trade. Today, the rate at which species are moving between different bio-geographic regions is unprecedented, resulting in adverse ecological, economic, and human health consequences.

Additionally, global environmental changes have continued to grow rapidly throughout the past five years. These changes to climate, transport networks, disease pathogens and their vectors do not respect administrative boundaries, and their influences and impacts are best addressed on a global scale. These factors have contributed to an environment where new disease threats can spread globally within hours or days. This requires biosurveillance systems to be cross-disciplinary and to support global collaboration. Over the last decade, the majority of the designs, analyses and evaluations of these systems have been geared towards specific data sources and detection algorithms. Much less effort has been focused on how these systems will "interact" with humans. For example, consider multiple domain experts working at different levels across different organizations in an environment

where numerous biosurveillance algorithms may provide contradictory interpretations of ongoing events. The aim of this presentation is to discuss the anticipated contribution of social networking and collaborative technologies to address these emerging issues.

1:30 PM - 3:00 PM in MaRS Auditorium

Chair: Carol Bond

Topic: Building virtual communities and social networking applications for patients and consumers

Judy Proudfoot, Black Dog Institute, University of New South Wales, Sydney, Australia

Expert Patients as Apomediaries

(Research)

IntroductionBipolar disorder is chronic condition associated with significantdisability, with recent estimates suggesting a lifetime risk of 5%. Relapse rates are high. Little is known about the experiences anddifficulties faced by consumers after receiving a diagnosis of bipolardisorder, such as filtering and accessing reliable information, and thedegree to which these issues affect their ability and willingness to learn how to take control of their illness.**Method**A qualitative study was conducted which explored the experiences of peopleafter receiving a diagnosis of bipolar disorder, as communicated online toInformed Supporters (expert patients who have been managing theirbipolar disorder effectively for two years or more and are trained to provide an apomediary function to those newly-diagnosed with thecondition). The study was part of a wider randomised controlled trialevaluating an online psycho-education program. Supporter contact was limited to two contacts per week per participant. No constraints wereimposed on the number of contacts initiated by participants nor on thetype of issues raised. **Results**Twenty-six participants with recently-diagnosed bipolar disorder discussedissues and concerns online with Informed Supporters. Within theboundsof a serious mental illness, the nature of the interaction included guiding participants to information, collaborating, providing emotionalsupport and giving practical advice. Participants found the apomediaryfunction very helpful. Issues raised included medication side-effects,coping with symptoms, reaction to diagnosis, identifying early warningsigns and triggers, loss of sense of self, uncertainty about future andstigma. **Discussion** Consumers are often reluctant to discuss issues of a personal nature withtheir health professionals, yet these issues, if left unresolved, canundermine take-up of effective treatments or lead to prematurediscontinuation, which, in turn, can result in worsening symptoms and reduced quality of life. Expert patients offer empathy, real-lifeexperience, credibility and practical advice. In addition to helping folkwith newly-diagnosed bipolar disorder navigate through the onslaught ofinformation about the condition, apomediaries in this context can help toreduce feelings of isolation that often accompanies diagnosis and canfacilitate self-management and control of the condition.

Toomas Timpka, Department of Computer Science, Linköping University, Sweden; Johnny Ludvigsson, Department of Pediatrics, Linköping University, Sweden; Henrik Eriksson, Department of Computer Science, Linköping University, Sweden; Joakim Ekberg, Department of Computer Science, Linköping University, Sweden; Lena Hanberger, Department of Pediatrics, Linköping University, Sweden; Sam Nordfeldt, Department of Pediatrics, Linköping University, Sweden

Web 2.0 systems supporting childhood chronic disease management: a general architecture compliant with the WHA eHealth Resolution

(Research)

Introduction: At the time they reach adolescence, 10-15% of all children live with a chronic disease. The recent technical advances have provided a foundation for ubiquitous and proactive health systems that use data from multiple sources to supply individuals and communities with support to improve their state of health and avoid health risks The aim of this research was to develop a general Web 2.0 system architecture compliant with the WHA eHealth resolution for support of children with a chronic disease.**Methods:** Participatory action research was used to stepwise define a design specification in the form of a pattern language. Support for children diagnosed with diabetes type 1 was used as the example area. Each individual design pattern was represented graphically and textually in the form Title, Context, Problem, Solution, Examples and References. Application references were included at the lowest level in the graphical overview in the pattern language but not specified in detail in the textual descriptions. The design represented in the pattern language was progressively implemented in a Web 2.0 system during the development process. The resulting system was introduced for patients, relatives, and caregivers in 2006.**Results:** The resulting design patterns describe a Web 2.0 architecture supplying three main services to communities including children suffering from a chronic disease:

- access to resources for development and maintenance of the specific competences needed for management of the chronic disease in the community;
- endorsement of learning about the chronic disease management through online peer-to-peer communication;
- systematic accreditation of learning materials and processes.

The design patterns constituting the architecture are divided into functional and non-functional design elements, and formulated at the levels of organizational, system, and application design. Two application-level preconditions were identified for implementation of the architecture in specific communities. First, the implementations have to be epidemiologically sensitive in order to support provision of age- and need-adjusted delivery of services on an equal basis to both

resourceful and vulnerable groups of children. This means that the applications that constitute the interface between the system content and the users must be chosen to suit the information infrastructure in the community and be affordable for the broad majority of patients and their families. Second, to provide individualized support to children with a chronic disease, the system has to be accessible at all times and in all types of daily-life situations. Consequently, the applications cannot be restricted to stationary computer platforms, but also need to be available at different types of mobile devices, e.g. personal media players and cell phones. **Conclusions:** Even though a high level of user participation in developing and managing content is the core of the Web 2.0 concept, the WHA ehealth resolution necessitates to structure this participation and to assure the medical quality of the services that are provided. The use of design patterns allowed representing the core design elements of a Web 2.0 system architecture upon which an 'ecological' development of content respecting these constraints can be built.

Sara Urowitz, Oncology Education, Princess Margaret Hospital, University Health Netowork, Toronto, Canada

From Social Networks to Social Medicine: Exploring the role of online interventions

(Research)

Background: The Canadian healthcare system supports an universal publicly funded health insurance system that insures the costs of medically necessary services without co-pays to all legal residents of Canada, including permanent residents [1]. To ensure sustainability of this system, innovative cost effective and timely ways for delivering insured services are necessary. By pushing the boundaries of clinical practice, Web 2.0 technologies, including the proliferation of online communities [2, 3], provide possible alternatives for the provision of care to a nation. Caring Voices (www.CaringVoices.ca), a national platform for providing support and clinical education to cancer survivors is one such example and is one of the few online communities directly associated with an hospital. Caring Voices offers barrier free access to health care professionals for all Canadians, and offers both professional and peer-to-peer support and clinical education. **Objective**: To describe the creation of an hospital based social networking community, and to explore its feasibility for the delivery of clinical education and supportive care, and for conducting clinical research within the context of the Canadian health care system. **Materials:** www.CaringVoices.ca supports cancer survivors through asynchronous communication using forums, and synchronous text-based communication using a series of scheduled monthly events. Events provide clinical education and supportive care, and are moderated by either peer survivors, members of community cancer agencies, or health care professionals, including physicians, nurses, social workers and allied health care professionals. **Methods:** Formal usability testing was conducted with eight participants using open-ended scenarios and participant observations. Informal usability data have been collected since launch of the platform in 2006, and two feasibility studies are being conducted to explore the possibilities of using the platform for clinical research. **Results:** A total of 1180 users registered in the first 18 months with an average of 49.5 users registering per month. On average there are 9 monthly synchronous events with clinician led events account for 35% of synchronous communications. Other events included peer led support chats (41%) and living with cancer chats led by other experts in the cancer care community (24%). For clinically focused events moderators have been recruited from medicine (n=2), nursing (n=3), allied health (n=4) including social work, dietician, physical therapist and clinical psychologist, and other professionals (n=2) including a medical librarian and a personal trainer. Collaborations have been established with 3 hospital based cancer programs, 4 community based cancer programs, and both formal and informal training sessions have been provided for online moderators.**Conclusions:** Hospital based social networking communities provide a new and innovative way of meeting patients' needs for clinical education and support in a system that is committed to the provision of care but has limited resources. In addition to meeting patients' needs this approach may result in cost savings, patient empowerment and activation and is a way of achieving patient centred care. Providers' willingness to participate in alternate models of care delivery will dictate the success of these communities. Wide scale adoption of these innovations will have an impact on care delivery and how research is conducted.

References

1. Detsky, A.S. and C.D. Naylor, Canada\'s Health Care System - Reform Delayed. N Engl J Med, 2003. 349(8): p. 7. [Medline] PMID: 12930935

2. Schweizer, K.J., H. Krcmar, and J.M. Leimeister. The role of virtual communities for the social network of cancer patients. in Twelfth Americas Conference on Information Systems. August 4-6, 2006. Acapulco, Mexico. URL: http://www.winfobase.de/lehrstuhl/publikat.nsf/intern01/8A67987A14BBA4A9C125721200474D08/$FILE/06-33.pdf WebCite®: http://www.webcitation.org/5Xq9c8fw2 [cached 2008-05-15)

3. Eysenbach, G., et al., Health related virtual communities and electronic support groups. BMJ, 2004. 328(7449). [Medline] PMID: 15142921

1:30 PM - 2:30 PM in MaRS CR2

Chair: Margaret Hansen

Topic: Digital Learning

Leanne Bowler, School of Information Sciences, University of Pittsburgh

Where can teens find health information? A survey of web portals designed for teen health information seekers

(Practice)

The Web is an important source for health information for most teens with access to the Web (Gray et al, 2005a; Kaiser, 2001). While teens are likely to turn to the Web for health information, research has indicated that their skills in locating, evaluating and using health information are weak (Hansen et al, 2003; Skinner et al, 2003, Gray et al, 2005b). This behaviour suggests that the targeted approach to finding health information that is offered by web portals would be useful to teens.

A web portal is the entry point for information on the Web. It is the front end, and often the filter, that users must pass through in order to link to actual content. Unlike general search engines such as Google, content that is linked to a portal has usually been pre-selected and even created by the organization that hosts the portal, assuring some level of quality control. The underlying architecture of the portal is structured and thus offers an organized approach to exploring a specific health topic.

This paper reports on an environmental scan of the Web, the purpose of which was to identify and describe portals to general health information, in English and French, designed specifically for teens. It answers two key questions. First of all, what portals exist? And secondly, what are their characteristics? The portals were analyzed through the lens of four attributes: Usability, interactivity, reliability and findability. Usability is a term that incorporates concepts of navigation, layout and design, clarity of concept and purpose, underlying architecture, in-site assistance and, for web content with text, readability. Interactivity relates to the type of interactions and level of engagement required by the user to access health information on a portal. Interaction can come in the form of a game, a quiz, a creative experience, or a communication tool such as an instant messaging board, a forum or blog. Reliability reflects the traditional values of accuracy, currency, credibility and bias, and in the web-based world, durabililty. Findability is simply the ease with which a portal can be discovered by a searcher using the search engine that is most commonly associated with the Web by young people – Google – and using terms related to teen health. Findability is an important consideration since the majority of teens begin their search for health information using search engines (CIBER, 2008; Hansen et al, 2003). The content linked to by the portals was not evaluated, nor was the portals' efficacy as a health intervention.

Teens looking for health information on the Web in English have a wide range of choices available but French-language portals are much rarer and harder to find. A majority of the portals found and reviewed originated from hospitals, associations specializing in a particular disease, and governmental agencies, suggesting that portals for teens on health related topics are generally reliable. However, only a handful of the portals reviewed were easy to find, suggesting that valuable resources for teens remain buried in the Web.

Attendees will be provided with a comprehensive list of portals to general health information, in English and French, designed specifically for teens.

Margaret Hansen, School of Nursing, University of San Francisco, USA

Versatile, Immersive, Creative and Dynamic Virtual 3-D Healthcare Learning

(Research)

The author provides a critical overview of three-dimensional (3-D) virtual worlds (VWs) and "serious gaming" that are currently being developed and used in healthcare professional education and medicine. The relevance of this e-learning innovation for teaching students and professionals is debatable and variables influencing adoption, such as increased knowledge, self-directed learning, and peer collaboration, by academics, healthcare professionals, and business executives are examined while looking at various Web 2.0/3.0 applications. There is a need for more empirical research in order to unearth the pedagogical outcomes and advantages associated with this e-learning technology. A brief description of Roger's Diffusion of Innovations Theory and Siemens' Connectivism theory for today's learners is presented as potential underlying pedagogical tenets to support the use of virtual 3-D learning environments in higher education and healthcare

1:30 PM - 3:00 PM in MaRS CR3

Chair: Peter Murray

Handy Amin, Active Health Management, Health Promotion Board, Singapore; Andy Tan, Active Health Management, Health Promotion Board, Singapore; Ping Ping Tan, Chief Information Officer's Office, Health Promotion Board, Singapore; Sau Chan Low, Chief Information Officer's Office, Health Promotion Board, Singapore; Shyamala Thilagaratnam, Active Health Management, Health Promotion Board, Singapore

Planning the Development of the Singapore National Health Portal

(Practice)

Background: Singapore is an island city-state in Southeast Asia with a land area of approximately 700 km2 and a multi-ethnic resident population of about 3.6 million. A vibrant global city and a thriving centre of

commerce and industry, Singapore's competitive modern economy enjoys high economic growth, high literacy rates, and life expectancy comparable to other high-income countries.

Based on the National Health Survey in 2004, an estimated 1 million Singaporeans suffer from at least one of the four major chronic diseases, namely diabetes mellitus, hypertension, lipid disorders, and stroke. To address the growing burden from these chronic diseases, the Singapore government embarked on a national strategy to promote the concept of Active Health Management (AHM) to all Singaporeans. This strategy focuses on inculcating individual responsibility towards one's health condition through raising awareness and providing patient empowerment among the population.

The Health Promotion Board (HPB) is the government agency responsible for driving health promotion initiatives to improve the health and fitness of Singaporeans and to empower Singaporeans to attain optimal health throughout life. As part of the AHM strategy, HPB was tasked with developing the National Health Portal (NHP) – a personal health information portal enhanced with tailored empowerment tools for behavioural change.

Vision: The vision for the NHP is a one-stop web portal that empowers Singaporeans and their care-givers with their personal health and medical information and provides preventive and monitoring tools for personalised health management, so as to achieve better health outcomes.

Conceptualising the NHP:The NHP Taskforce, consisting of stakeholder representatives, was formed to drive the development of the NHP. To understand the landscape for health portals, a feasibility study was commissioned. Phase I involved a global review of health portals; Phase II involved dialogues with local stakeholder representatives to assess their readiness and gather their inputs for the NHP. Plans for the implementation of the National Health Portal were developed based on the study findings.

Implementation Plans for the NHP:The NHP aims to empower users by providing access to their personal health information. This includes self-entered data (e.g. user profile, Health Risk Assessment) with their Electronic Medical Records (EMR) obtained from healthcare providers. Based on the user's personal health information, tailored health information, relevant Behavioural Health Intervention Programmes (BHIP), and supporting health management tools will be provided. The implementation of the NHP will be conducted over 3 distinct phases from 2008 to 2011. The initial phase will focus on self-entered data of at-risk individuals and chronic patients from the public health sector, and include three BHIPs (weight management, smoking cessation, and diabetes management). Integration with EMRs from public sector healthcare providers, interfacing with the private healthcare sector medical records, and the inclusion of additional BHIPs will be implemented in subsequent phases.

Conclusion: The experience of planning the development of the Singapore NHP will be valuable for other countries that are considering a similar strategy to support AHM within the population.

Jason Aprile, OntarioMD, Toronto, Canada

Providing Online Access to Point-of-Care Information: Lessons from the OntarioMD.ca Physician Portal

(Business)

OntarioMD.ca (www.ontariomd.ca) is a Web portal which provides clinicians access to a variety of information resources. The content delivered via the portal is organized and presented according to lessons gleaned through the ongoing engagement of portal users and other stakeholders.

Recent studies regarding the information needs of clinicians (Davies (2007) and Perley et al. (2007), have concluded that online content must meet certain criteria in order to be effective. These findings include the need for content to be: available at the point of care, easily searchable, customizable and anticipate the user's needs.

Since 2006, OntarioMD.ca has been developing resources which address these criteria. This presentation will demonstrate how OntarioMD.ca engaged in the development of resources and best practices which address the clinician's need for reputable point of care information.

References

Davies K. The information-seeking behaviour of doctors: a review of the evidence. Health Information and Libraries Journal, 25, 2007.OntarioMD Web portal. URL: http: //www.ontariomd.ca

Perly CM, Gentry CA, Fleming AS, Sens KM. Conducting a user-centered information needs assessment: the Via Christi Libraries' experience. J Med Libr Assoc 95(2) April 2007.

Grant Cumming, NHS Grampian Scotland; University of Aberdeen; Andrew Fowlie , NHS Grampian; Adrian Baker , NHS Highland; Thomas Heneghan, NHS Grampian; Neil Hamilton , University of Aberdeen; Stefan Agamanolis

e-health in a Remote and Rural Setting in the North of Scotland- An Evolving Success Story

(Practice)

Thomas Friedman's aphorism Connect and Collaborate from his book "The world is Flat together with the enthusiasm supported by scientific rigor generated from Mednet 2006 were the catalysts for developing an infrastructure to delivering health and social care differently to a population of 120,000 and a geographical area covering 2238 squarekm^2 in the North of Scotland.

On the back of seed funding for economic growth in the region and the success of menopause1 and miscarriage2 websites, www.babyfeedingmattters.co.uk was commissioned. NHS Grampian then made funding available to develop the use of the website with a teleconferencing facility to promote breastfeeding and support of women in a rural setting. NHS Grampian then provided further funding for the development of www.health-e-space.com – both as a conduit and an

infrastructure to deliver holistic health care to the Moray area of Scotland under the self care agenda. This infrastructure is clinically driven and will allow in addition to providing information, support forums, story telling and relevant links to kite marked sites, management of chronic conditions where pathways of care are clear and management of complex care where multiple conditions/social circumstances are impacting on health and wellbeing. The website is being developed using the PEDI process (Personalized predictive health care plans, Evidence based health and care, Digital consultations with health care professionals and Interactive web-based care). Health-e-space.com will be subdivided into community health-e-space, spiritual health-e-space and personalized health-e-space. The user will be able to have access to their own health content using the tools on www.health-e-space.com where they can bring together quality assured information about their own health, anticipate changes and create their own record and risk assessment on their own computer using predictive modeling. If they have a 10% or greater risk of being admitted to hospital in the next year, an interactive anticipatory care package will be designed (if appropriate) for their use to reduce this risk.

At all stages, site development will be subject to focus group scrutiny and all aspects of delivery of the service will be subject to qualitative and quantitative analysis.

Because of its rurality Moray has several isolated communities, Dufftown is one such community-not only is it the malt whiskey capital of the world but it will be the first to pilot www.health-e-space.com and self blood pressure monitoring with interactive consultation with a clinician if required. A pilot using a cohort of 50 people is in progress.

www.health-e-space.com is unique not only in its collaboration with universities, clinicians and industry but also in generation of revenue streams. Generating revenue streams other than non recurring funding is the challenge of websites in general and medical-related websites in particular. We believe we have developed a unique symbiotic relationship with the local newspaper by creating links to their website and advertising streams.

An overview and update of ehealth in Moray will be given.

References:

1. Cumming GP, Herald J, Moncur R, Currie H, Lee AJ. Women's attitudes to hormone replacement therapy, alternative treatments and sexual health. Menopause International 2007;13:79-83

2. Cumming G, Klein S, Bolsover D, Alexander D, Hamilton N. The development and evaluation of a miscarriage-related website for women and partners following early pregnancy loss : A pilot study11th World Congress on Internet in Medicine 2006: 26

Jaime Jiménez Pernett, Andalusian School of Public Health, Granada, Spain; Jose Francisco García Gutiérrez, Andalusian School of Public Health, Granada, Spain; Pablo Martínez Cabrero, Andalusian School of Public Health, Granada, Spain; Sergio Minué Lorenzo, Andalusian School of Public Health, Granada, Spain

Knowledge Exchange Nodes in Public Health: Two successful practical experiences

(Practice)

The importance of the traditional core public health areas of epidemiology, biostatistics, environmental health, health services administration, and social and behavioural sciences is still paramount. However, public health professionals will be better prepared for the future if they achieve competency in other eight areas: informatics, genomics, communication, cultural competence, community-based participatory research, global health, policy and law, and public health ethics. For many Schools of Public Health (SPH) around the globe such new framework is certainly overwhelming.

On "the age of the web 2.0.", SPH should foster new forms of scientific and educational collaboration, and must actively develop innovative ways of community-based research, learning, and service.

Teaching, Research and Knowledge Exchange Nodes -that connect the work of different SPH by using the Internet and Web 2.0 tools- may be a valuable option.

Description of the experience:In this context, we would like to present two innovative experiences in which the Andalusian School of Public Health (ASPH) is participating.

> Virtual Campus of Public Health. A web-based initiative sponsored and coordinated by the Pan American Health Organization (PAHO) with the collaboration of six SPH (INFOMED-Cuba, FIOCRUZ-Brasil, ASPH-Spain, National SPH-Chile, Cayetano Heredia-Peru, Antioquía-Colombia). A network of Knowledge Exchange Nodes (KEN) that maintains a central node through RSS feeds. It is designed to share courses, learning objects, educational materials, research projects, publications and information resources under Creative Commons (CC) and Open Educational Resources (OER) licences.

> Best practices in Public Health teaching and learning. A pilot content management system (CMS) designed by the ASPH to promote and facilitate the exchange of educational initiatives. It includes a "best-practice bank" for successful practical experiences in teaching public health, and an online searchable database with more than 200 critically-appraised documents about learning methodologies for health sciences. There is also a report on "evidence-based teaching for public health" and a "benchmarking forum". This web-site will serve as a common best-practice platform for all the institutions mentioned above, and it will be open to any other organization interested in collaborating.

Conclusions and discussion:To address the complex challenges of the 21st century and make additional improvements in the health of the public depends, in large part, upon the relevance and quality of public

health education and training worldwide. SPH need to create different forms of relationship using an imaginative expansion of a global collaborative culture

Knowledge Exchange Nodes & Networks will help realize an unprecedented vision of public health that support free sharing of applied innovation and dissemination and facilitate the exchange and uptake of "new knowledge using new technologies".

3:30 PM - 5:00 PM in MaRS Auditorium

Chair: Judy Proudfoot

Topic: Building virtual communities and social networking applications for patients and consumers

Dorrit Billman, Stanford CSLI and Palo Alto Research Corp; Diane Schiano , Palo Alto Research Corp; Lee Gugerty, Clemson, Psychology

Patient Problem-Solving on the Web: How do Patients Use Web Forums to Cope with Chronic Disease?

(Research)

Introduction: Patients' use of the web, particularly patient-to-patient (e.g., online fora) ,is playing an influential and transformative role in health care, particularly around chronic diseases (Fox07). We need to understand the specific types of work that patients attempt in online fora and the barriers or facilitators to their goals. This can guide design of new web health technologies, across the varied types of sites, tools, and activities. We are studying naturally occurring material (Coulson,Buchanan,&Aubeeluck07) postings on health fora about several chronic diseases.**Method:** We have collected a large amount of material from public forums on several chronic diseases. We have selected fora and within fora sampled threads over time with additional inclusion of long threads. Our initial analysis focuses on the first message of each thread, to identify the issues patients raise and how exchanges are initiated. We have developed coding to capture variation and patterns suggested by extensive reading of the material. In our talk we will present qualitative and quantitative findings. **Results:**We will report on three of the first-message codes we developed, for Form, Topic and Schema, and summary results. Form was coded as Ask (for information or action), Tell (personal or general information), and Other (including Greetings and Jokes). Topic was coded as Physiological (treatments, mechanisms), Pragmatic (providers, insurance), and Social/Emotional (comfort, sympathy, validation). Neither Form nor Topic codes have mutually exclusive values: For example, the primary form of a message might be ASKing for information, but include TELLing; or multiple topics might be addressed. In our initial sample of 100 threads, 60% of first messages concerned Physiological, 40% were Social/Emotional, 40% were pragmatic, and few percent were other. 33% of the messages were multi-topic.We looked for frequently recurring message 'schemas', conventional structures of the message as a whole. 1) In the Problem&Question schema, the user asks a question about a problem (typically, own health) but also tells about their condition as background; 1/4 of the messages fit this schema. 2) In Give-Information, the user simply posts information judged valuable to the community or an individual; 1/8 of the messages fit this schema. Content may be expert-authored or come from personal experience. **Discussion:**Overall, a wide range of difficult activities were addressed in first-messages. On the cognitive side these included reasoning about causes and treatment effectiveness, responding to or planning for visits to providers, soliciting help in making a decision, and gaining initial understanding of an unfamiliar condition, treatment, or test. On the emotional side, these included asking for help facing a frightening event, venting, validating experience, and providing specific encouragement or concern. Message coherence and complexity varied greatly.We will report qualitative and quantitative analysis of first-messages on user forums for chronic disease, characterizing the types of problems that users try to solve in online communities. Our next step is to look at the problem solving outcomes and methods of the community's response across a thread. We also plan to compare this to other web forms of patient-to-patient interaction.

Neil Coulson, University of Nottingham

Living with HIV/AIDS and use of online support groups

(Research)

Introduction: A growing number of individuals are searching for health-related information, advice and support on the Internet [1,2] including that which is related to HIV/AIDS [3-5]. Qualitative studies [4,5] suggest that for individuals living with HIV/AIDS, the Internet has helped individuals to connect with each other, often through participation in online support groups. The present study aimed to explore the association between use of online support groups by individuals living with HIV/AIDS and medical and health status, coping and social support. Methods: In order to undertake this research, 8 HIV/AIDS-related online support groups were identified from an Internet search engine (i.e. google) and their moderators were contacted and invited to participate in the study. A positive response was received from one website (http://www.aidsmeds.com) where permission to recruit participants was granted. A recruitment message, together with a link to our study website, was emailed to those individuals who had subscribed to their mailing list as well as being posted on their bulletin board. Inclusion criteria of the study were individuals who were aged 18 or above, and had been HIV-positive for at least 1 month. Participants were directed to the online questionnaire, and online consent was sought with recruitment taking place over a 4-week period. **Results:** A total of 640 respondents living with HIV/AIDS completed our online questionnaire. The majority were male (82.9%) with a mean age of 45.52 (age range = 19-73 years). Approximately 85% were college educated or higher and approximately half were single.

The majority of respondents were from North America (80.3%), followed by Europe (8.8%), Africa (6.9%), Asia (2.5%) and Australia (1.4%). The average time since diagnosis was 9.7 years (range <1 to 18 years) with 62.3% reporting being asymptomatic, 12.7% symptomatic and 25% with AIDS. Using a median split, respondents were classified into one of three groups reflecting the time spent accessing online support groups in the last month. (1) 'non-users' comprised those individuals who reported that they had not used such (41%); (2) 'infrequent users' were those who reported accessing online support groups up to 4 hours (26.4%) and (3) 'frequent users' were those who had accessed online support groups for more than 4 hours (32.3%) in the past month. Our analysis revealed that frequent users were more likely to be female, younger, single, in a more advanced disease stage, and reported spending more time accessing other HIV/AIDS-related websites. In addition, both infrequent and frequent users reported being more recently diagnosed than non-users. After controlling for these factors, our multivariate analysis revealed that frequent online support group users reported greater limitations due to physical functioning, social functioning and poorer general health than non-users. In addition, both frequent and infrequent online users scored higher in planning, active coping, instrumental support and emotional support coping. No significant difference was found for social support. Discussion: Our results suggest that online support groups may potentially offer some benefits for those living with HIV/AIDS though more longitudinal research is needed. Implications for healthcare and future directions will be considered.

1. Madara EJ. The Mutual-Aid Self-Help Online Revolution. Social Policy 2007; 27(3): 20-26.

2. Powell J, Clarke A. The WWW of the World Wide Web: Who, What, and Why? J Med Internet Res 2002; 4(1): e4.

3. Flicker S, Goldberg E, Read S, Veinot T, McClelland A, Saulnier P, et al. HIV-Positive Youth's Perspectives on the Internet and eHealth. J Med Internet Res 2004; 6(3): e32.

4. Reeves PM. Coping in Cyberspace: The Impact of Internet Use on the Ability of HIV-Positive Individuals to Deal with Their Illness. J Health Commun 2000; 5(1): 47-59.

5. Reeves PM. How Individuals Coping with HIV/AIDS Use The Internet. Health Educ Res 2001; 16(6): 709-719.

Trevor Van Mierlo, No University Affilliation, V-CC Systems Inc. (Toronto, Canada) & V-CC Systems USA Inc. (San Francisco, USA); Peter Selby Mbbs, Ccfp, Centre for Addiction and Mental Health, University of Toronto, Ontario Tobacco Research Unit; Danielle Parent (rn), None (V-CC Systems Inc);John Cunningham, Phd, Centre for Addition and Mental Health, University of Toronto

Moderated online social networking for smokers: Content of first posts from 2,562 members

(General)

Objective: Social support and group therapy is associated with successful smoking cessation. However, many smokers simply do not have access to, or time to participate in, real-world smoking cessation clinics or support groups. Would the convenience or accessibility of online versions of social support facilitate connection between widely dispersed groups of quitters who may experience isolation from either a geographic or temporal perspective? Although increasingly popular, there has been little research or best practice guidelines in the general development, management, and sustainability of online social networks for behaviour change or Web Assisted Tobacco Interventions (WATIs) specifically.

Key learnings from over eight years of eHealth social network management will be fused with the results of a qualitative study illustrating theoretical aspects of online social support, common discussion themes, average response times for new users, and the characteristics of smokers who post in an online smoking cessation support group.**Methods:** Data were collected from StopSmokingCenter.net version 5.0, a WATI equipped with an online social support network moderated by trained Health Educators from November 6, 2004 to May 15, 2007. Demographics for both users and non-users of the online social support network were analyzed, and qualitative analyses were conducted to explore message themes and message content. The frequency of and patterns of posting were also analyzed.**Results:** 16,764 individuals registered, with 70% reporting being American. The mean age of registrants was 38.9 years and 65.4% reported being female. The mean number of cigarettes smoked was 20.6 per day. The mean score for 6,849 (41%) users who completed the Fagerstrom Test for Nicotine Dependence was 5.6. Of all registered members 15.3% (n=2,562) made at least one post in the Online Social Support network with 25% of first posts receiving a response within 12 minutes. The most frequent first posts were from recent quitters who were struggling with their quit attempts and most responses were from members who had quit for a month or more. Demographic characteristics between Support Network users (Posters) and registered users who did not post (Lurkers) were statistically but not clinically significant. **Conclusions:** Peer to peer responses to new users are rapid, indicating that online social support networks may be a particular benefit to recent quitters who experience difficultly in their cessation attempt. Accessing this kind of rapid support from a professional would take an

inordinate amount of time and money. The interactions appear consistent with in person social support groups. Further research in the effectiveness of WATIs with online social support networks is required to better understand the contribution of this feature to cessation. Also of importance will be determining if there is benefit for those who do not post but view the discussion of others.

A version of the data collected in this study was presented by Dr. Peter Selby at the Society for the Study of Addiction (SSA) 2007 Annual Symposium, York, United Kingdom, November, 2007.

An original paper utilizing data from this study has been submitted to the Journal of Medical Internet Research (JMIR) special issue for Web-Assisted Tobacco Interventions (WATIs)

Cornelia Van Uden-kraan, Institute for Behavioural Research, University of Twente, Enschede, The Netherlands; Constance Drossaert, Institute for Behavioural Research, University of Twente, Enschede, The Netherlands; Erik Taal, Institute for Behavioural Research, University of Twente, Enschede, The Netherlands; Erwin Seydel, Institute for Behavioural Research, University of Twente, Enschede, The Netherlands; Mart Van De Laar, Institute for Behavioural Research, University of Twente, Enschede, The Netherlands

Patient-initiated Online Support Groups: Motives for Initiation, Extent of Success and Success Factors

(Research)

Background Nowadays patients are able to choose between many online patient support groups [1, 2]. Most groups are initiated by individual patients or patient organizations. The threshold to start an online support group is low [3]. With patient-initiated groups now mushrooming, a need for an increase in research on this type of online support group is warranted [4]. **Objective** In a qualitative study, we explored the motives and goals of people who take the initiative to start such a group, so called webmasters. In addition, we wanted to learn more about how webmasters define success and success factors. **Methods** The webmasters of all Dutch online support groups for patients with arthritis, fibromyalgia and breast cancer (N=26) were approached if they were willing to be interviewed. In total 23 webmasters (7 arthritis, 8 fibromyalgia and 8 breast cancer) agreed to participate. The interviews were audio taped with prior consent of all participants and transcribed verbatim. Data were analyzed by two coders, using inductive analysis. Both coders separately read the entire transcripts several times, to identify emerging themes. Then the two coders met to discuss their findings in order to resolve differences. **Results** The analysis of the interviews revealed that most webmasters have altruistic motives for initiating a group, such as the provision of information and support or the empowerment of patients. However, several webmasters also mentioned intrinsic motives, like a hobby, or even as a kind of advertisement for a book. Webmasters defined success as the fulfillment of the goals they had in mind when they initiated their groups. Our study showed that before the support group goes online (the initiation stage), several decisions have to be taken about access, embedding, design, and content of the online group. The webmasters mentioned a number of pros and cons of the differing options, and stressed that the decisions need to be in coherence with their goals. When the group is online (the evolution stage), the success is dependent from the webmaster's success in managing the processes and people in the online group. Although webmasters have less influence over these aspects, since they do not control the participants of the online group, the webmasters mentioned pointers concerning the ways of promoting and financing the group, how to keep the group alive, the extent of moderation, the organization of meetings and rituals and the extent to which the wishes of the participants should be followed. **Conclusions** Our study yielded into guidelines for webmasters that might lead to successful online patient support groups. Success of the group is important, since it can be expected that effects of participation in an online support group for patients may vary, depending on the group's success.

References

[1] Meier A, Lyons EJ, Frydman G, Forlenza M, Rimer BK. How cancer survivors provide support on cancer-related Internet mailing lists. J Med Internet Res 2007 May 14;9(2):e12.

[2] Ginossar, T. Online participation: A content analysis of differences in utilization of two online cancer communities by men and women, patients and family members. Health Commun 2008;23: 1-12.

[3] Preece, J. Online Communities. Designing usability, supporting sociability. Chichester, UK: Wiley, 2000.

[4] Eysenbach G, Powell J, Englesakis M, Rizo C, Stern A. Health-related virtual communities and electronic support groups: systematic review of the effects of online peer-to-peer interactions. BMJ 2004; 328: 1166-1170.

3:30 PM - 5:00 PM in MaRS CR2

Chair: Benjamin Hughes

Topic: Wikis

Saulo Bortolon, Dept. Informatics, UFES, Vitoria, Brazil; Roberta Gomes , Dept. Informatics, UFES, Brazil; Mariella Berger, Hilario Seibel Jr., CEFETES; Serra, ES

Using Wiki for a Large Medical Nomenclature Translation

(General)

We report ongoing research on terminology and Open source software in Healthcare. We describe a collaborative environment for translating SNOMED-CT to portuguese. MediaWiki was used as the first implementation, but alternatives are being analyzed. Discussions about the pros and cons of adopting this solution in a nationwide effort to adopt international

standardized nomenclatures are presented. We discuss the importance of Terminologies for interoperability and its relations to Classifications, Ontologies and Archetypes.

The collaboration process needs a hierarchical structure, not the usual, democratic wikipedia approach. An account manager has to allow incoming translators to enter one or more medical subareas. Each subarea (eg. ophthalmology) has a senior participant with power to determine which is the best translation to portuguese, or which are the acceptable translations, for each SNOMED concept. This responsability may be transferred to some of the other participants by this senior one. The regular partipant has only the power to suggest new translations.

Derek Hansen, College of Information Studies, University of Maryland, College Park, USA; Sean Munson, School of Information, University of Michigan, Ann Arbor, USA

Patient-Driven Content Creation: Effectively Introducing Wiki Repositories to Virtual Support Communities

(Research)

Background: Virtual patient communities are an important source of information and social support for many patients (e.g., [1]). Most patient communities use a threaded conversation design such as an email list or discussion forum. This simple design supports highly personalized and empathetic communication [2-3]. In addition, many communities augment their conversations with community repositories (e.g., websites, FAQs) that distill their conversations, help newcomers find their bearings, and promote their community. Wiki platforms provide a new way of creating these community repositories collaboratively [4]. However, because wikis are new to most patient communities it is not clear how to effectively introduce and use them.**Objectiv**e: To identify challenges of collaborative authoring by a patient support group and effective strategies for overcoming those challenges. **Methods:** We performed an action research field study of 3 existing online patient groups. We provided each group with a customized wiki and suggestions on how to effectively use it. Suggestions were based on a prior study of a successful technical support community and tailored to the medical context [4]. Data from discussion archives, wiki page histories, and server logs were used to analyze participation patterns and wiki growth. Data from interviews and conversations between community members and the investigators were analyzed using a grounded theory approach, where common themes were identified and contrasted. The evaluation focused on both technical and social issues, similar to other studies implementing novel technologies (e.g., [5]) **Results:** The three groups varied significantly in the number of contributors and amount of content they created using the wikis, ranging from 1 contributor and a handful of pages (Pain Management) to a dozen contributors and 26 pages (Graves' Disease) to over 70 registered users and over 400 pages (GIST Cancer). Interviews and content analysis identified several challenges in introducing and sustaining the wiki repository. Social challenges included motivating contributions to the wiki, getting community buy-in, the hesitancy of members to edit other members contributions (even when those edits would be beneficial), and reconciling different frameworks for organizing the content. Several strategies helped overcome some of these challenges including: creating a Wiki Team that shared ownership of the wiki and having them seed the wiki with content before introducing it to the community; emphasizing the differences between a wiki and threaded conversation and how they can strengthen each other; and establishing realistic expectations about the temporal pace of activity on the wiki as compared to the discussion forum (i.e., intermittent and bursty wiki participation versus frequent and steady discussions). The most significant technical challenge was learning wiki editing syntax. Although none of the participants had prior wiki editing experience, they all learned to use the wiki with little coaching from the research team. **Conclusions:** The social challenges of implementing a wiki repository were far greater than the technical challenges, despite contributors' general lack of technical expertise. Further research should validate the successful strategies for overcoming these challenges that were identified in this study and develop novel social and technical strategies for creating patient-driven wiki repositories.

References:

[1] Meier A, Lyons EJ, Frydman G, Forlenza M, Rimer BK. How cancer survivors provide support on cancer-related Internet mailing lists. J Med Internet Res 2007;9(2):e12. http://www.jmir.org/2007/2/e12/

[2] Galegher J, Sproull L, Kiesler S. Legitimacy, authority, and community in electronic support groups. Written Communication 1998;(15): 493-530.

[3] Maloney-Krichmar D, Preece J. A multilevel analysis of sociability, usability, and community dynamics in an online health community. ACM Transactions on Computer-Human Interaction (TOCHI) 2005;12(2): 201-232.

[4] Hansen DL. Knowledge Sharing, Maintenance, and Use in Online Support Communities. Dissertation 2007. School of Information, University of Michigan. http://hdl.handle.net/2027.42/57608

[5] Leimeister JM, Krcmar H. Evaluation of a Systematic Design for a Virtual Patient Community. Journal of Computer-Mediated Communications 2005;10(4), article 6.

Aviv Shachak, Faculty of Information Studies; Health Policy, Management and Evaluation, University of Toronto; Jamie Meuser, Department of Family and Community Medicine, University of Toronto; Tupper Bean , Centre for Effective Practice, Department of Family and Community Medicine, University of Toronto; Stephen Hockama, Faculty of Information Studies, University of Toronto; Lena Salach, Centre for Effective Practice, Department of Family and Community Medicine, University of Toronto; Noah Ivers, Department of Family and Community Medicine, University of Toronto; Michael Evans , Department of Family and Community Medicine, University of Toronto

Collaborative Knowledge Translation: Application of a Wiki Model for Primary Care Practitioners

(Research)

Background: Current internet applications, collectively referred to as web 2.0, enable users to participate in generating and managing content and collaborate in the creation and dissemination of knowledge. Wikis are a particular type of web 2.0 applications, which consist of a set of interlinked web pages that can be easily constructed, edited or removed by users. The wiki model transcends traditional borders between researchers (or integrators of research evidence) and users, allowing both to contribute and engage in an on-going discussion. Therefore, wikis may provide an ideal platform for integrated knowledge translation. However, the potential of wikis and other web 2.0 applications for knowledge translation in health care is just beginning to be realized.

In 2004 the Department of Family and Community Medicine (DFCM) at the University of Toronto was engaged by Elsevier to undertake the review and revision work necessary to publish the fourth edition of its Family Practice Source Book. Over 194 faculty, residents, and students contributed to this effort. . This resource, representing a compilation of recent evidence collected from published research, covers current approaches and controversies in the prevention, diagnosis. and treatment of a wide variety of problems encountered in primary care. The book has been successful, selling over 7000 copies. It serves as a quick reference tool for family medicine residents and primary care providers.

The Centre for Effective Practice (CEP), an initiative within the DFCM, has as its primary goal to address the growing gap between best evidence and current primary care practice, and to produce practical solutions to address these barriers. As part of its mission the CEP has engaged in negotiations with the publisher, Elsevier, to purchase the copyright of the Family Practice Source Book, and to deliver an online, updated version, based in part on a wiki. This project represents an attempt to leverage web 2.0 tools to facilitate knowledge translation by directly connecting researchers and health care providers via the emerging online community. It transcends traditional roles by allowing both researchers and practitioners to participate in the synthesis of evidence and to engage in an on-going discussion. Crucially, this wiki will be updated and available continuously, thereby fostering the faster creation and dissemination of knowledge.**Objectives:** The main objective of this project is to develop and investigate the feasibility of offering a leading textbook of family medicine via a wiki environment to primary care professionals across Ontario. We will accompany the project with a research component to evaluate users' perceptions of the wiki sourcebook, and its contribution to knowledge translation and practice enhancement.**Methods:**The content of the Family Practice Sourcebook will be transformed into a wiki environment. The wiki will be opened to all primary care providers in Ontario. Mixed methods will be used to collect and analyze data on how best practices resources are created and evolve collaboratively; contributors and users' perceptions of the wiki's usefulness, ease of use, credibility and effect on behavior; and its diffusion within the primary care community. Specific methods include content and discourse analysis, key informant interviews and surveys.

3:30 PM - 5:00 PM in MaRS CR3

Chair: Chris Paton

Topic: Web2.0-based medical education and learning

Panagiotis Bamidis, Medical School, Aristotle University of Thessaloniki, Thessaloniki, Greece; Stathis Constantinidis , Medical School, Aristotle University of Thessaloniki, Thessaloniki, Greece; Eleni Kaldoudi , Medical School, Democritus University of Thrace, Alexandroupolis, Greece; Nicos Maglaveras , Medical School, Aristotle University of Thessaloniki, Thessaloniki, Greece; Costas Pappas, Medical School, Aristotle University of Thessaloniki, Thessaloniki, Greece

The use of Web 2.0 in teaching Medical Informatics to postgraduate medical students: first experiences

(Research)

IntroductionRecent technological advances have shifted the core focus of medical education towards more active learning approaches. This shift is certainly related with evolutions on the Internet and the Web, and certainly the emergence of Web 2.0. The latter is being stressed as a promising tool for advanced support of medicine and medical education. Although Web 2.0 emphasizes on participation, in its early days is still used in the majority of cases to hold and provide content. In this paper, we demonstrate how we used Web 2.0 tools like wikis, blogs and discussion forums in delivering specific topics of Medical Informatics to postgraduate medical students. **Methodology**The approach is based on a blended learning scheme, where e-learning is actually complimentary to traditional classes (lectures, demos and labs). The approach utilises the SCORM standard within an open source

learning environment (Moodle). Emphasis is placed not only in creating and promoting information, but also how to best utilise Web 2.0 as an active support mechanism towards a problem (or case) based learning. In our approach, students and instructors use the web as a virtual place to collaborate and create new knowledge and new educational experiences. **Evaluation**Students' evaluation was asked in two stages. In the first stage, students were asked to complete an anonymous questionnaire emphasising merely on the use of Web 2.0 tools. The Moodle system lead the logged-in students to an external web site powered by another open source source survey tool, namely, "PhP Surveyor", so as to maintain anonymity.

Evaluation of the results indicates that students were quite satisfied by this approach, and were happy with the collaboration opportunities offered to them. Most of them admitted that they were tempted to "read what others have contributed in their own personal blogs" (which by the way were given public access to the enrolled students). In addition, they believed that they found the student collaboration opportunities offered by the system tools quite useful and enhancing the overall learning process.

In the second stage students were asked to evaluate the whole course using a dedicated, home made, evaluation software, specifically developed for course evaluation purposes within the postgraduate program of the Medical School at AUTH. These evaluation results demonstrate a significant course improvement of the last year quality when compared with those obtained in the past 5 years. **Discussion**Evaluation of results indicates that Web 2.0 technologies have a major role to play within the educational arena. Although we have applied them to a physically akin to them course, i.e. the medical informatics course, the methodology we have utilised is by no means exclusive to other courses. In the contrary, we believe the approach is directly applicable, and we intend to expand this effort to a number of medical curriculum courses over the next few years.

Deirdre Bonnycastle, College of Medicine, University of Saskatchewan

Medicine 2.0 and Medical Faculty Development

(Practice)

The University of Saskatchewan, like many Canadian Universities, has 800 part-time medical faculty members scattered throughout the province, many of them working in small communities with limited opportunities to attend faculty development workshops in urban locations. Having faculty development personnel travel to remote locations to conduct workshops 1-1 is economically challenging and may not meet the needs for collegial interaction.

As Saskatchewan moves towards a more distributed medical education model, new pedagogies and technology become essential topics for faculty awareness.

In the past 5 years, there has been a rapid increase in online technology that allows dispersed communities to interact with one another. Web 2.0 refers to online tools such as blogs, wikis, social networks and virtual simulations that allow participants to involve themselves in learning and connecting to others at a distance.

Combine these tools with the need for faculty development and the University of Saskatchewan sees an obvious fit both in the ability to offer learning opportunities and to role model how to use Web 2.0 technology with medical students.

Deirdre Bonnycastle, the Clinical Teaching Development Coordinator for the College of Medicine will discuss the successes and failures of the Web 2.0 tools such as Elluminate, Moodle, blogs, wikis, podcasts, Second Life, Facebook, Ning and TeacherTube that she has used as a faculty development tool.

Bertalan Mesko, University of Debrecen, Debrecen, Hungary

Medical education and building an on-line reputation in the world of web 2.0

(Practice)

The new websites, services and tools of web 2.0 can play an important role in the future of medical education. These web tools, expert-based community sites, medical blogs and wikis can facilitate the work of physicians, scientists, medical students or medical librarians. The new generation of web services can change the way medicine is practised and healthcare is delivered.

In the new, web-based world, building an on-line reputation is becoming crucial for medical professionals. Patients are more likely to search for information about their doctors on the web, and a forum entry should not be the only resource about a medical practice. That is why physicians should take control of the dissemination of medical information in order to represent properly their own practices on the web. Building an on-line reputation is a matter of hard work, effort and good marketing. Writing medical blogs, participating in medical wikis or being a member of medical communities can improve the search results of advertising your medical practice or research.

Regarding medical education, medical blogs provide content and express opinion on healthcare that you can never find in a medical paper. More and more physicians and medical students maintain their own blogs nowadays as it is a good method for making new contacts with people all over the world and is an excellent tool for career building.

Second Life is an online 3-D virtual world with more than 13 million residents as of April, 2008. This virtual world makes it easier to communicate with people; to educate people without boundaries; to use videos, images, texts, web links, online presentations, tutorials and e-learning tools at the same time. The future of medical education is being constructed at the Ann Myers Medical Center, which was established in 2006 to test the possibilities of training medical and nurse students in a virtual medical center. The AMMC organizes medical exercises which are free and open to any kind of medical professionals. Educators present

medical cases, upload medical images which medical students have to discuss and find out the proper diagnosis. There are some other educational opportunities in Second Life such as the Heart Murmur Sim where you can listen to cardiac murmurs. Second Life offers numerous educational opportunities for physicians and medical students as well.

A medical professional does not just help fight quackery and medical misinformation by taking an active part in the medical communities and databases of web, but he can also create his own on-line reputation. It is vital that medical professionals take control of publishing medical information on the web. And by using these tools to ensure the quality of medical articles and blogs on the web, medical professionals can also set their online presence.

My aim is to present all the features of web 2.0 in the fields of medical education and healthcare through the story of my medical blogs which have been visited for more than a million times in one year's time and have won several blog awards.

Rod Ward, University of the West of England, Bristol, UK

The potential and challenges of Web 2.0 in the education of healthcare professionals.

(General)

Web 2.0 technologies have the potential to change the education of healthcare professionals, at undergraduate and postgraduate levels, from a didactic one way process, in which information is transferred from the "expert" to the student, to a collaborative and participative process, empowering the student to be an equal participant in the learning process. However there are significant challenges and hurdles which need to be considered.

The potential for elearning to enable and empower healthcare students within educational programmes has been discussed for many years, [1] however this has been challenged with calls for moves from instructivist to constructivist learning approaches, built around "Communities of Practice", which potentially provide the greatest scope for learning through interaction and discussion [2].

Web 2.0 technologies are emerging as platforms to enable or encourage students to be collaboratively creating and sharing their own insights into current and emerging themes within their education. This "architecture of participation" has been described as "emphasising the pre-eminence of content creation over content consumption", [3] and the use of Web 2.0 applications as "mind tools to stimulate reflection and actively involve learners in their own construction of knowledge" which have been proposed as a way to yield powerful learning experiences [4]. It may also be important for healthcare professionals to be aware of the emerging technologies, and their potential development in the future as their patients and clients may well be using them.

Where software such as blogs, wikis, podcasts and social networking tools are provided by formal education providers they can by made public or restricted to particular groups of students and are often "moderated" by a member of staff. Many students are taking greater control and setting up their own mechanisms, sometimes via platforms such as Facebook, for both structured and "just in time" learning, to enable them to collaborate, and academic staff may be explicitly excluded by the students from these applications.

These developments bring challenges to existing structures and power relationships, in which academic staff or experts have control over the learning process, and may shift the power towards the students hands, either individually or in groups. This may be challenging for many, both academics and students, and will require different perceptions of role, and possibly demand a rethinking of existing pedagogy.

The emergence of new forms of knowledge generation and distribution such as Wikipedia, brings issues with the assessment of the reliability or accuracy of resources [5] and may challenge the role of academic journals, which are already having to change their production and financial models in the light of open access and eprints initiatives. Additional issues arise in the detection of plagiarism and the identification of clear mechanisms for assessment of group work in credit bearing courses.

The potential offered by Web 2.0 technologies in the education of healthcare professionals, is potentially significant, however these developments need to be balanced with the inherent risks and challenges.

References

1. Salmon, G. 2002 E-tivities The Key to Active Online Learning. Routledge, UK

2. Moule, P. 2007 Challenging the five-stage model for e-learning: a new approach. ALT-J, Research in Learning Technology. 15(1). 37–50

3. Boulos, M.N. and Wheeler, S. 2007 The emerging Web 2.0 social software: an enabling suite of sociable technologies in health and health care education. Health Information and Libraries Journal 24 2-23

4. McLean, R., Richards, B.H. & Wardman, J.I. 2007 The effect of Web 2.0 on the future of medical practice and education: Darwikinian evolution or folksonomic revolution? eMJA The Medical Journal of Australia. 187 (3): 174-177 available from: http://www.mja.com.au/public/issues/187_03_060807/mcl10181_fm.html

5. Boulos, M.N., Maramba, I. & Wheeler, S. 2006 Wikis, blogs and podcasts: a new generation of Web-based tools for virtual collaborative clinical practice and education. BMC Medical Education. 6(41) doi:10.1186/1472-6920-6-41 available from: http://www.biomedcentral.com/1472-6920/6/4

9:00 AM - 10:30 AM in MaRS Auditorium

Judy Proudfoot, Black Dog Institute, University of New South Wales, Sydney, Australia; Lisa Whitehead, Centre for Postgradute Nursing Studies, University of Otago, Christchurch, New Zealand; Caryl Barnes, School of Psychiatry, University of New South Wales, Sydney, Australia; Britt Klein, Faculty of Life and Social Sciences, Swinburne University, Melbourne, Australia

Methodological Issues and Challenges in eHealth Research Panel

(Research)

Introduction: Web 2.0 technologies and approaches offer enormous advantages for consumers, health practitioners and eHealth researchers, but they also present challenges, particularly for the ethical conduct of research.**Method:** A recent systematic review of Internet-mediated research by Lisa Whitehead1 in our panel revealed that the key challenges reported by online researchers cluster into three key areas:

1. Sampling issues
Recruitment methods
Repeat participation
Identity misrepresentation
2. Ethical issues
Seeking informed consent
Anonymity, privacy and protection of confidentiality
Harm minimisation
Site security
3. Measurement issues
Validity
Reliability

This panel discusses the above challenges and profiles ethical and practical solutions to them. To stimulate audience discussion, we use our Australia / New Zealand Web 2.0 research studies below as examples of hurdles and solutions.

Research Study 1: Online help, apomediation and support from “Informed Supporters” (expert patients) to assist people recently diagnosed with bipolar disorder to take control of their condition. This study investigates whether the role of apomediaries, offered in association with an online psycho-education program, extends beyond helping users with information to collaborating, providing emotional support and giving practical help within the bounds of a serious mental illness. Ethical issues relating to confidentiality and privacy will be discussed. (Dr J Proudfoot, Black Dog Institute, Australia).

Research Study 2: The health of tertiary students: exploring on-line and postal data collections methods. This study randomly assigned 2,000 students at a New Zealand University to complete a survey on physical and emotional health either on-line or by post to explore the reliability and validity of the data collection measures and process. The study adds to debate on the potential impact of data collection mediums on results, particularly for measures of subjective symptomatology (e.g. fatigue and anxiety). (Dr L. Whitehead, University of Otago, New Zealand)

Research Study 3: A randomised controlled trial evaluating a 12-month online relapse prevention program ‘Recovery Road for Bipolar Disorder’. This study utilised online recruitment of participants through a web-based screening questionnaire, obtained informed consent through an online enrolment function and incorporated automatic randomisation into either a study or a control group. (Dr C Barnes, University of New South Wales, Australia, in collaboration with Sentiens Pty Ltd Perth)

Research Study 4: ‘ReachOut! Central’ (ROC: http://roc.reachout.com.au/flash/index) is a web-based interactive educational game using ‘real life’ scenarios and role-play to assist young people to identify and develop practical coping skills for dealing with life stressors that may be precursors to mental health problems. The study incorporated online recruitment, registration and informed consent procedures, and the use of evaluative online questionnaires and collection of participant program usage statistics. These elements, along with the unique features of ROC program, will be discussed. (Dr Britt Klein, Swinburne University, Australia, in collaboration with Inspire Foundation Australia)**Discussion:** Our panel will facilitate discussion about common ethical challenges in Web 2.0 research and collaboratively generate workable solutions to the issues.

1 Whitehead, LC. (2007) Methodological and ethical issues in Internet-mediated research in the field of health: An integrated review of the literature. Social Science & Medicine, 65, 782-791.

9:00 AM - 10:30 AM in MaRS CR2

Chair: Khaled El-Emam

Irmajean Bajnok, Toronto

RNAO eChampion Program

(Practice)

Key objective:1. To describe an eHealth champion program and its early outcomes related to supporting eHealth adoption among nurses in health care settings 2. To outline key elements of a successful eHealth champion program (lessons learned) to support eHealth adoption among nurses

As part of the Nursing and eHealth Project, funded by the Ontario Ministry of Health and Long Term Care, the Registered Nurses’ Association of Ontario (RNAO), developed an eHealth Champion Program. This program was modeled after the highly successful RNAO Best Practice Guideline Champion Program, now widely utilized in Ontario to support successful implementation of evidence based practice. The goal of the eHealth Champion Program is to support understanding, acceptance and adoption of informatics in point of care activities for nurses and other members of the health team and to raise awareness among nurses about eHealth and its link to quality patient care and the nursing role, and to support nurses in eHealth adoption across all health care sectors.

The eHealth Champion Program consists of close to 300 nurses from all sectors and across all regions of the

province of Ontario, and continues to grow. Champions are promoted by their organizations to attend workshops that are held across Ontario to prepare them with eHealth knowledge, and change strategies and processes to assist with eHealth adoption among nurses. As part of the workshop, participants receive an eHealth resource kit which includes readings, tools that helps participants determine and assess their organization's eHealth strategies, practice tools to facilitate websites, and a list of literature reference. At the end of the workshop, all participants are presented with an eHealth champion certificate. In their role as eHealth Champions, they promote nursing's involvement in eHealth for quality patient care in a variety of ways: seeking, creating and coordinating opportunity for nursing's involvement; mentoring others to promote and support knowledge transfer; proving operational support for eHealth integration within their organization; and being an eHealth resource to their organization and/ or local region.

Ongoing network meetings of eChampions are held every three months to maintain communication linkages and an opportunity to discuss successes and barriers to eHealth awareness, education and adoption; learn about strategies to promote awareness and adoption of eHealth in the workplace and update their knowledge about eHealth and informatics in nursing and health care. The Nursing eHealth Champions are also a key resource to contribute to eHealth content and policy development that affects nurses and patients. The eChampion Network provides a great opportunity for building varies networks or knowledge transfer amongst eChampions across organizations and in all health care sectors.

This presentation will focus on the recruitment and preparation of eHealth Champions, an outline of the key components of the champion preparation workshop, a profile of the eHealth champion cohort to date, and early outcomes of this strategy to support eHealth adoption among nurses. Key lessons learned related to development and use of this strategy will also be highlighted in this presentation.

Dominic Covvey, WIHIR, University of Waterloo, Waterloo, Canada

Representing and Processing Dynamic Healthcare Workflow

(Research)

Healthcare workflows are complex and highly variable. Healthcare workflow execution can be affected by any participant in a process, including clinicians, the patient, and the patient's family, as well as environmental factors such as clinician, staff, facility and equipment availability, and patient clinical status. Attempts to document healthcare workflows result in highly detailed descriptions (often incomplete) with many possible pathways to achieve the goals. A variety of graphical methods and languages have emerged to support the documentation of workflow and computer-based execution. However, only a few solutions exist that enable workflow to address the full complexity and variability of healthcare processes. We have re-conceptualized workflow and developed a new workflow representation and execution framework based on software engineering, inference engine, and database concepts, which has freed workflow representation from the constraints of past methods. We have reported our work on context-aware workflow in a separate paper.

Although much has been done on the representation of workflow in business settings, the representation of workflow in highly dynamic settings is still a focus of research. Complex dynamic environments are characteristic of healthcare. They typically involve considerable human interaction resulting in a high degree of variability in scenario outcome. Healthcare settings have many decision makers, kinds of decisions, events and a multitude of reactive, subsidiary workflows that often require a quick revision of the course of action (See Table 1). Operational and treatment protocols attempt to regularize workflow, but the needs of care, the great variety of situations and individuals, the exigencies of the moment (such as equipment failure), and the nature of human beings frustrate attempts at regularization, often resulting in protocols being labeled as "rigid" and hence being abandoned. While event sequences in healthcare processes may abide by loose constraints, they are largely non-deterministic. Therefore, it is difficult, if not impossible, to prescribe fully healthcare workflow. Instead, workflow must be dynamic, self-adapting and evolving at execution-time to match the dynamicity of the environment.

Traditional workflow technology by its very static nature supports a finite set of scenarios. In fact, traditional workflow is understood to support, at best, the union of atomic workflow patterns described by van der Aalst1. Available workflow platforms that support these patterns are often incomplete, unsatisfactory, or even non-existent. In fact, no single commercial product supports all listed patterns.1

We have developed a new way of representing healthcare processes that is able to address human-machine interaction and complexity. It converges service oriented architectures concepts with decision support techniques.

Michael Martineau, MED2020 Health Care Software Inc., Ottawa, Ontario, Canada

Consumers are not Patient(s)

(Business)

For many years Canadians considered the relationship with their physician as fundamentally different from the relationship they had with other professionals. Indeed, Canadians, like people in most other countries, use a special word – "patient" - to characterize their role in the relationship. This situation is changing, however, with people taking a more active role in looking after their health and treating physicians with less deference than they did in the past.

The emergence of the healthcare consumer is not only driving a new class of eHealth applications (sometimes referred to as Personal eHealth or Consumer eHealth applications) but will also have a profound impact on the nature of the applications as well as the choices that individuals make regarding the organizations that offer these applications. In looking

after their health and engaging healthcare providers, individuals will alternately act as either a patient or a consumer. In each role individuals will think and act differently and may consequently have a need for a different type of personal eHealth application.

While the distinction between patient and consumer may seem somewhat arbitrary and perhaps even artificial, it does help explain why new healthcare intermediaries such as revolution.com, WebMD, and Microsoft are entering the market. As well, this distinction also helps to more easily identify opportunities for healthcare providers to use information and communications technologies to engage their patients. This presentation examines the differences between consumer and patient and explores how these differences must be considered in the design of personal eHealth applications.

Edoardo Narduzzi, TechEdge, Rome, Italy

MyOpenCare: Multicontent treatment paths to share medical knowledge and experience

(Business)

MyOpenCare enables users to manage the creation and sharing of treatment plans and/or approaches in combination with the system's multimedia content. Once the "thread" has been created, the user will then be able to edit the content, i.e. with the inclusion or deletion of text or image.

The user will manage his or her content according to a graph-based structure. This information will then branch out according to the user's choices.

The creation of personal H-Books is a feature available to every user. It is designed for medical professionals interested in contributing content in exchange for increased visibility. Using MyOpenCare health professionals will be able to manage the creation and distribution of treatment and/or formative paths, which will be paths created combining the multimedia content in the system, aimed towards the production of different threads. On the nodes and edges of the path users will be able to include descriptive texts or short in-depth examinations. The user will travel along graph-based structures: on certain nodes the path will fork into two or more edges depending on the users' choices (i.e.. slightly different symptoms lead to different pathologies, or there can be multiple suggested cures)

9:00 AM - 10:30 AM in MaRS CR3

Chair: Neil Seeman

Topic: Collaborative biomedical research, academic / scholarly communication, publishing and peer review

Allan Barclay, Ebling Library, University of Wisconsin-Madison, Madison, WI, USA; Rebecca Holz, Ebling Library, University of Wisconsin-Madison, Madison, WI, USA

Development of an RSS-based Current Awareness Service

(General)

Increasingly, journal vendors are making content available via RSS feeds, but barriers still exist between the user and the content. Inspired by the FeedNavigator, a project of the National Library of Health Sciences-Terkko, we set out to develop a tool that would allow our patrons to easily locate and subscribe to journal feeds. Using a blog and a wiki for staff communication and project management respectively, we created tools that allowed the 6 members of our group to quickly capture and classify feeds for over 1,900 journals. Journal feeds are now available from our site, individually and in bundled, exportable packages (OPML files). Users can locate feeds alphabetically or by subject and can preview tables of contents before subscribing to a feed. Comprehensive bundles of feeds are offered along with select bundles of “best bets” titles.

Øystein Eiring, Norwegian Electronic Health Library, National Knowledge Center for the Health Services, Oslo, Norway; Runar Eggen, Norwegian Electronic Health Library, National Knowledge Center for the Health Services, Oslo, Norway; Magne Nylenna, Norwegian Electronic Health Library, National Knowledge Center for the Health Services, Oslo, Norway

Implementation of Web 2.0-services in the Norwegian Electronic Health Library

(Practice)

The Norwegian Electronic Health Library is a government funded website for Norwegian health personnel. The website provides national access to a wide array of scientific journals, major databases, clinical decision support, rating scales and clinical guidelines. Being a portal with predominantly static content and a one-to-many approach, the website contrasts with interactive, dynamic web services labelled “Web 2.0” where active participation from the users is necessary to expand.

Our objective was to assess benefits and limitations in different Web 2.0 services and to pilot some of the services in our website. We searched for blogs, podcasts, RSS feeds, social bookmarking sites, blogs, wikis and social networking websites aimed at health

personnel, and published in English or Norwegian. The identification of sites was performed by searching through databases, portals, websites, general search engines and reference lists. The results were published in a Norwegian medical journal.

Important benefits of the services offered include asynchronous use, automatic updates and personalization. Transparency, dialogue and more specialized communities of practice are other possible advantages of the services. Potential disadvantages include questionable quality of content, privacy concerns, problems of retrievability, ownership and a “chronic state of temporariness”. The dynamics driving Web 2.0 services in the domain of amateurs does not necessarily apply to experts.

The Electronic Health Library is gradually putting into use different Web 2.0 services on its Mental Health Specialist Library. RSS feeds from major journals and from automated searches in the Norwegian Google News is extensively used in the mental health specialist library to provide dynamic content. Through tailor-made Google Coop searches users can search for pasient information, Open Access journals and government information in a list of quality-assessed websites. Quality assessments and project plans are produced collaboratively on web-based word-processors and spreadsheets and are linked to from the website, reducing the problems of distance and version control. The mental health specialist library has established and improved more than 30 Norwegian Wikipedia entries within the mental health field, as this online collaborative encyclopedia is an important information source to patients, yet still has varying content. Our long-term goal is to move from today’s practice of extensive deep linking to automated services, which would decrease the workload as well as make our content more updated and complete. We have thus suggested to the Cochrane Library and to the Guidelines International Network that they make their new reviews and guidelines available with the use of topic specific RSS feeds.

Six months after starting applying Web 2.0 services in our website, the share of refferals from search engines has doubled. The number of front page views in the specialist library has increased by 15 percent during the period. It has proved difficult to evaluate on a more specific level the results of the adjustments.

References:

www.helsebiblioteket.no

www.helsebiblioteket.no/Psykisk+helse

Eiring et al. Blogg, RSS og podkast i medisin og helsefag. Michael 2008;5:24-39.

Moshe Pritsker, JoVE (www.jove.com)

Online video-publication for biological research

(Business)

Journal of Visualized Experiments (JoVE, www.jove.com) was founded in October 2006 as the first online journal devoted to video-publication of biological research. This new method of scientific publishing allows visualization of experimental studies consequently increasing reproducibility and efficiency of research in biological sciences. The JoVE Editorial Board includes 20 scientists from Harvard, Princeton, NIH, and other institutions. JoVE has so far released 12 monthly issues that include about 200 video-protocols on experimental approaches in neuroscience, immunology, developmental biology, microbiology and other fields. To facilitate integration of video into scientific publishing, JoVE has developed an organizational and technical structure to perform and/or assist scientists with filming and editing in their labs.

Press about JoVE:

WIRED: http://www.wired.com/science/discoveries/news/2007/10/jove

The Scientist: http://www.the-scientist.com/news/home/53500

Nature: http://www.nature.com/news/2006/061120/full/061120-12.html

11:00 AM - 12:30 PM in MaRS Auditorium

Chair: Holly Witteman

Topic: Consumer empowerment, patient-physician relationship, and sociotechnical issues

Luis Fernandez Luque, NORUT AS, Norway; Ignacio Basagoiti, ITACA-TSB Research Institute, Technical University of Valencia (Spain); Elin Johnsen, Norwegian Center of Telemedicine (Norway); Randi Karlsen, Department of Computer Science, University of Tromsø (Norway)

Study of the ePatient as a provider of health content in the Internet

(Research)

INTRODUCTION: Millions internet users publish information about their daily life (1) and also about their health. There is little research evidence (2) about the usefulness and trustworthiness of the Patient Generated Content (PGC) and how it could affect the doctor-patient relationship. To study these aspects we designed a web-survey for chronic patients who are actively publishing in the Internet, gathering their views as consumers and creators of PGC.**METHODS:**A survey was designed and a private invitation to participate was sent to 122 patients. We searched for patients in Google (Web, Video and Blog Search Engines), Patient’s Portals and the links found. The participants were informed and asked for their consent.**RESULTS:**28 completed surveys were submitted. 53% of the responders were female, the average age was 43,6 years (SD = 13) and 96% had at least some college studies.

The PGC got the highest rating in usefulness and trustworthy comparing with content from other authors (Pharmaceutical companies, Governmental Agencies, etc.). However, some responders think that some PGC is false (29%).

The PGC was especially helpful to find emotional support (93% agreed or strongly agreed) and to learn from experience of others (96%). PGC was considered also helpful to: prepare for an appointment, complement the doctor's information, manage better their health, acquire a healthy lifestyle and adjust medical treatment. Most of the patients (64%) have discussed the PGC found with their doctors and their reactions were mainly positive (33%) or neutral (55%).

In the previous 3 months, the mean of publishing post in blogs or personal webs was 23, 16 in other patient´s sites and 3 videos. All the patients publish information classified as confidential by the HIPAA, e.g. 75% publish full face photos. The most common medical information published is diagnosis (86%), treatment (79%), date of diagnosis (71%) and relapses (61%). Patients publish also about their healthcare professionals: general opinions (68%), names (25%), working place (21%) and complaints (17%). The 68% of the patient's doctors knew that they are publishing, and their reactions were positive (76%) or neutral (23%).**DISCUSSION:**PGC is highly rated in terms of usefulness and trustworthy. The reactions of the doctors to PGC were positive, both as consumers and as generators of PGC. However, in our opinion, there is a risk of damaging the doctor-patient relationship by using the web to complain about health professionals. As the rest of the health information providers the patients need ethical guidelines. This could be addressed by promoting the use of the well-known publishing ethical guidelines, such as HON, among the patients. Nevertheless, to adapt them to the needs of the patients, it is necessary to study more the PGC. In our opinion, the patients should be considered by the research community as providers and not mere consumers.

REFERENCES:

1. Lenhart A; et al. Content creation online. Pew Internet & American Life Project. 2008-04-30. URL:http://www.pewinternet.org/pdfs/PIP_Content_Creation_Report.pdf . Accessed: 2008-04-30.

2. Sujin K; et al. Characteristics of cancer blog users. J Med Libr Assoc. 2007 October; 95(4): 445–450. doi: 10.3163/1536-5050.95.4.445.

Joanne Mayoh, Health Informatics: Bournemouth University

Will the development of Web 2.0 technology result in a preference for quantity over quality?

(Practice)

Over the last decade the world has experienced a rapid increase in the number of people using the internet for health information seeking and interaction. This increased demand for internet heath information has led to the emergence of large volumes of unstructured and unregulated medical information found on the web. This web-based information is seldom the subject of systematic investigation for its accuracy and appropriateness for patients, highlighting the need for additional research into the field. The rapid development of the internet as a communication tool, and the accumulation of large volumes of information, makes locating valid information extremely difficult especially for those who lack ability to use computers and technology efficiently. A 1997 study examining 40 websites providing advice on the management of a feverish child found that only four of the websites adhered to published guidelines. These high volumes of unregulated internet health information are partly due to there being no agreed standard for the assessment and to ensure accuracy of information presented on the web. This paper addresses the issue of quality of online health information, and asks, as we move forward and develop technologies which emphasise content creation, will the issue of quality of online health information be exacerbated?

The emergence of Web 2.0 has facilitated many new online activities which could not have previously been achieved by the world wide web (Web 1.0) technology. Often referred to as the 'social web', it looks to increase social interaction by sharing content which is more easily generated and published by users. Many researchers believe this has implications for the future of healthcare, by enhancing the connection between patients, clinicians and health information. The use of blogs, podcasts and wikis can help to both communicate health information, and increase social support by providing a potentially anonymous space within which online interaction can take place. It also allows the information seeker to rapidly become the information provider, and share healthcare knowledge and experiences quickly and easily. However, this benefit of Web 2.0 technology may also be seen as a limitation when discussing information quality. For example, although health related Wiki's and blogs are an excellent way to share health information, their content can be added and edited by anyone, with a significant number being fuelled by lay users, most often with no professional experience of the health topic they are writing about.

This paper aims to discuss that while the benefits of using Web 2.0 to develop communication in healthcare and health education are clear, it is also necessary to consider potential issues, such as quality, which may be more present in Web 2.0's application to healthcare than other uses. This issue is made relevant due to the possibility of high volumes of inaccurate and misleading information being potentially disastrous within a health care setting, as it can be extremely distressing and potentially damaging for some health information seekers.

Sheryl Mitchell, Women's College Hospital

Women Wading Through the Web: providing women with the tools to evaluate online health information

(Practice)

Background: Using the Internet, women can now access a broad range of medical information in their own homes with relative anonymity. It is assumed that "greater availability of health information via the Internet will lead to the emergence of more informed patients who are better able to assess the risks and benefits of different treatments for themselves." [1] Yet the solution

is often flawed. Health information on the Internet is often inaccurate and misleading. Given that anyone with Internet access can easily create and distribute online content, concerns about the credibility of health-based information are especially relevant. It is vitally important that women are given the tools to assess the accuracy and quality of online health information.

Criteria for assessing online health information: Health librarians have developed criteria for assessing the quality of online health information. These include instruments and checklists intended to establish whether content is credible, [2] and web interfaces usable. A review of published criteria for evaluating health information on the web reveals that many authors agree on key criteria for evaluating health-related websites. [3] Experts advise Internet users to check a health site's source and currency, and to visit several sites in their search for information.

While health librarians clearly know how to assess the quality of online health information, the general public usually does not. Only one-quarter of respondents to a survey on the use of strategies to separate "good" information from "bad" online health information [4] said they follow the recommended protocol of thoroughly checking the source and timeliness of a website's information. This puts women who use online health information resources at serious risk for obtaining, and acting upon, false and misleading information.

If women are to use online health information resources effectively, they need accessible and plain language educational tools and best practices information for assessing the quality of information presented.

Online Health Toolkit: This paper describes how the staff at womenshealthmatters.ca, Women's College Hospital's consumer website, provided women with the tools to assess the accuracy and quality of online health information. Women Wading Through the Web was designed to provide women with educational tools and best practices information on how to use the Internet to search for health information, judge the quality of online health information, understand medical research and analyze health stories in the media. The presenter will also discuss the challenges of using a dedicated web community to facilitate discussion about accessing reliable online health information and to evaluate consumer satisfaction with the Toolkit.

References

1. Henwood F, Wyatt S, Hart A, Smith J. Ignorance is bliss sometimes: constraints on the emergence of the 'informed patient" in the changing landscapes of health information. Sociol Health Illn. 2003 Sept: 25(6): 589-607.

2. Eysenbach, G, Powell J, Kuss O, Sa ER Empirical studies assessing the quality of health information for consumers on the world wide web: a systematic review. JAMA. 2002 May 22-29: 287(20): 2691-700.

3. Kim, P, Eng TR, Deering, MJ, Maxfield A. Published criteria for evaluating health related web sites: review. BMJ. 1999 Mar 6;318 (7184):647-9

4. Vital Decisions: How Internet Users Decide What Information to Trust When They or Their Loved Ones are Sick. (Pew Internet & American Life Project: May 22, 2002).

Cornelia Van Uden-kraan, Institute for Behavioural Research, University of Twente, Enschede, The Netherlands; Constance Drossaert, Institute for Behavioural Research, University of Twente, Enschede, The Netherlands; Erik Taal, Institute for Behavioural Research, University of Twente, Enschede, The Netherlands; Erwin Seydel, Institute for Behavioural Research, University of Twente, Enschede, The Netherlands; Mart Van De Laar, Institute for Behavioural Research, University of Twente, Enschede, The Netherlands

Empowerment by Participation in Online Support Groups for Patients with Arthritis, Fibromyalgia and Breast Cancer

(Research)

Background: Ever since the rise of online support groups much is expected of the potential empowering effect of participation for patients [1]. Although many have pointed at this empowering effect [2, 3, 4, 5], little empirical evidence exists for how patients feel empowered.Objectives: We explored if and how people with arthritis, fibromyalgia and breast cancer feel empowered by their participation in online support groups. In addition, we were interested which processes that take place in online support groups contribute to these outcomes. **Methods:** We sent a posting with a link to an online questionnaire to 19 online patient support groups. The questionnaire included questions on demographics, use of the support group, empowering processes (exchanging information, finding recognition, sharing experiences, encountering emotional support and helping others) and empowering outcomes (being better informed, feeling more confident in the relationship with their physician, improved acceptance of the disease, feeling more confident about the therapy, enhanced self-esteem, enhanced social well-being and increased optimism and control). All items concerning the outcomes had the format of a statement that began with 'Through my participation in online support groups…'. Scores on all scales ranged from 'not empowered' (1) to 'very empowered' (5). In total 528 participants responded to the request to fill in the survey (23% arthritis, 22% fibromyalgia, 41% breast cancer). **Results:** The majority of the respondents were women (n=494) with a mean age of 44 years (SD 10.4 years). The respondents were diagnosed 5 years ago on average (SD 5.9 years). The respondents felt empowered in several ways by their participation. With the exception of 'increased optimism and control' all scale scores for empowering outcomes were above 3.1. No significant differences in empowering outcomes between diagnostic groups were found. The empowering outcome that was experienced to the strongest degree, was 'being better informed'. For example, in total 74% of the patients had the feeling that they had the right knowledge to manage their illness. The empowering outcomes 'feeling more confident in the relationship with their physician', 'increased acceptance of the disease', and, 'feeling more confident about the therapy' were experienced to the same

degree. The participants indicated, for example, that they knew better which questions to ask their physician (61%). For many respondents, participation in an online support group enabled easier acceptation of the disease (47%) and helped them to 'open up' about their disease (55%).

The empowering outcomes could only be partially predicted by the processes. The most important predictors of the outcome 'being better informed' appeared to be 'exchanging information' and 'finding recognition'. In total, 31% of the variance could be explained. The most important predictors of the outcome 'enhanced social well-being' appeared to be 'encountering emotional support' and 'sharing'. In total, 30% of the variance could be explained.**Conclusions:** This study showed that participation in online support groups can make a valuable contribution to the empowerment of patients. Healthcare providers should acquaint their patients with the existence of online support groups and with the benefits that participation in these groups can offer.

References

[1] Ferguson T. Health online and the empowered medical consumer. Joint Committee Journal on Quality Improvement 1997;23(5):251-257.

[2] Shaw B, McTavish F, Hawkins RP, Gustafson D, Pingree S. Experiences of women with breast cancer: exchanging social support over the CHESS computer network. Journal of Health Communication, 2000;5(2):135-149.

[3] Sharf BF. Communicating breast cancer on-line: support and empowerment on the internet. Women & Health 1997;26(1):65-84.

[4] Høybye MT, Johansen C, Tjørnhøj-Thomsen T. Online interaction. Effects of storytelling in an internet breast cancer support group. Psycho-Oncology 2005;14(3):211-220.

[5] Barak A, Boniel-Nissim M, Suler J. Fostering empowerment in online support groups. Computers in human behavior 2008:doi:10.1016/j.chb.2008.02.004.

11:00 AM - 12:30 PM in MaRS CR2

Chair: Michael Martineau

Topic: Personal health records and Patient portals

Miguel Cabrer, eHealth Advisor; Francisco Tous, IBIT Institute - Balearic Government; Debora Martinez, IBIT Institute - Balearic Government;Pedro Ferriol, IBIT Institute - Balearic Government

Virtual Health Platform

(Business)

The interoperability of Electronic Medical Records is a worldwide problem difficult to solve and with lots of resources involved and working on it. There are some standards to interchange information that are being implemented and good advances have been made.

But the reality is that the clinical information is poorly shared in regions and countries and even less between countries. That means that a citizen that moves between countries has no guarantee that, if something happens and medical service in requiered, the clinicians have the right information to treat the patient.

At a european level the problem is adding the fact that the european citizens have the right to have health services at any european contry. The multilingual problem, poor interoperability implementation technology makes the solution difficult to achieve. For that reason the EU is funding projects through the FP7 that pretend to achieve the "Health without barriers".

In the context os the Innovative European Program (FEDER 2000-2006) a Balearic Islands (Spain) consortium has defined and implemented a project that pretends to provide a solution for Electronic Patient Record Interoperability between european countries. Such a project has been evaluated in the Medical Tourism environment, dinamic sector happy to provide high level service to tourists.

The implemented solution (Virtual Health Platform) consists on a basic interoperable platform with this characteristics:

- A personal health record with the basic and resumen relevant clinical information from the patient (diagnosis, medication, allergies) but codified with SNOMED a worldwide multilanguage medical standard terminology.
- A virtual asyncronous teleconsultation solution that allows referral physician to ask for a second opinion or to referer a patient. It allows to add interconsultation information plus images and videos in Dicom Format (also for interoperability reasons)
- The interoperable platform allows to send and receive HL7 messages
- The platform is integrated with a USB Devices and allows to store the medical data into a secure USB bracelete.

The platform allows to cover the workflow:

1. the patient that is going to travel can store some critical data on the Virtual Health Platform. If travelling because of a medical tourism process can also perform a teleconsultation and send some presurgery information or images.
2. The source health center received the information and can manage the teleconsultation and schedule the patient.
3. To treat the patient the physicians can access to the personal health record and get the information into a local language (SNOMED performs the translation).
4. If there is an emergency or connection problem the information can be reviewed trought the usb bracelete.
5. when the patient has already been treated and has to go back home (other country) the information is stored in the personal health record platform.

The Virtual Health Platform acts as Medical Record Gateway allowing access to information between countries, translating the clinical content and integration with other information systems.

As a result of the project there are two outpus:

- the virtual health platform
- a paralel study of medical tourism business cases and requirements

The project is one more step in the interoperability problem that europe is trying to reach. To focus in a very dinamic sector like tourism has been good to achieve concrete results fast. The standard terminology

SNOMED has helped to get translation and to better organise the medical record. Standards are necessary.

Deborah Chan, University Health Network; Selina Brudnicki, Shared Information Management Services

InfoWell Patient Portal: A Case of Patient-Centred Design

(Practice)

ObjectivesThere is still much to understand about the benefits of Patient Portals and patient access to their electronic health record. Specifically usability best practices for their design and development and insight into what patients really want to see in a Patient Portal. The InfoWell Patient Portal is a tool designed to assist patients in the management of a chronic condition by providing education, and improving access, support, and navigation through the continuum of care. The objective of the usability component of the InfoWell Patient Portal project is to engage patients throughout the design and development phase to ensure the final end product meets user needs in terms of content and tools, and is easy to use in order to encourage user adoption. **Context/Situation** The InfoWell Patient Portal represents the largest comprehensive personalized health information website in the province that spans the continuum of care. The SIMS Partnership, the information management and technology (IM/IT) group shared between thirteen health care organizations in the Greater Toronto Area, has led its development.The InfoWell Patient Portal was developed in response to the demand for greater access to health information for patients. Breast cancer, diabetes, and chronic kidney disease patients were chosen in particular due to the perceived benefits of being more educated, informed, and proactive, enabling self-management and ultimately resulting in improved health outcomes, for these particular groups.**Method**The InfoWell Patient Portal utilized a user-centred design (UCD) methodology to gather requirements, insight, and feedback from patients. This provided health care and IT professionals, and researchers an enlightened perspective as to what patients really want. The requirements gathering process involved an ethnographic approach of using patient interviews and surveys. Patients helped to improve content organization, navigation, menus, and taxonomy of the website through a "Card Sorting Method". Several stages of iterative user testing were conducted to determine the ease of use of the website and to collect suggestions on future tools and functionality for the portal. **Benefits**Involving end-users in the design and development of the InfoWell Patient Portal help to ensure that 1) The content and tools are useful and meet the needs of patients, 2) The website is intuitive and easy-to-use in terms of design and navigation, thereby increasing the likelihood of widespread adoption by end-users. **Results** Results of usability testing were incorporated into the iterative design cycle for the Patient Portal and resulted in continual improvement in terms of ease of use with regards to navigation, content groupings and labels, with potential for scalability of disease groups and additional partner organizations. In particular, the card sorting exercise significantly improved the content organization of the website to better correspond to patient expectations.

While it is important to engage patients in the requirements gathering phase, it has proven essential to directly observe patients' interaction with the website and tools through iterative usability testing in order identify any disconnects between the goals of health care organizations for the portal, and those of the patient.

Zach Landis Lewis, Department of Biomedical Informatics, University of Pittsburgh, Pittsburgh, USA; Gerald Douglas, Department of Biomedical Informatics, University of Pittsburgh, Pittsburgh, USA; Valerie Monaco, Department of Biomedical Informatics, University of Pittsburgh, Pittsburgh, USA

Measuring Efficiency of Use in a Web-based EMR Developed for Malawi: Lessons Learned From Performing a Preliminary CogTool Analysis

(Research)

Background: Developing Web-based electronic medical record (EMR) systems is a fundamental step in enabling resource-constrained healthcare environments to benefit from Web 2.0 applications and services. Towards this goal, we have developed and implemented a Web-based EMR in Malawi, Africa. The EMR, built using Ruby on Rails and AJAX, features touchscreen workstations that collect and validate data from the clinician in real-time at the point of care. The system is deployed in high patient burden HIV/AIDS clinics that have frequent staff turnover. This context makes two aspects of system usability – learnability and efficiency of use[1] – important design considerations.**Objective:** To determine how efficiently novice users complete tasks using the touchscreen interface of the EMR. **Methods:** To predict how quickly a user could be expected to perform the EMR tasks, we used an established technique for predicting skilled performance time called the Keystroke-Level-Model (KLM)[2]. This technique models the low-level perceptual motor operators required to complete a specified task using a given interface. KLM calculations require the application of a significant amount of cognitive modeling information. However, a free software application called CogTool[3] has been developed to support cognitive modeling for KLM. In the first phase of our research, we selected thirty-one routinely performed EMR tasks and conducted a KLM analysis of the tasks using CogTool. The interfaces and user actions for each task were prepared within CogTool using a storyboard approach. CogTool analyzed the storyboards to predict performance time of a skilled user (i.e., one who performs without error or hestitation). In the second phase, we recruited four volunteers who had not previously used the EMR to complete the thirty-one tasks for three mock patient encounters (resulting in 372 task observations). We collected the task performance data by modifying the EMR to collect timestamp data for all interface events. In the third phase we compared the

preliminary novice performance data with the CogTool predictions.**Results:** Of the 372 EMR tasks, 77% (286) were performed without errors by the novices (errors defined as any deviation from the CogTool storyboard). Of these error-free tasks, the performance time for 68% (194) was faster than the CogTool prediction. We observed two unexpected user behaviors not captured by the initial CogTool models: (1) two-handed touchscreen interface use, and (2) prolonged dialogue during patient interaction (e.g. spelling verifications). **Conclusions:** Although preliminary in nature, our initial data suggests that novices can sometimes achieve the efficiency level of a skilled user of the EMR. We found CogTool to be an effective software application that permitted us to rapidly develop predictions of skilled performance time. The ability of most novices to often outperform the KLM calculations was likely related to a documented weakness in CogTool regarding the insertion of "think steps" for all touch typing. In our case, for onscreen typing tasks, CogTool inserted "think steps" before each button press. However, upon review it was not possible to identify suggested inclusion criteria for all of these "think steps" [4]. We are currently revising the CogTool models before collecting additional empirical data.

References

1. Nielsen J. Usability Engineering. 1st edition. New York: Morgan Kaufman; 1993.

2. Card SK, Moran TP, Newell A. The Keystroke-Level Model for User Performance Time with Interactive Systems. Commun ACM. 1980 Jul;23(7):396–410.

3. John BE, et al. (2006). The CogTool Project. http://www.cs.cmu.edu/~bej/cogtool/index.html. Archived at: http://www.webcitation.org/5Xayftaav

4. Kieras D. (2001). Using the Keystroke-Level Model to Estimate Execution Times. ftp://www.eecs.umich.edu/people/kieras/GOMS/KLM.pdf

Barbara Purin, Bruno Kessler Foundation (FBK), Trento, Italy; Emiliano Ricci, Bruno Kessler Foundation (FBK), Trento, Italy

User-centered Design of a PHR: Traditional Web Forms vs. Wizard Forms

(Research)

Background: This work is part of an ongoing feasibility study aimed to design and deploy a PHR system for the citizens of the Province of Trento (NE Italy). The system is intended both to support storing health information important to patients and to improve relationships and communication between patients and their health care providers. With regard to the first topic, input data forms and navigation are crucial elements that would be used consistently in order not to compromise the use of the system. That‘s why considerable effort was dedicated to guide and support the management of health information in the system design phase. We adopted a citizen-centered approach within an iterative design-evaluation process. This work is supported by the Department of Health and the Department of Research and Innovation of the Autonomous Province of Trento (NE Italy).**Objective:** Our objective was to explore the effectiveness of the traditional web forms vs. wizard step-by-step structure and to gather information about problems that users may encounter when interacting with such interfaces. We focused on testing usability and user experience of two user interfaces within an interaction design approach [1]. The two prototypes were designed and developed to support citizens to maintain drugs' information themselves.**Methods:** An early evaluation by health researchers working in FBK was performed. In a second time interaction design methods were used for comparing the two user interfaces. We adopted a usability testing based on the think-aloud technique for observing users while surfing the system and filling the forms, post-task questionnaire based on a Likert-type scale for assessing user satisfaction, and debriefing semi-structure interview for exploring subjective user ex-perience behind what was previously observed [2]. According to Nielsen [3], eight participants were recruited among the administrative personnel of FBK. The inclusion criterion was the skill in using the web. The used task scenarios were stories of common drug prescription.**Results:** The first evaluation identified mainly navigation problems from screen to screen. A brainstorming with the health researchers allows us to reorganize internal navigation according to the critiques. The usability testing was carried out on the refined user interfaces. Think-aloud reports were transcribed in a world processing file and a content analysis was performed using coding categories described in literature about human-computer-interaction [2]. The most frequent problems were related to the selection of malaises and diagnoses from the related lists. These lists were difficult to use since they were based on large medical terms organized in apparatus. The other issues were about navigation: the button for adding a new prescription in the main page need to be more highlighted and the tab label for editing personal evaluation should be reworded for clarity.**Conclusions:** Repeated cycles of design-testing-measure-redesign allow pointing out wrong design assumption that could cause usability problems later. No one system prevailed against the other and the incidental preference coming from the debriefing interview was founded on subjective impressions. It could be interested to repeat this test after people have used the wizard interface for a period of time.

References:

1. Preece, J. Roger, Y. and Sharp, H. Interaction design: beyond human-computer interaction. New York, John Wiley & Sons, Inc.

2. Kushniruk, Andre W. and Patel Vimla L. Cognitive and usability engineering methods for the evaluation of clinical information systems. Journal of Biomedical Informatics, 37, 56-76, 2004

3. Nielsen, J. Usability engineering. Academic Press, San Diego, CA, 1993

11:00 AM - 12:30 PM in MaRS CR3

Chair: Suzanne Dickerson

Topic: Building virtual communities and social networking applications for health professionals

Carol S Bond, Bournemouth University

Stop The World wide web I Want To Get Off.

(Research)

BackgroundIncreasing number of patients are using the Internet for health information (National Statistics 2007). Traditionally professionals have been the information gatekeepers; key questions were around what information patients should be given. Reviews of information on the Internet have been focused towards analysing the quality of information e.g. Eysenbach et al [1] and Breckons et a 2008[2]

Whilst this debate is important patients do not always want this professional centric world and are quietly seizing the agenda. If the professionals are not giving them the information they want, they are going and finding it for themselves.

Neither patients nor professionals however yet understand enough about the new options that the Internet affords to be able to develop a shared understanding of how to make best use of it. Web 1.0 brought debate around the quality of the information made available. Web 2.0 however brings the additional complexity of knowing where the information is before it can be questioned. One weakness of some research into health information on the Internet is that a quite common research method is to carry out searches and then analyse the top results (e.g. Biddle et al[3]), which tends to exclude information from Web 2.0 sites. **Objective**Healthcare professionals are one of the main sources of information for patients; their knowledge of information sources will affect their ability to support their patients. The objective of this research was to explore professionals' use of the Internet for patient information. **Methods**A questionnaire was distributed to healthcare professionals attending CPD sessions at an English university. Participation was voluntary, and students assured that this did not affect the session they were attending. The researcher was not involved with the session. **Results** In total there were 51 completed the questionnaires. 71% of respondents were nurses. The majority (94%) thought the Internet was a good source of health information. Their use of the Internet was however limited. Most (85%) often used email, far fewer often used instant messaging (29%) or discussion boards (20%). Only 23% reported having an Athens account, the Health Service system for accessing online journals. Few (18%) would usually recommend a website to patients, 33% would never do so. Even less, 8% would usually recommend an online support group, over half (57%) would never do so. Not only were these professionals not recommending the Internet to their patients, they were not using it to locate patient information themselves. Only 16% said they usually used websites that allowed them to create tailored information leaflets for their patients, 32% never using these sites. **Conclusion**Although these healthcare professionals thought the Internet was a good source of health information this belief was not matched by their actions. Less than one third usually recommended the Internet to their patients, more never did so. Nor were they using the Internet to meet their own information needs. Healthcare professionals education needs to include the Internet as a patient information source so that they are able to advise and support their patients.

Shirley Fenton, WIHIR, University of Waterloo, Waterloo, Canada; Dominic Covvey, WIHIR, University of Waterloo, Waterloo, Canada

Creating and Supporting a Community of Practice

(Develop)

Aims: We have investigated the nature of a Community of Practice (CoP), particularly as applied to healthcare and health research teams. Our objectives are to define frameworks for collaboration and provide information systems support for collaborative teams. Although the "CoP" term is widely used, it appears to be minimally understood, as is how to realize and support CoPs. **Methods:**Our work has involved three thrusts: (1) to understand the literature on the CoP concept and experience with its application, (2) to convene a Ministry of Research and Innovation workshop featuring progenitors of the concept together with researchers and other parties, and (3) to analyze in depth the needs of a specific CoP – a team of cancer researchers. **Results:**A very successful workshop featuring Dr. Etienne Wenger exposed its participants to the nature and realities of CoPs and defined the requirements for facilitating the creation of CoPs. The video archive of this workshop has been made available on the Web. The study of the needs of cancer researchers resulted in two key reports, one on the nature of a researcher CoP and its required technological underpinnings, and one on the concept of operations of a CoP-support system.**Conclusions:**We all seem to realize the value of the CoP idea, but few have delivered practical, usable tools that help to assemble a CoP and support it. Our work provides this basis, and we will present what establishing a CoP requires and how it needs to be supported by information systems.

Chris Paton, Health Informatics Programme, Department of Information Science, University of Otago, Dunedin, New Zealand

New Media Medicine: A Social Network for Doctors and Medical Students

(Business)

New Media Medicine hosts an online Social Network with over 40,000 registered members.

The network primarily caters for UK doctors, medical students and pre-med students applying for medical school. Features include a lively discussion forum with

over 500,000 posts, and facilities for members to blog, upload photos and create social groups of friends.

This presentation describes the tools and techniques required for building a friendly and successful medical social networking community. In particular, we examine online community dynamics, moderation techniques and strategies for growth and sustainability.

The presentation discusses how a social network can be used as a basis for an international e-learning community through the use of user-generated content and online peer review and discussion.

Ken Seto, OntarioMD.ca

Facilitating Online Collaboration and Communication: A Groups Approach

(Practice)

OntarioMD Groups (www.ontariomd.ca/groups) is an online collaboration tool designed to facilitate communication and workflow among physicians and other healthcare professionals. By providing an external web presence and a private intranet suite of tools, OntarioMD Groups is a versatile way to plan events, share documents, and engage in discussion with peers.

The OntarioMD Groups project is the first iteration in a fully user-driven communication and collaboration platform. The project is currently being piloted with a diverse population of both clinical and non-clinical users. The presentation will focus on the development of the project, results of the pilot and future plans.

References

OntarioMD Groups homepage. URL: http://www.ontariomd.ca/groups

1:30 PM - 3:00 PM in MaRS Auditorium

Chair: Peter Pennefather

Topic: Consumer empowerment, patient-physician relationship, and sociotechnical issues

Dafna Carr, Cancer Care Ontario; Steve Hall, Cancer Care Ontario

Using Technology to Engage Patients and Clinicians in Electronic Cancer Symptom Assessment and Management: Lessons Learned and Implications for Practice

(Practice)

Many cancer patients struggle with the physical and emotional pain caused by cancer symptoms. Patients and care providers often find it challenging to effectively communicate their symptoms because of minimal use of standardized symptom assessment tools and inconsistent pain and symptom management practices. Paper tools are the norm but they are not accessible to all team members, cannot be viewed across the system unless they are manually entered into a database, and are not easily shared across care settings (e.g. hospitals, home care) or able to show a patient's symptom severity scores over time.

The Interactive Symptom Assessment and Collection (ISAAC) tool developed by Cancer Care Ontario (CCO) is an easy-to-use, standardized, secure, electronic tool that allows patients to complete an interactive version of the Edmonton Symptom Assessment System (ESAS) tool 1. ISAAC puts patients in control of their own symptom assessment and engages them directly in the symptom management process. Patients simply enter their symptom severity scores for nine common cancer symptoms electronically on a touch-screen computer kiosk, which sits at their regional cancer centre, or from their home computer via the internet. Clinicians can access a patient's symptom information, regardless of where the patient entered their scores – clinic, home, or at another cancer centre – and can track this information over time and across health care settings. ISAAC also provides Cancer Care Ontario with the data to report on symptom screening across Ontario and the patient experience.

Preliminary evaluation of ISAAC has been positive. Users of ISAAC surveyed gave high ratings to its ease of use and clarity. Clinicians and other stakeholders reported that it allows for better management of the disease if symptoms can be detected as they arise, in real-time. The patient histograms were seen as extremely useful in care management, by providing clinicians with a snapshot of a patient's experience with various symptoms and a view of this experience of over time.

This presentation will provide an overview of the ISAAC tool and its development. Challenges in facilitating home-based use of ISAAC will be discussed along with issues related to system integration with electronic records and the electronic challenge of supporting the patient/clinician interaction across care settings.

Suzanne Dickerson, School of Nursing University at Buffalo, Buffalo, NY; Grace Dean, School of Nursing, University at Buffalo

Developing of a web-based application to facilitate patient treatment adherence in individuals with sleep apnea treated with Continuous Positive Airway Pressure (CPAP) devices.

(Research)

Background: Obstructive sleep apnea (OSA) is an increasingly common sleep disorder in individuals whose airways obstruct and become frequently apneic during sleep. Persons diagnosed with OSA are at risk for serious health problems such as hypertension, stroke, and arrhythmias including daytime sleepiness and poor waking cognitive function that increases their risk for motor vehicle and work related accidents [1]. The most common treatment for OSA is the use of a Continuous Positive Airway Pressure (CPAP) device which serves as a pneumatic splint to keep the airway

open. Many people have difficulties in adapting to the device, modifying their sleep routine, and thus discontinue the treatment. In a study of experiences of OSA patients in the first three months of CPAP use, researchers followed the patients by questioning and evaluating of the CPAP use which helped the patients understand the often subtle improvements in their symptoms of sleep deprivation which hampered motivation to adhere to treatment [2]. Another study documented the effectiveness of a CPAP support group in sharing difficulties, offering suggestions, and empowering patients to problem solve their difficulties [3] which suggest that social networks introduced in an online application would provide at-home support to improve adherence. Research indicates that education alone does not improve adherence rates; however, instituting cognitive behavioral therapy may be effective [4]. Therefore the chosen approach focuses on improving self efficacy (confidence), evaluation of barriers, and developing behavioral strategies strengthened by social support to improve adherence rates.**Objective:** The purpose of this paper is to discuss the steps in the development of a web-based interactive application to facilitate patients' adherence to CPAP treatment. The goal of the web- based application is to facilitate adherence by creating a patient-managed tracking system for symptoms and CPAP usage, self-assessment of barriers, motivational interactive questioning, and a resource library including patient success stories. This application will facilitate developing behavioral strategies to promote adherence that can be accessed early in the treatment phase, when risk of non-adherence is high. **Method:** Application of Self Efficacy theory [5] to interactive web based design. **Results:** The CPAP motivational application will provide a web-based interactive patient self- management program that will: 1.) Capture user data by collecting self-reported CPAP use survey data; 2.) Track patient progress over time; 3.) Provide automated motivators; 4.) Provide a resource library with motivational success stories; and 5.) patient discussion forum. **Conclusions:** Use of theory based behavior modification approaches can in integrated into a web based application for future intervention study to test feasibility and efficacy.

References:

1. Young T, Peppard P, Gottlieb D. Epidemiology of obstructive sleep apnea. A population health perspective. Am J Respir Crit Care Med 2002; 165:1217-39.

2. Dickerson SS, Akhu-Zaheya L.Longitudinal study of CPAP patients beginning treatment. Rehabil Nurs 2007;.32(6):241-50

3. Dickerson SS, Kennedy M. Seeking encouragement in a support group of individuals with sleep apnea who use continuous positive airway pressure (CPAP) devices. Rehabil Nurs 2006; 31(3),114-122.

4. Haniffa M, Lasserson TJ, Smith I. Interventions to improve compliance with continuous positive airway pressure for obstructive sleep apnoea. Cochrane Database of Systematic Review 2004 Issue 4. Article no CD003531.DOI:10.1002/14651858.CD003431.pub2.

5. Bandura A. Self Efficacy: The exercise of control 1997; NY:W. H. Freeman

Matic Meglic, UP PINT, Koper, Slovenia

Web and mobile solution for support of process of care: case of depression

(Research)

Introduction: In general practice 12% of patients are suffering from depression, which puts depression on the second place of most common chronic diseases in primary care. Results of DEPRES study have shown that only 1,5 % are properly treated – they are either undiagnosed, receive improper treatment or discontinue treatment and are not followed-up. Several interventions improve outcome of treatment: education of staff, care management, active patient involvement including self-assessment, follow-up with telephone reminders and access of general practitioner to patient progress results. All of these can effectively be joined together using an information system, employing workflow management. A recent study by Robertson et al has shown promising results of an internet based solution to provide several of these functionalities.**Methods:** to evaluate an integrated care management and patient involvement approach to treatment of depression a web-based information system has been developed, accessible by all actors in care process using computers and mobile phones. The solution enables process support through defining tasks and timelines to all involved actors, automated reminders, communication tool, access to patient data etc. Use of process matrices enables complex evaluation of patients' response to treatment. The effects of the approach in which all involved actors use the application as care support are being evaluated in a case-control study involving general practitioners and patients diagnosed with depression for the first time. A multivariate analysis of factors influencing patient compliance and treatment outcome will be conducted. **Results:** the research will show which functionalities of the system and related care management or patient empowerment approaches contribute to what extent to depression treatment outcome. Generalised model of defining and structuring the care process and the modalities of the ICT support has been tested. **Discussion:** information technology supported care management of chronic diseases with focus on active patient involvement and patient empowerment emerges as one of the most promising ways to ensure cost-effective way of treatment. It is in most cases tightly connected to organization change and financing change, To insure sustainability these rather complex financing and organizational issues need to be resolved.

Nicol Nijland, Department of Psychology and Communication of Health and Risk, Faculty of Behavioural Sciences, University of Twente; Lisette Van Gemert-pijnen, Department of Psychology and Communication of Health and Risk, Faculty of Behavioural Sciences, University of Twente; Saskia Kelders, Department of Psychology and Communication of Health and Risk, Faculty of Behavioural Sciences, University of Twente; Bart Brandenburg, Medicinfo;Erwin Seydel, Department of Psychology and Communication of Health and Risk, Faculty of Behavioural Sciences, University of Twente

Evaluation of the use of an interactive web-based support program for optimizing the management of Diabetes Mellitus

(Research)

Background: The introduction of the Internet into medical practice as an information-sharing and communication medium has brought about many opportunities for the management of chronic care. Research has shown that teleconsultation for example is a practical, cost-effective, and reliable way of delivering a worthwhile health care service to diabetics [1,2]. Medicinfo, a leading e-health company in the Netherlands, used this knowledge to develop an interactive web-based diabetes self-management support program, called the Diabetescoach: a low-tech solution for a large group of diabetes patients (type II). The Diabetescoach is aimed at improving access to care by facilitating easier, time-efficient communication between patients and nurses, and at facilitating self-care via education, monitoring, and entertainment for persuading individuals to make life-style changes. A Diabetescoach pilot project runs within three primary care practices (pilot group: 50 patients, 6 nurses). **Objective:** To assess the added value of the Diabetescoach as a supplement on regular diabetes care. We focused on patient needs, expectations, motivations, and experiences with electronic care via the Diabetescoach. We also assessed nurses' experiences.**Methods:** We conducted usability tests combined with in-depth interviews with 20 patients and 5 nurses to assess patients' and nurses' experiences with electronic care. Log-files registered system usage and content of patient-caregiver interaction. Via a validated questionnaire we gathered information about patient characteristics, like age, gender, education, diabetes duration, quality of life, diabetes knowledge, and diabetes self-efficacy.**Results:** Mean age of the participating patients (n=50) was 62 years, with a minimum age of 43 years and a maximum of 80 years. Log-files showed that 80% (n=40) of the total pilot group used the Diabetescoach regularly. Usability tests and interviews revealed that the Diabetescoach is seen as a useful and worthwhile supplement on regular diabetes care. It proved to be a powerful instrument to learn about the disease and about disease control. Usability tests also functioned as a powerful training method; participants were more skilled and more motivated to use the Diabetescoach afterwards. Patients were particularly interested in telemonitoring and the mail contact with their caregiver. Nurses often initiated mail contact; they give feedback on measurements, lifestyle, and make compliments on patients' healthy behaviour. Patients ask for information about nutrition, for personal advice on health issues, they use mail for changing appointments, and for expression of emotions (worries, appreciation about care delivery). Patients like to receive feedback and compliments by mail; it motivates them to adapt to healthy behaviour. **Conclusion:** The Diabetescoach supports patients in the control of their own care. Patients are particularly enthusiastic about the possibility to mail with their nurse. However, not all patients were motivated or skilled enough to use the Diabetescoach. For electronic care applications, it is important to give adequate training and to explore who suites which technology best and what changes are necessary to reach non-users or drop-outs. Innovations in health care diffuse more rapidly when technology is used that is simple to use, affective aimed at expression of emotions and useful; taking into account the expectations and needs of end-users [3].

1. Verhoeven F, van Gemert-Pijnen L, Dijkstra K, Nijland N, Seydel E, Steehouder M. The contribution of teleconsultation and videoconferencing to diabetes care: a systematic literature review. J Med Internet Res 2007 Dec 14;9(5):e37.
2. Glasgow RE, Boles SM, McKay HG, Feil EG, Barrera M Jr. The D-Net diabetes self-management program: long-term implementation, outcomes, and generalization results. Prev Med 2003 Apr;36(4):410-9.
3. Cain M, Mittman R (2002). Diffusion of innovation in health care. California HealthCare Foundation, Institute for the future. URL: http://www.iftf.org/docs/SR-778_Diffusion_of_Innovation_in_HC.pdf

1:30 PM - 3:00 PM in MaRS CR2

Chair: Aviv Shachak

Topic: Semantic Web ("Web 3.0"), Open Source

Julio Lorca, Fundacion para la eSalud-FESALUD, Malaga, Spain; Francisco J Alcazar , Consejeria de Innovacion, Ciencia y Empresa, Andalusian Government, Malaga, Spain; Carlos L Sanchez , Consejeria de Innovacion, Ciencia y Empresa, Andalusian Government, Malaga, Spain; Miguel Romero-Cuevas , Fundacion para la eSalud-FESALUD, Malaga, Spain; Diego L Lopez , eHealth Competence Center, Regensburg, Germany; Sergio Ruiz , Revista eSalud, Spain; Pedro Garcia-Fortea, Fundacion para la eSalud-FESALUD, Malaga, Spain

PESCA: Developing an Open Source Platform To Bring eHealth To Latin America and the Caribbean

(Research)

No abstract available

Jen Mccabe Gorman; Maarten Den Braber, University of Twente, Healthcare Technology and Management

"Nexthealth" - An Open-Source, Web-Based Tool Determining "What's Next" in the Evolution of Consumer-Centric Care Delivery

(Practice)

Semantic web technology and open source, 'free range' APIdevelopment provide opportunities to build consumer-centricservices beyond current Health 2.0 strata. But the roadmap for true consumer-centric care does not end with semantic web growth –rather it begins there.

The authors detail a new planning model, termed 'neXthealth,'giving stakeholders concrete tools to define current positioning and create a map to future goal realization (using existing service lines and developmentinitiatives). We focus on four critical factors needed to realize complete consumer-centric care (aka 'nexthealth'): content + community + commerce + coherence.

The authors plot the roadmap to consumer-centric care at the intersection of two rapidly evolving developmental axes: joining patients andprofessionals in the healthcare conversation, and combining brick-and-mortar real world systems with virtual online services. A case study is used to illustrate the ideal result: completely integrated care delivery where consumers may access healthcare goods and services at will, online or offline.

American Well, a Health 2.0 company included in a case study in the presentation, recently validated the nexthealth theory, partnering with Microsoft and the State of Hawaii to deliver the 'online healthcare marketplace' paid for by insurance providers.

A live, web-based Flash demo of the neXthealth model will allow Medicine 2.0 attendees to participate in a crowd-sourcing experiment, plotting points on the roadmap to 'nexthealth' and connecting the dots to determine 'what's next' for consumer-centric care, according to the Congress.

The authors present not a commercial, one-size-fits all solution to consumer-centric care evolution, but rather a 'killer planning app' - a practical, graphical, open-source tool for executive teams to determine 'what's next' for each organization. In the spirit of crowd-sourcing healthcare evolution, the model will be available free online after launch.

Charalampos Bratsas, Lab of Medical Informatics, Medical Faculty, Aristotle University, Thessaloniki, Greece; Panagiotis D Bamidis, Lab of Medical Informatics, Medical Faculty, Aristotle University, Thessaloniki, Greece; Evagelos Kaimakamis, Lab of Medical Informatics, Medical Faculty, Aristotle University, Thessaloniki, Greece; Nicos Maglaveras, Lab of Medical Informatics, Medical Faculty, Aristotle University, Thessaloniki, Greece

Usage of Semantic Web Technologies (Web-3.0) Aiming to Facilitate the Utilisation of Computerized Algorithmic Medicine in Clinical Practice

(Research)

Introduction: Despite the proven benefits of algorithmic medicine in healthcare and the plethora of implemented medical algorithms solving Medical Computational Problems (MCPs) which are available on the web [1], their usage is limited in everyday clinical practice. This is mainly due to poor organisation of MCP information, difficulties in MCP search and the missing parameters in the description of medical cases, making their management by a single algorithm rather impossible. In this paper a comprehensive approach to the usage of semantic web technologies (web-3.0) is presented, aiming to facilitate the utilisation of computerized algorithmic medicine in clinical practice. In particular, there are 3 main goals achieved, namely, the semantic descriptions of MCPs, the efficient search of MCPs, and the dynamic semantic composition of a sequence of algorithms managing a certain medical case. **Methods**: For the Semantic representation of MCP knowledge the MCP OWL Ontology describes the MCPs as a triad:

(Medical Problem-Algorithmic Solution-Implementation)

Three interacting ontological schemes that refer to each part of the above triad were created.

For efficient search, the method used is an adaptation of the classical Vector Space Model (VSM) in MCP Ontology, via which the similarity between MCP semantic descriptions and the user questions is calculated. The weights of MCP vectors are created utilizing the UMLS Ontology [2].

In order to dynamically composite a pathway of algorithms for managing a certain medical case SWLR semantic rules are used. These rules automatically associate different algorithms and construct a Finite State Machine (FSM) of algorithms. The description of a certain medical case via the MCP Ontology by a user constitutes the "language" for that case. If this language is recognised by an FSM of algorithms with the final algorithm that manages the case as the initial state and the algorithm of initiation by the user as the final state, then the sequence of these algorithms can manage the medical case.**Results:** A modular and expandable, Web-Based Knowledge System (KS) for the MCPs was developed. Preliminary results from its usage showed a more efficient search of MCPs, as well as, a proper management of medical cases through algorithmic

pathways proposed by the system which were in agreement with international medical guidelines whenever these were available.**Discussion:** The developed methods of display and management of MCP knowledge along with the further utilization of the proposed KS are expected to enhance the dissemination and use of algorithmic solutions in everyday medical practice. Simultaneously, medical research and high quality medical education are bound to be benefited at a considerable level. For the dissemination of algorithmic medicine we believe that the future is the combination of web-2.0 and web-3.0 technologies and the transformation of our system to a semantic wiki of MCPs.

[1] Iyengar MS, Svirbely JR: Computer-based medical algorithms: overview and experiences. Technol. Health Care 2005, 13(5):403-405

[2] Bratsas C, Koutkias V, Kaimakamis E, Bamidis PD, Pangalos G, Maglaveras N: KnowBaSICS-M: An ontology-based system for semantic management of medical problems and computerised algorithmic solutions. Comput. Meth. Progr. Biomed 2007, 88(1):39-51

1:30 PM - 3:00 PM in MaRS CR3

Chair: Trevor van Mierlo

Topic: Web 2.0 approaches for clinical practice, clinical research, quality monitoring

Hughes Benjamin, Dept of Information Systems, ESADE, Barcelona, Spain; Indra Joshi, North and East Hertfordshire PCT Trust; Hugh Lemonde, Department of paediatrics, King George Hospital, London

To 2.0 or not to 2.0 – have junior doctors already answered the question?

(Research)

Background: Use of Web 2.0 by doctors has been demonstrated by surveys both in the UK [1] and the US [2]. Furthermore, many authors have called for a wide awareness of these technologies and further research into their use [3]. However despite this research, the surveys only indicate use over extended time frames, and we have little understanding on if and how this impacts daily clinical practice **Objective:** This paper aims to determine the impact of Web 2.0 in daily clinical practice in the UK, understanding its frequency of use, both absolute and in comparison to traditional internet resources aimed at doctors **Method:** A sample of 30 ST1-level (2 years post registration) UK NHS doctors across a range of specializations recorded a daily diary of internet use over a week. The sample was selected from London medical schools graduate cohort. The selection of junior doctors at the ST1 grade was deliberate, based on the fact that research has shown that use of Web 2.0 decreases with a doctor's seniority [2]. This paper seeks to provide a potential vision of how doctors will use Web 2.0 going forward through their training. Diaries were selected as a method for their effectiveness in examining use of time and collecting data from the perspective of the participant [4]. In addition, participants took a survey of attitudes to Web 2.0 technologies as completed in previous surveys [1] to determine any bias in the sample.**Results:** Early results indicated that Web 2.0 tools are more widely used than other internet tools (53% of total internet use) with on average being used 2 out of every 5 days. Wikipedia was the most popular tool, representing 20% of total internet use being used by 79% of users. This was followed by Google at 15% of total internet use. 83% of respondents indicate that their use of these tools was due to being able to find relevant information more efficiently than other sources (internet resources of reference books in the ward or clinic). Significant differences in attitudes to technology between this sample and larger young doctor populations examined in other surveys could not be established.**Conclusion:** A sample of 30 doctors cannot be taken as representative of the whole population of young UK doctors or UK doctors in general, but it suggests that adoption is beyond that noted by previous research. This supports the academic literature that has identified the potential of Web 2.0 resources to clinical practice [5]. Furthermore, as noted by Web 2.0 definitions, users can rapidly decide if tools are useful or not driving adoption more than organization policies or promotion of tools. Have junior doctors already answered the question, to 2.0 or not to 2.0?

References

1.Sandars J, Schroter S. Web 2.0 technologies for undergraduate and postgraduate medical education: an online survey. Postgrad. Med. J. 2007;83;759-762. doi:10.1136/pgmj.2007.063123. PMID: 18057175

2.Manhattan Research, LLC. White Paper: Physicians and Web 2.0: 5 Things You Should Know about the Evolving Online Landscape for Physicians. 2007. URL: http://www.manhattanresearch.com/TTPWhitePaper.aspx on 20/1/2008

3.Potts HW. Is e-health progressing faster than e-health researchers? J Med Internet Res. 2006;8(3):e23 PMID: 17032640

4.Easterby-Smith, Thorpe, Lowe. 2002. Management research: An Introduction. Sage:London

5.McLean, R, Richards, B, Wardman, J. The effect of Web 2.0 on the future of medical practice and education: Darwikinian evolution or folksonomic revolution? MJA 2007; 187 (3): 174-177. PMID: 17680746

Miguel Cabrer, eHealth Advisor

MDPIXX: The Global Medical Images Repository

(Practice)

MDPIXX (http://mdpixx.com) is a multi-media portal for clinical cases, medical images and videos – a convenient way to upload and share medical images and associated information. Physicians, researchers and educators can use MDPIXX within their institution or collaborate and interact with colleagues anywhere in the world for clinical, research, teaching and decision

support purposes. MDPIXX is free and exclusively for physicians.

MDPIXX can be used for images management, collaboration and sharing purposes, organize clinical cases from an organization, store physicians medical images, second opinion, telemedicine, teaching purposes and decision support.

SNOMED CT for Terminology

MDPIXX allows to organize medical media like images and videos. Also, Clinical Cases can be created with a title and description. All the resources and cases use the keywords system to categorize the content. Keywords are based on SNOMED CT Terminology which allows MDPIXX to index cases and images, relate clinical information, link to external sources and perform automatic translation of the keywords depending on the user default language selection. SNOMED provides a battery of more than one million clinical terms.

Powerful video management

MDPIXX allows to manage big videos and images. Big videos can be uploaded to MDPIXX and will be coded correctly to be seen and broadcasted on the internet.

Vote, comment and share

MDPIXX is a web 2.0 site so a social community is going to manage and control the content. Cases, images and videos are valued by the MDPIXX users, commented and shared with other users.

White Label MDPIXX: use a personalized MDPIXX for your organization

MDPIXX is happy to partner with hospitals or medical centers that want to use MDPIXX. MDPIXX can be used as white label product. The mash-up embedded technology allows health organizations to use MDPIXX personalized and privately only for the organization community or workgroup. So any Telemedicine network or health organization can have their MDPIXX space with their own logo, users and content. You can also embed MDPIXX related cases and content into your intranet site or Electronic Medical Record application.

Some partners have already established a relationship with MDPIXX to define a worldwide private telemedicine network, second opinion businesses, research institutes, special illness repository, health support to non developed countries, etc.

Marcelino Cabrera Giraldez, Institute for Prospective Technological Studies (Joint Research Centre, European Commission); José Antonio Valverde , IPTS; Dolores Ibarreta, IPTS

Social Computing Enhances Biomedical Research on Rare Diseases

(Research)

Subject: Results of our ongoing Study on the Potential of Social Computing for Biomedical Research in Rare Diseases.**Rationale of our study:** Rare Diseases (RDs) affect 6-8 % of the European population, approximately 25 million citizens. There exist between 5000 and 8000 RDs of which 80 % have a genetic origin. Because of their rarity, these diseases are hardly observed in basic diagnostic procedures and pathways by clinicians, resulting in under-diagnosing and/or on longer waiting periods needed to get the correct. Research on rare diseases (RDs) has traditionally been hindered by the fact that cases, clinicians, researchers and resources (pharmaceutical companies have lacked return on investment) are scattered. One of the key characteristics of social computing is its ability to enable powerfully user-created content. Social Computing, also known as the Web 2.0 has the potential to connect up all the actors and stakeholders, especially patients and biomedical researchers. These activities and networks are already allowing a critical mass of knowledge to be gathered, from both patients and researchers - albeit in an unstructured manner.**Objectives:** To assess the capability of social networks to enable sustainable research on rare diseases, by allowing patients to be knowledge generators, in synergy with biomedical researchers, and to propose a structured construct to dramatically increase this synergy. Specific objectives of our research include: a) the assessment of opportunities and challenges of social networks for research, from the point of view of patients, formal and informal carers, clinicians, researchers, industry and society; b) a proposal for a construct to structuring and making efficient this potential; c) the derivation of policy options at EU level as to develop this construct; d) the analysis of relevant implications for privacy and security of social computing related activities in this realm.**Applied method:** We have applied a 3-step methodology: 1) Browsing current experiences (there are scarce but there are some examples, e.g. Autism: IAN Project http://www.ianproject.org/ and OAR http://www.researchautism.org/); while specifically checking the relevant social computing-based applications, if any. 2) Holding an expert workshop to ascertain the opportunities and challenges; and 3) proposing a structure for this construct, partly based on Science 2.0 theories and also on some practical cases.

Preliminary conclusions: Though RDs affect only 6-8% of European citizens, Information Society Technologies platforms using social computing approaches have a considerable potential for research on RDs, not only as regards its sustainability but also its profitability for both the pharmaceutical industry and the society at large. The body of knowledge on RDs has developed very slowly and is still largely an "'uncharted territory". Based on the "Long Tail" theory [1], research on specific rare diseases through the application of social computing is worth - socially, clinically and economically. Cases examined preliminarily confirm our hypothesis. An international expert consultation will serve to systematise and validate our insights so far.

References:

1. Anderson, Chris. The Long Tail – Why the Future of Business is Selling Less of More. Hyperion, New York. 2006.

2. RDCRN – Rare Diseases Clinical Research Network. http://rarediseasesnetwork.epi.usf.edu/

3. Sauer I, Bialeck D, Efimova E, Schwartlander R, Pless G & Neuhaus P. \'\'Blogs\'\' and \'\'Wikis\'\' are valuable software tools for communication within research groups. Artificial Organs 29(1):82-89, Blackwell Publishing, Inc. 2005.

4. Singer, E. Social Networking Hits the Genome. http://www.technologyreview.com/Biotech /20464/

5. Waldrop, M. Science 2.0 – Is Open Access Science the Future? Scientific American Magazine – April 21, 2008. (Also available at http://www.sciam.com/article.cfm?id=science-2-point-0).

Chris Paton, Health Informatics Programme, Department of Information Science, University of Otago, Dunedin, New Zealand; Muzaffar Malik, Health Informatics Programme, Department of Information Science, University of Otago, Dunedin, New Zealand; Alec Holt, Health Informatics Programme, Department of Information Science, University of Otago, Dunedin, New Zealand

Mobile Medicine 2.0

(Research)

Introduction A new generation of smartphones is enabling high speed internet access via the mobile web browser. In this paper, we examine the unique aspects of using social media applications on mobile platforms and suggest potential uses for medical practice. Personal Digital Assistants (PDAs) have long been used by clinicians as reference tools(1) and simple patient databases. Recent advances in mobile phone and PDA technology have allowed for high speed access to social media web applications such as social networks, blogs and wikis.**Methods** We performed a literature review and conducted technical evaluations of current social media applications designed for access on mobile platforms. In particular we examined how these applications could be used in medical practice and identified cases where health professionals have utilised mobile access to social media applications. We also performed evaluations of smartphone devices and identified the key technologies that are enabling high-speed web access on mobile devices.**Results** Most major social media applications now have specially formatted mobile versions. Recent developments in mobile hardware include 3rd Generation (3G) high speed internet access, multi-touch displays and high resolution screens. The technologies combine to offer an improved web browsing experience that enable the use of more advanced types of website such as social networks, blogs and wikis. Although there is a paucity of available research into how these mobile social media applications can be applied to medicine, there appears to be some evidence that they could be useful for patients and clinicians. Some applications, such as micro-blogging(2), appear to be more suited to mobile use than others and may have more potential for use in healthcare. **Discussion** We propose a number of medical uses for popular mobile social media applications and examine the unique technologies and methodologies that differentiate these tools from legacy mobile technology such as bleeper systems and non-connected personal digital assistant technology. We discuss the possibility for integrating the technology and techniques used by popular consumer social media applications with recent advances in clinical coding and communication standards such as SNOMED CT and HL7. As mobile access to the electronic health records become more common, so too will mobile access to medical social media applications to support the evidence based practice of medicine.

References

1. Baumgart DC. Personal digital assistants in health care: experienced clinicians in the palm of your hand?. Lancet. 366(9492):1210-22, 2005 Oct 1.

2. Ginzberg U. Twitter: applications for the health 2.0 world. Retireved May 2 2008 from http://blog.medical20.com/2007/06/twitter-applications-for-health-20.html

3:30 PM - 4:30 PM in MaRS Auditorium

Topic: Ethical & legal issues, confidentiality and privacy

Joan Dzenowagis, World Health Organization, Geneva, Switzerland; Shubhabrata Roy, Microsoft Corporation; London; Kevin Clauson, Pharmacy Practice, Nova Southeastern University, Florida

Risk 2.0: The future of connected health (Panel)

(General)

Over the past decade the Internet has radically changed the dynamics of health, bringing not only new ways of providing information and care for individuals, but also for communicating and managing health matters on a global basis. New applications, services and access to information have permanently altered the relationships between consumers, health professionals, industry and governments – and there are more changes yet to come with Health 2.0. One of the tenets of Health 2.0 is choice, where consumers take a more active role in their health. Despite the proliferation of wikis, blogs and a host of websites dedicated to their particular concerns, nevertheless there remains an inherent information asymmetry between health care providers and consumers. Bridging these differences presents enormous challenges and goes beyond simply providing tools for consumers, towards facilitating their effective use for improving health outcomes. As significant and well-publicized incidents have shown, there is increasing public pressure on professionals, as well as on national and local systems to better manage the consequences of the online world. These pressures arise in part from the public's perception of risk, such as theft or disclosure of personal health records or vulnerability to fraud and misinformation, to cite several examples. This panel draws on research and expertise in new technologies, health communication and international public health to explore the public's understanding and perception of quality, evidence and risk. Using an audience response system to facilitate discussion, participants will highlight real and perceived dangers of online health and the implications for the sector. Together the panel and audience will consider the role of consumers, health professionals, industry and governments in facing the challenges of the next decade as the technologies evolve and as our ability to understand, prevent and manage risk improves.

Poster Presentations (Foyer)

Thursday 10:30 AM – 11:00 AM

Poster 1

Clara Bermúdez-Tamayo, Spain;Jaime Jiménez-Pernett, Andalusian School of Public Health; Jose Francisco Garcia-Gutierrez

Using Web 2.0. tools on health Websites in Spanish

Objetive To evaluate the use of Web 2.0 tools on health websites in Spanish. **Methods** We conducted a descriptive study on the tools of Web 2.0. used by health websites. We review 39 websites on general health in Spanish, and 32 Web sites of specific areas (including allergy, infectious and parasitic diseases, digestive system, endocrine system, nutrition, metabolic diseases, circulatory system, apparatus genito-urinary, respiratory, mental illness, musculoskeletal system and connective tissue, nervous system, rare diseases, back, older and disabled, women's health, perinatal health, tumors and vaccines). Websites were selected in a previous study. We analyzed the following dimensions (Cobo-Romany 2007): Social Networking (Forums, Communities), content management (Blogs, Wikis, space for comments and opinions), Social organization of information (RSS, bookmarkings, informative panels) and applications and services (podcasting, webcasting). **Results** Social networking. 31% of all health websites in Spanish has used social networking tools, including forums with specific applications developed for each site. Content Management System (CMS): The tools designed to create and manage content on the health websites are scarce being used in less than 10% of all; Blogs = 5, Wiki = 1, Comments and opinions = 7 . Social organization of information: The RSS feeds are the most widely tool used on Web sites evaluated (25%), on bookmarkings, there are 6 Websites that let you share its contents employing various open management services as Del.icio.us, Digg, Technorati, among others. There are 9 websites that use information panels. Only two Websites of media used all the tools. Applications and services. Only 13% of Websites evaluated offer the ability to play or download audio and video (Podcasting and webcasting). **Conclusions** Websites with health information in Spanish have not yet migrated to the Web 2.0. The media are the websites that most have used the tools of Web 2.0.

Poster 2

Jaime Escallon, Toronto, Canada; Adriana Fonseca

Email Communication Between Physicians and Patients

Nowadays an internet connection is available almost everywhere. In Canada, 67.9 %[1] of the population have access to Internet and 91 %[2] of the Internet users have an email account. When improving the communication between physicians and patients is a priority, email communications become an excellent tool to provide an efficient contact with the physician. **Objective:** The objective of this study is to evaluate the content of emails exchanged between physician and patients in a Breast surgery setting.Methods: Retrospective analysis of the content of random emails collected for a physician in a breast surgery setting, for the last three years. A small sample (67 emails) has been collected; we analyzed and coded the content and the length of the emails.**Results:** 67 Emails were analysed, 99 issues were identified, 60% of the emails were sent before surgery and the other 40% were sent after surgery, 54.5% of the patients sent one email, and 77.61% were sent by the patient. In the 50.49% of the emails, the issue was General information. The average length of the emails was 146 words per email, the literature reports the average adult can read approximately 120 words per minute, so email communication could be a cost-effective method of communication between patients and physicians. With the results of this study, we will be able to educate the patients and the physicians in the use of the email as part of the regular patient care in our institution and start a new prospective study with more statistical significance.

REFERENCES:

1. Statistics Canada. Characteristics of individuals using Internet. 2008-05-02. URL:http://www40.statcan.ca/l01/cst01/comm15.htm. Accessed: 2008-05-02. (Archived by WebCite® at http://www.webcitation.org/5XWJyZCKr)

2. Statistics Canada. Internet use by individuals, by type of activity. 2008-05-02. URL:http://www40.statcan.ca/l01/cst01/comm16.htm. Accessed: 2008-05-02. (Archived by WebCite® at http://www.webcitation.org/5XWKMtS9B)

Poster 3

Hanberger Lena, Diabetes Research Centre, Linkoping University Hospital, Sweden; Nordfeldt Sam, Diabetes Research Centre, Linkoping University Hospital, Sweden; Larsson Anders, Dept. of Computer and Information Science, Linköping University, Sweden

Information search behaviours in a controlled study of a web portal for young diabetes type 1 patients and their parents

Introduction: We report pilot data on information search behaviours in a web portal targeted for young type 1 diabetes patients and parents. The portal www.diabit.se was designed in a user-centred process and contained comprehensive specific diabetes-related information, pedagogic devices and social networking tools. The navigational structure was designed as a 2-level system, where users always can access all the main menues. Second level menues are only visible at specific locations within the site after choosing a main

menu. Thus the site gives equal access to each of the following:

Main menues on Diabetes-related information are Acute situations, What is diabetes, Relations, Late complications, Insulin, Devices, Food, Blood glucose, Exercise and sports, Living with diabetes, This can affect, Research and External links (containing altogether 85 second level pages). Other main links are Home, About Diabit, Rules, Videos, Simulator, Discussions, Stories, Diary, Wordbook, Blog, FAQ, Team Jönköping and Team Linköping.

The frontpage is dynamic with regularly updated news and links to varying contents, e g linking to Team Jönköping and Linköping respectively, and includes an internal search engine. **Method:** Out of a geographic population of children and adolescents with parents treated at two pediatric clinics in Jönköping and Linköping, randomly selected families were given personal logins to www.diabit.seDuring a 15-months pilot use, with informed consent from each user at first login all page visits were logged. **Results:** 74 unique mothers, 40 fathers and 46 adolescents aged 12-18 years were active users. The mother/father ratio was 74/40 in unique users, 3423/1325 in page hits. The adolescent/parents ratio was 46/114 in users and 1611/4748 in page hits. The total numbers of page hits were 6359, of which 1611 in adolescents (range 1-168), 3423 in mothers (range 1-314) and 1325 in fathers (range 1-253) respectively. Page hits per weekday were Mon 1193, Tue 831, Wed 1376, Thu 956, Fri 801, Sat 541 and Sun 661. Summer holiday periods generated the lowest visit frequencies.

The most frequently visited pages will be described in terms of page hits and % of hits per usergroup, form the pages receiving 0.9% of all hits and higher, with social networking highlighted.**Discussion:** Patients and parents in families with type 1 diabetes children actively explored the presented web portal, especially on clinical visit weekdays. Interestingly, more frequent page hits were seen on social networking such as blogs, stories and discussions. Thereafter, the local diabetes team pages and an online disease simulator device generated most frequent hits.

The findings might support introducing such functions into routine care, in close collaboration with patients and health professionals. More knowledge is needed on application of online social networking facilities and pedagogic devices in real-world disease-specific clinical practice.

Poster 4

Maria Inmaculada Grau, Hospital Clinic de Barcelona; Joan Gene-Badia, CAPSE-Universitat de Barcelona; Encarnacion Sanchez Freire, Fundacio Clinic per a la Recerca BIomedica; Miguel Bernardo, Hospital Clinic de Barcelona-Universitat de Barcelona

Is www.forumclinic.org an electronic support groups for patients with chronic illnesses?

Introduction: In January 2007 forumclinic (http://www.forumclinic.org) was launched. It is a Website produced in both Spanish and Catalan, with the objective of improving the quality of life of chronically ill patients. It allows patients to participate in a discussion forum moderated by health professionals. Barry Wellman defines virtual community as networks of interpersonal ties that provide sociability, support, information, a sense of belonging, and social identity. **Objective:** To assess if forumclinic has developed a virtual community as a first step to evaluating its effectiveness in delivering social support electronically. **Methods:** Web has forums for schizophrenia, diabetes, COPD, ischemic heart disease and breast cancer. During weeks 14 to 17 of 2008 we measured using qualitative and quantitative methods: web and forum audience, characteristics of forum visitors and participants, adherence to forums and topics of interest. **Results:** 1-web has received 32.364 visits during this weeks, 2-27.9% visit forums, 3-4.7% of this participate. 4-An average of 0.2 post/topic in breast cancer forum and a 3,4 in COPD forum5-Females are the highest participations in forums (60,5% about participations)6-In breast cancer forum participants are mostly patients (76%) and relatives participate more in the schizophrenia one (46%) than in the other forums. 7-Moderator posts are 49% in the esquizophraenia forum, 26% in breast cancer and 4% in COPD.**Conclusion:** Our data suggests that we have succeed in developing virtual communities of chronic patients although only the COPD and breast cancer forums can be considered electronic support groups. forumclinic seems to be an useful tool of bidirectional communication between health professionals and citizens as well as for disseminating scientific knowledge to the general public, dealing with scientific evidence as well as the emotional aspect of healthcare.

Poster 5

Saskia M. Kelders, University of Twente; Nicol Nijland, University of Twente; Marieke S. Beld, University of Twente; Julia E.W.C. Van Gemert-Pijnen, University of Twente; Mariët Theune, University of Twente; Bart Brandenburg, Medicinfo; Erwin R. Seydel, University of Twente

Using usability-tests to improve language match of a web-based system for digital triage in primary care

Background: Previous research showed that health care consumers favor web-based communication systems to control their own care [1, 2]. Systems that combine high-quality information with interactive components for self-assessment, decision support, or behavior change have the potential to reduce costs while maintaining the same or achieving better quality of care [3]. A system that incorporates these different features is eConsult|Plus (developed by Medicinfo) which combines digital triage with e-consultation. For digital triage systems to be adopted and accepted, it is important to adjust the system to the needs and expectations of its users. A serious problem hereby is a discrepancy between the language of users and that of the system (inadequate language match) [4].

The system under investigation matches the description of the complaint entered by users to pre-defined complaints in a database. This matching occurs by comparing the entered description to a list of synonyms of the pre-defined complaints. The matched complaint is linked to questionnaires which provide triage and identify the urgency. The users receive a computer generated advice, varying from "contact a doctor immediately" to sending their GP a question accompanied by the gathered information. **Objective:** The aim of this study was to assess and improve the language match of a web-based system for digital triage in primary care in the Netherlands.**Methods:** A usability-test (55 consults by 19 unique respondents) was conducted to assess the language match of the web-based system and to obtain insight in the users' language. These data were used to complement and adapt the database. An online survey (29 different respondents) was conducted to obtain additional synonyms. Finally a second usability-test (60 consults by 20 different respondents) was conducted to measure the effects of adapting the database.**Results:** The initial usability-test revealed that the system returned a response in 58,1% of cases (no response means the system asks the user to rephrase the complaint) of which only 64,5% included an adequate response. On average respondents needed 2,3 descriptions to get an adequate response. The average number of matching-problems (problems that occur during the description process and the system's response) was 5,2. Overall, participants awarded the system with 6,23 (scale 1-10). The second usability-test showed significant improvement in all areas: 82,4% response, of which 91,0% included an adequate response; users only needed 1,42 descriptions to get an adequate response (p=0,001); users experienced 2,42 matching-problems (p=0,000); the overall grade increased to 6,87 (p=0,004).**Conclusions:** Altering and expanding the database of the system by making use of the actual language of users, significantly improved the language match, resulting in more adequate responses, fewer descriptions needed, less problems experienced and respondents valuing the system higher. This research shows that usability-testing is an effective way of matching the language of systems and their users and that improved matching not only leads to fewer problems, but also to higher ratings and eventually to improved adoption and acceptation of these systems.

References

1. Katz SJ, Moyer CA. The emerging role of online communication between patients and their providers. J Gen Intern Med 2004 Sep;19(9):978-83.

2. Eysenbach G, Sa E R, Diepgen T L. Cybermedicine. Interview by Clare Thompson. BMJ 1999 Nov 13;319(7220):1294.

3. Kerr C, Murray E, Stevenson F, Gore C, Nazareth I. Internet interventions for long-term conditions: Patient and caregiver quality criteria. J Med Internet Res 2006 Jul;8(3):e18.

4. Barry CA, Stevenson FA, Britten N, Barber N, Bradley CP. Giving voice to the lifeworld. More humane, more effective medical care? A qualitative study of doctor-patient communication in general practice. Soc Sci Med 2001 Aug;53(4):487-505.

Poster 6

Veronica Law, Graduate Department of Rehabilitation Science, University of Toronto

Internet Use among Occupational Therapists in Their Clinical Practice

Introduction There has been an increased use of the Internet in clinical practice for a broad range of health care professionals; however, this has not yet been documented for persons working in the rehabilitation field.**Objectives** This study aimed to investigate the patterns of Internet use in clinical practice among occupational therapists. **Methods** A self-administered, structured survey was mailed to all provincially registered occupational therapists (n=4050) who engaged in any form of client contact (e.g., personal interaction or phone consultation). Descriptive statistics were used to describe their Internet use and bivariate data analyses were performed to identify relationships between therapists' characteristics and Internet use. Barriers and facilitators to Internet use (e.g., paid time) were also examined.**Results** Of the 1382 surveys received that met the inclusion criteria for data analysis; majority of respondents were female, full-time therapists who lived and worked in urban areas. A majority of participants reported that access to information over the Internet was useful as their clinical information source. The top 3 facilitators for Internet use were: 1. "having a computer at work", 2. "a work culture that fosters Internet use" and 3. "the availability of technical support". To a lesser extent, having paid time to access the Internet and the presence of regulatory guidelines (e.g., privacy issues related to emailing clients) were

also reported as influencing the therapists' Internet use in their clinical practice. **Conclusions**The identification of facilitators and barriers faced by clinicians is an essential step when looking for ways to encourage the adoption of technology in the delivery of health care services (e.g., using accessible Internet information as materials for patient education). The results seem to signal a need for additional funding for technical training and guidelines for email use in a clinical setting. The study findings also include factors that may facilitate greater use of the Internet for clinical practice among occupational therapists that may also be relevant for other health care professionals.

Poster 7

Nordfeldt Sam, Diabetes Research Centre, Linkoping University Hospital; Hanberger Lena, Diabetes Research Centre, Linköping University Hospital; Timpka Toomas; Nordqvist Cecilia

Health professionals' attitudes toward using an Internet portal for child and adolescent diabetes care

Introduction: Internet-based interventions in diabetes care may improve patients' health care utilization, behaviour, attitudes, knowledge and skills, but have reached little clinical application in pediatric diabetes care. This study aimed to explore health professionals' baseline attitudes and expectations when starting to use a tailored Internet-based information system in their clinical practice with young type 1 diabetes patients. **Methods:** After participating in a user-centred process for designing a web portal containing targeted diabetes-related information, pedagogic devices and social networking tools, 20 diabetes professionals from two paediatric diabetes teams underwent a semi-structured interview by a sociologist. Using a modern phenomenological method, she analysed the interviewees´ experiences within the context of culture.**Results:** Health professionals expressed positive expectations regarding integration of the targeted portal into routine care. A complementary role was suggested for the portal, where patients would be able to search for information before, in between and after scheduled visits and telephone contacts. Whereas some respondents expected that e-mail communication with patients and online patient information would save time in routine care, others emphasized the need of face-to face communication. Online peer-to-peer communication was mentioned as an important function too, but most interviewees did not expect the portal to be used very much for social networking. There were no major differences in attitudes between different professions or clinics, but some differences appeared in relation to work tasks.**Discussion:** Health professionals in clinical diabetes teams, irrespective of profession, are in favour of implementing targeted Internet-based resources for young patients with type 1 diabetes. The findings might support introducing these into routine care in close collaboration with patients and health professionals. More knowledge is needed on application of online social networking facilities in real-world disease-specific clinical practice settings.

Poster 8

Cornelia van Uden-Kraan, Institute for Behavioural Research, University of Twente, Enschede, The Netherlands; Constance Drossaert, Institute for Behavioural Research, University of Twente, Enschede, The Netherlands; Erik Taal, Institute for Behavioural Research, University of Twente, Enschede, The Netherlands; Erwin Seydel, Institute for Behavioural Research, University of Twente, Enschede, The Netherlands; Mart van de Laar, Institute for Behavioural Research, University of Twente, Enschede, The Netherlands

Determinants of engagement in face-to-face and online patient support groups

Background Engagement in face-to-face and online peer support can have a profound effect on the participants' feelings of 'being empowered' [1, 2]. However, studies showed that many face-to-face peer support groups have encountered less than enthusiastic participation [3]. Although it was anticipated that engagement in peer support would increase by the emergence of online peer support, recently indications are found that only a limited number of patients make use of this type of peer support [4]. Objective We explored factors that facilitate or hamper engagement in face-to-face and online peer support. The Theory of Planned Behavior (TPB) [5] was used to explain patients' participation behavior. The TPB proceeds from the assumption that human behavior is determined by intentions. The intention to engage in peer support is determined by patients' attitudes, subjective norms and extent of perceived behavioral control towards peer support. **Methods** We sent a questionnaire to a random sample of patients with arthritis, breast cancer and fibromyalgia who were under treatment in a regional hospital. The questionnaire was accompanied by a letter of the patients' physician. The overall total response rate was 69% (N=679). The questionnaire included questions on demographics, engagement in peer support, and TPB variables. TPB variables were measured regarding both face-to-face and online peer support. All TPB items were measured on 5 point scales and derived from literature and earlier studies on peer support [1]. **Results** The majority of the respondents were women (84%) with a mean age of 54 years (SD 12.8 years). Patients were diagnosed with arthritis (40%), fibromyalgia (31%) or breast cancer (29%). The respondents were diagnosed 7 years ago on average (SD 7.8 years). In total 14% of the respondents had ever engaged in face-to-face meetings for patients. Only 4% of the respondents had ever had contact with peers via the Internet. On average respondents were moderately positive about participating in face-to-face (mean=3.7) and online peer support (mean=3.2). They

had much confidence in their ability to engage in face-to-face (mean=4.1) and online peer support (mean=3.9). Nevertheless, the respondents' intention to engage in peer support in the future was slightly negative. The majority of the respondents did not intend to look for peers via the Internet (71%) or face-to-face (58%) during the coming year.

A hierarchical multiple regression analysis showed that the total amounts of explained variance of intention were moderate (respectively 33% and 31%). All TPB variables appeared to be significant independent predictors of intention towards online peer support (β's varied between -.09 -disadvantages- to .20 -advantages-). Attitude (β=.23), perceived advantages (β=.17), perceived disadvantages (β=-.16) and subjective norm (β=.21) appeared to be predictors of intention towards face-to-face peer support. Of all background variables only age (β=-.24) could significantly improve the total amount of explained variance of intention towards online peer support. **Conclusions** Our study showed that only a small share of the patients made use of peer support. TPB variables could only moderately explain intention. Age and perceived behavioral control did predict online peer support, but did not predict face-to-face peer support.

References

[1] Van Uden-Kraan CF, Drossaert CHC, Taal E, Shaw BR, Seydel ER, Van de Laar MAFJ. Empowering processes and outcomes of participation in online support groups for patients with breast cancer, arthritis and fibromyalgia. Qual Health Res 2008;18:405-17.

[2] Buchanan H, Coulson NS. Accessing dental anxiety online support groups: An exploratory qualitative study of motives and experiences. Patient Educ Couns 2007;66:263-69.

[3] Winefield HR, Coventry BJ, Lewis M, Harvey EJ. Attitudes of patients with breast cancer toward support groups. J Psychosoc Oncol 2003;21(2):39-54.

[4] Van Eenbergen MCHJ. [Use of the Internet by cancer patients.] Eindhoven, The Netherlands: Comprehensive Cancer Centre South, 2006. [Report in Dutch]

[5] Ajzen I. The theory of planned behavior. Organ Behav Hum Dec 1991; 50:179–211.

Poster 9

Robert Verheij, Neth. Inst. for Health Services Reseach (NIVEL); Lea Jabaaij, Neth. Inst. for Health Services Research (NIVEL); Khing Njoo, Dutch College of General Practitioners (NHG); Henk van den Hoogen , Centre for Quality of Care Reseach (WOK); Dinny de Bakker, Neth. Inst. for Health Services Research (NIVEL)

The EMR-scan: Assessing the Quality of Electronic Medical Records in General Practice

BackgroundThe use of electronic medical records (EMR) in general practice has spread rapidly in the last decade (more than 90% today). Traditionally, these records are primarily used for direct patient care and for administrative purposes by the practice involved. In recent years, further technical developments have made it possible to exchange EMRs or summaries of EMRs between health care providers. In the UK a 'spine' will provide summary information to other health care providers, to assist with diagnosis and care [1]. In the Netherlands a national health care information hub is being implemented, with the same objective [2]. In addition, routine primary care EMRs are increasingly used for public health monitoring in some countries [3]. The use of EMRs for the health care inspectorate are currently being considered and making EMRs accessible for individual patients is desired by many. In sum, theoretically, primary care EMRs can be used for many purposes. However, there is one condition that has to be fulfilled to make EMRs useful for all these purposes: it has to be certain that EMRs provide a true picture of the health status and treatment of all patients involved. Quality of record keeping is the key issue. **Objective**To be able to assess the quality of recording by general practices in the Netherlands, we developed and tested the EMR-scan.**Methods**The first step in doing this was to identify the most important items that have to be adequately recorded. This was done on the basis of guidelines issued by the Dutch College of General Practitioners [4], concerning the minimum dataset that has to be available at the out-of-hours service (e.g. current medication, clinical notes of last contacts, allergies). The second step was developing meaningful indicators on these items, without mixing up quality of treatment and case mix characteristics with quality of recording routines. This resulted in a set of indicators and data requirements. Examples of indicators are: % of contacts with diagnosis recorded; % patients with recorded allergies. Finally, indicators were tested on EMRs of 112,315 patients in 32 general practices (the third step). **Results** Benchmark analysis on data from these 32 practices was performed. Controlling for demographic composition of practice populations, the performance of all practices on each separate indicator was plotted in order to assess the amount of between practice variation and to determine the reference value (benchmark). Reference values were generally defined as the average of the 32 practices analysed. Practices with unexplainable large deviations from this average were regarded as bad recorders. **Conclusions** Given the increasing use of primary care EMRs for multiple purposes, there is a great need for an instrument to assess the quality of recording routines. The EMR-scan provides this instrument. The EMR-scan has been developed in order to improve recording routines of general practitioners and allied personnel. It can be applied by anyone who has access to the necessary data. It offers benchmark opportunities for groups of practices; a tool to evaluate differences between software systems; and a tool to evaluate the effects of education in recording routines.

References

[1] NHS. Spine factsheet. URL:http://www.connectingforhealth.nhs.uk/resources/systserv/spine-factsheet. Accessed: 2008-04-29. (Archived by WebCite® at http://www.webcitation.org/5XRIoAffT)

[2] NICTIZ. Better care thanks to better information. URL:https://www.nictiz.nl/?nav=home. Accessed: 2008-

04-29. (Archived by WebCite® at http://www.webcitation.org/5XRITf8Xe)

[3] The potential of electronic medical records for health service management. Fleming DM, Bartholomeeusen S, Van Casteren V, Elliott C, Verheij R. European Journal of Public Health, Vol. 16, Supplement 1, 2006, pp 104-105.

[4] NHG / NICTIZ. Richtlijn Gegevensuitwisseling Huisarts-Centrale Huisartsenpost. . 2008-04-29. URL:http://www.nictiz.nl/uploaded/FILES/WDH/Richtlijn%20Gegevensuitwisseling%20Huisarts%20en%20Centrale%20Huisartsenpost%20(CHP)_%20januari%202005.pdf. Accessed: 2008-04-29. (Archived by WebCite® at http://www.webcitation.org/5XRJLyu1p)
